# SBH17: Benchmark Database of Barrier Heights for Dissociative Chemisorption on Transition Metal Surfaces

**DOI:** 10.1021/acs.jctc.2c00824

**Published:** 2022-12-19

**Authors:** T. Tchakoua, N. Gerrits, E. W. F. Smeets, G.-J. Kroes

**Affiliations:** † Leiden Institute of Chemistry, Gorlaeus Laboratories, Leiden University, P.O. Box 9502, 2300 RALeiden, The Netherlands; ‡ PLASMANT, Department of Chemistry, University of Antwerp, BE-2610Antwerp, Belgium; ¶ ALTEN Nederland, Technology, Fascinatio Boulevard 582, 2909 VACapelle a/d IJssel, The Netherlands

## Abstract

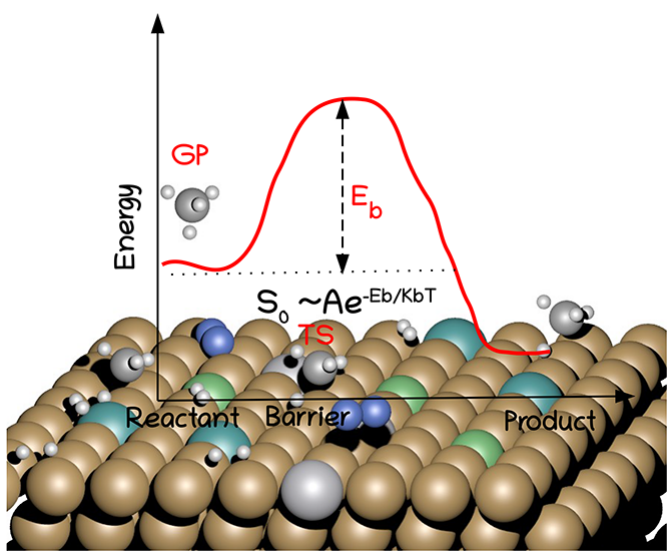

Accurate barriers for rate controlling elementary reactions on metal surfaces are key to understanding, controlling, and predicting the rate of heterogeneously catalyzed processes. While barrier heights for gas phase reactions have been extensively benchmarked, dissociative chemisorption barriers for the reactions of molecules on metal surfaces have received much less attention. The first database called SBH10 and containing 10 entries was recently constructed based on the specific reaction parameter approach to density functional theory (SRP-DFT) and experimental results. We have now constructed a new and improved database (SBH17) containing 17 entries based on SRP-DFT and experiments. For this new SBH17 benchmark study, we have tested three algorithms (high, medium, and light) for calculating barrier heights for dissociative chemisorption on metals, which we have named for the amount of computational effort involved in their use. We test the performance of 14 density functionals at the GGA, GGA+vdW-DF, and meta-GGA rungs. Our results show that, in contrast with the previous SBH10 study where the BEEF-vdW-DF2 functional seemed to be most accurate, the workhorse functional PBE and the MS2 density functional are the most accurate of the GGA and meta-GGA functionals tested. Of the GGA+vdW functionals tested, the SRP32-vdW-DF1 functional is the most accurate. Additionally, we found that the medium algorithm is accurate enough for assessing the performance of the density functionals tested, while it avoids geometry optimizations of minimum barrier geometries for each density functional tested. The medium algorithm does require metal lattice constants and interlayer distances that are optimized separately for each functional. While these are avoided in the light algorithm, this algorithm is found not to give a reliable description of functional performance. The combination of relative ease of use and demonstrated reliability of the medium algorithm will likely pave the way for incorporation of the SBH17 database in larger databases used for testing new density functionals and electronic structure methods.

## I Introduction

Heterogeneous catalyzed processes are of large importance to the chemical industry,^1^ with well-known examples of such processes being ammonia synthesis^2^ and steam reforming.^3^ In heterogeneously catalyzed processes on metal surfaces, the steps with a high degree of rate control often involve the dissociative chemisorption (DC, the process whereby the interaction of a molecule with a surface leads to the breaking of a bond in the molecule and the formation of two new bonds of the molecular fragments to the surface) of a molecule on the surface.^4,5^ Understanding how heterogeneous catalysis works is of huge importance. Our ability to understand the different mechanisms underlying the DC on metal surfaces could benefit significantly from the availability of an accurate database for barrier heights of elementary molecule-metal surface reactions. Just like chemisorption energies of (intermediate) reactants and products, accurate barriers for rate controlling elementary reactions are key to understanding, controlling, and predicting the rate of overall heterogeneously catalyzed processes.^6−9^

Ideally, accurate barrier heights could be extracted directly from detailed systematic experiments. However, it is not possible to measure barrier heights for DC directly. An observable that can be measured experimentally and that is strongly related to the barrier height for the DC is the sticking probability (S_0_).^10^ The best way to access barrier heights using theory is through a theoretical approach in which potential energy surfaces (PESs) are computed and used in dynamics calculations to evaluate S_0_ as a function of average incidence energy.^10^ Comparison with experimental S_0_^10−14^ will then allow one to evaluate the accuracy of the electronic structure method used to compute the PES for the calculated barrier height.^10^ Only when experimental data are reproduced within chemical accuracy (i.e., with errors smaller than 1 kcal/mol^11,12^) to a sufficiently large extent, a claim can be made that the computed barrier height is of high accuracy.

For adsorption bond energies to transition metal surfaces, a database containing 39 entries for use with DFT benchmarking studies has recently been constructed.^15^ This database, subsets of it,^16,17^ and a slightly extended version^18^ of it have been used in several benchmark DFT studies,^15−21^ and a considerably extended database containing 81 entries also exists.^22^ Barrier heights for gas phase reaction have been extensively benchmarked.^23−26^ However, barriers for the DC on metal surfaces have been mapped out to a much smaller extent^10^ and have been little used for benchmark calculations. For many gas phase reactions, it has been possible to use the very accurate CCSD(T)^27^ electronic structure method to compute reference values. On the other hand, for molecule-metal surface reactions, until very recently only semilocal density functional theory (DFT)^28^ could be used, which is much less accurate. As a result, it is not yet known how large the errors in barriers for molecule-metal surface reactions are when using standard exchange-correlation (XC) functionals. For reactions occurring in the gas phase, it is well-known that the density functionals (DFs) at the second rung on Jacob’s ladder^28,29^ (GGA level^30,31^) underestimate barrier heights as a consequence of self-interaction errors.^24,32^ An idea of the performance of semilocal functionals on gas phase reaction barriers can be obtained from their performance on the BH206 database,^24^ tests showing that application of the best performing MN12-L^33^ and N12^34^ nonseparable meta-GGA and GGA DFs resulted in root-mean-square deviations of 4.3 and 7.1 kcal/mol, respectively. To overcome this potential problem of the XC functional for molecule-metal surface reactions, the SRP-DFT method^35,36^ (which uses weighted averages of two XC functionals) has been adopted for such reactions.^11^ This semiempirical (SE) method has allowed prediction of barrier heights to within chemical accuracy (1 kcal/mol) for specific systems.^10^

Some theoretical studies have been carried out recently in attempts to build databases of barrier heights for molecule-metal surface reactions. The first database (CatApp^37,38^) was built based on DFT calculations using only one functional (RPBE^39^). More recently, a first attempt was made to construct a database of molecule-metal surface reaction barriers for benchmarking purposes.^40^ This database, called SHB10, contained 6 entries based on SRP-DFT and 4 entries based on more ad-hoc SE procedures. The SBH10 database was used^40^ to test the performance of one DF consisting of GGA exchange and nonlocal correlation (BEEF-vdW-DF2^16^), one meta-GGA (MS2^41^), and one screened hybrid DF (HSE06^42^). A surprising conclusion was that BEEF-vdW-DF2 performed the best.

With more than 30 000 papers published annually,^43^ DFT arguably is the most important electronic structure method for dealing with complex systems. It is therefore important to develop a large enough database that allows testing the method on barrier heights for molecule-metal surface reactions. As discussed below, accurate SRP-DFT barriers for the DC are now available for 14 molecule-metal surface reactions, allowing the former database to be extended with 7 systems if additional results from three more ad-hoc procedures are included as before. In the present paper, we therefore develop a new and larger database for benchmarking (SBH17), which contains benchmark results for 17 systems. We now also test a much larger number of DFs on this larger database, i.e., 3 GGA-type DFs, 4 meta-GGA DFs, and 7 DFs containing GGA exchange and nonlocal correlation. In performing these tests, we also take an improved approach over that taken in the previous paper,^40^ in which the metal surface was allowed to relax in response to the incoming molecule while computing the barrier height. This approach is flawed in that the metal surface atoms have too little time to respond to the motion of the incoming molecule in the hypersonic molecular beam experiments employed to performing sticking experiments, which are used in the SE procedure to construct SRP DFs.^10^

In performing the tests of the 14 DFs to be discussed below, three different algorithms will be used to compute barrier heights, which differ in the computational effort that may be required to compute metal lattice constants and metal slabs that have interlayer distances simulating metal surfaces that have been relaxed to describe their interaction with the vacuum, and to locate the transition state geometry for a specific functional. These three algorithms will be compared among each other for their performance. A new database for molecule-metal surface reaction barriers is of course more likely to be used if it meets the following two demands, which may conflict with one another. When used in testing new functionals or electronic structure methods in general, the algorithm should be as easy and straightforward to use and require as little computational effort, as possible. At the same time, the algorithm should also still yield reliable results regarding how functionals or new methods perform, because otherwise it would not be useful.

The outline of our paper is as follows: In Section II, the methods used are explained, beginning with the DFs tested in Section II.1. The description of the SE procedures used to obtain reference values of barrier heights and the motivation of the use of SRP-DFT are presented in Section II.2, the choice of the reference values is clearly explained in Section II.3, and the details of the algorithms used are described in Section II.4. The results are presented in Section III, beginning with the structure of the metals in Section III.1, while Section III.2 presents the DC barriers. The discussion is provided in Section IV. The description of the metals with the DFs tested is discussed in Section IV.1. The description of the barrier heights to the DC is discussed in Section IV.2. In this Section, the performance of the algorithms is discussed in Section IV.2.1. Subsequently, the performance of the DFs using the medium algorithm for SBH17 is discussed in Section IV.2.2. The dependence of the performance for the barrier heights on the type of system is discussed in Section IV.2.3. The comparison with results for the previous SBH10 database is provided in Section IV.2.4. Section IV.3 provides a comparison of how the DFs tested perform on the SBH17 database for the DC barriers (kinetics), to how they perform for molecular chemisorption (thermochemistry), and to how they perform for gas phase kinetics and thermochemistry. A discussion on future improvements is given in Section IV.4. Finally, the conclusions and outlook are given in Section V.

## II Methods

### II.1 Density Functionals Tested

The DFs that we have tested on reaction barriers for the DC on metal surfaces, as presented in our new database discussed below, are listed in Table 1. Of these XC DFs, three fall in the GGA^28^ category, seven consist of GGA exchange^28^ and vdW-DF1^44^ or vdW-DF2^45^ Rutgers-Chalmers type nonlocal correlation, and four fall within the meta-GGA^28^ category. Here, we will only briefly describe the DFs tested; for details, we refer to the original papers.

**Table 1:** XC Functionals Tested in This Work and How Their Exchange and Correlation Parts Are Chosen^a^

	name	type	exchange	correlation
1	PBE	GGA	PBE^46^	PBE^46^
2	RPBE	GGA	RPBE^39^	PBE^46^
3	SRP50	GGA	0.50RPBE(ref ^39^)+0.50PBE(ref ^46^)	PBE^46^
4	vdW-DF1	GGA+vdW	revPBE^191^	vdW-DF1^44^
5	vdW-DF2	GGA+vdW	rPW86^50^	vdW-DF2^45^
6	PBE-vdW-DF2	GGA+vdW	PBE^46^	vdW-DF2^45^
7	SRP32-vdW-DF1	GGA+vdW	0.32RPBE(ref ^39^)+0.68PBE(ref ^46^)	vdW-DF1^44^
8	PBEα57-vdW-DF2	GGA+vdW	PBEα = 0.57^192^	vdW-DF2^45^
9	BEEF-vdW-DF2	GGA+vdW	BEEF^16^	BvdW-DF2^16,45^
10	optPBE-vdW-DF1	GGA-vdW	optPBE^99^	vdW-DF1^44^
11	revTPSS	meta-GGA	revTPSS^53^	revTPSS^53^
12	SCAN	meta-GGA	SCAN^54^	SCAN^54^
13	MS-B86bl^55^	meta-GGA	MS-B86bl	revTPSS^53^
14	MS2	meta-GGA	MS2^41^	MS2^41^

a The type ‘GGA-vdW’ means that GGA exchange is combined with vdW-DF1^44^ or vdW-DF2^45^ correlation.

In the GGA, which is at the second rung of “Jacob’s ladder”,^28,29^ use is made of the density and its gradient. As discussed by Perdew,^28^ at the GGA level, a constraint based DF can be made to satisfy a subset of constraints but not all known constraints. For applications to surface reaction dynamics, to some extent, the constraint based PBE and RPBE DFs selected here may be considered to be “at extremes”, with PBE^46^ often underpredicting and RPBE^39^ often overpredicting reaction barrier heights according to conventional wisdom.^10^ The PBE DF^46^ is often considered to be a “workhorse” GGA DF, in a sense that it describes a range of properties of molecules and materials with a fair accuracy. The PBE DF was designed to replace the PW91^47^ DF, yielding similar results while employing a mathematical framework superior to that of PW91. The RPBE DF is mainly used for molecule-metal surface interactions and was introduced to correct for the overbinding observed for adsorption of small atoms and molecules to metal surfaces^39^ as obtained with the PBE DF. In addition to RPBE and PBE, we also test a 50/50% mixture of these DFs, which is called SRP50 here. The choice of this DF stems from the similar 48/52% RPBE/PBE mixture providing a chemically accurate description of the well-studied H_2_ + Cu(111) system (see also below). We only test 3 GGA DFs here because they suffer from a fundamental drawback: optimizing GGA DFs for their performance of adsorption energies of molecules to metal surfaces goes at the cost of an accurate description of the metal surface itself.^48,49^ It has been argued that this problem can be solved with GGA DFs of which the XC DF is nonseparable in an exchange and a correlation part,^34^ but we do not test such DFs here.

Like the meta-GGA DFs discussed below, GGA DFs are not capable of a reasonably accurate description of the van der Waals interaction. For this reason, and because we are dealing with metals, we have tested seven DFs consisting of GGA exchange and nonlocal correlation functionals, for which we use either one of two Rutgers-Chalmers correlation functionals, which we call vdW-DF1^44^ and vdW-DF2,^45^ respectively. These van der Waals DFs were originally designed to be a part of a nonempirical XC DF where the exchange DF was somehow matched to the specific correlation DF,^44,45^ and these nonempirical XC DFs, which are both tested here, are simply called vdW-DF1 and vdW-DF2 here. The vdW-DF2 correlation DF has also been incorporated in the so-called BEEF-vdW DF (here called BEEF-vdW-DF2) also tested here, which was semiempirically fitted to adsorption energies on transition metal surfaces, gas phase reaction barriers, and other properties.^16^ The optPBE-vdW-DF1 functional is an example of a DF in which the vdW-DF1 correlation functional has been combined with a semiempirically adjusted exchange DF, in this case to obtain good interactions of weakly interacting dimers.^50^ Finally, the PBE-vdW-DF2, SRP32-vdW-DF1, and PBEα57-vdW-DF2 are combinations of GGA exchange DFs and vdW-DF1 or vdW-DF2 correlation DFs designed to describe particular DC systems with chemical accuracy, i.e., H_2_ + Ru(0001),^51^ CH_4_ + Ni(111),^13^ and H_2_ + Pt(111),^52^ respectively. These DFs are more fully described in Table 1. We note that for all of the DFs incorporating vdW-DF1 or vdW-DF2 discussed here except BEEF-vdW-DF2, the full correlation functional can be written as the sum of correlation from the local density approximation (LDA) and a nonlocal functional, which is the nonlocal part of the vdW-DF1^44^ or vdW-DF2.^45^ For BEEF-vdW-DF2, the full correlation functional is written as a weighted average of the LDA and the semilocal PBE correlation functional (with the sum of the weights equal to 1)^16^ plus the nonlocal part of vdW-DF2.^45^ To emphasize this difference, the correlation DF of BEEF-vdW-DF2 is represented by the acronym BvdW-DF2 in Table 1.

In the meta-GGA, which is at the third rung of “Jacob’s ladder”,^28,29^ additional use is made of the kinetic energy density, which is equivalent to the Laplacian of the electron density. Of these, the revTPSS DF^53^ was designed to be the workhorse counterpart of the GGA PBE DF. The SCAN DF was designed to enforce all known physical constraints on the DF^54^ (this can be done at the meta-GGA level). The MS2 functional has two semiempirically fitted parameters in it and was designed with the specific aim of accurately describing both metals and molecules.^41^ Finally, the MS-B86bl DF has been shown to accurately describe the earlier mentioned H_2_ + Cu(111) system, and its design^55^ should ensure reasonable accuracy for any system in which H_2_ interacts with a metal surface. Again, details on the composition of these XC DFs may be found in Table 1.

In hybrid DFs, which are at the fourth rung of Jacob’s ladder,^28,29^ a fraction of the semilocal exchange in the exchange part of the XC functional is replaced by exact exchange. Screened exact exchange DFs (in which the exact exchange component is switched off at large electron–electron distances) have been used in a few instances in studies of a specific DC system (see e.g. ref (56)). However, their use is computationally expensive, and a screened hybrid DF was only used to study 4 of the 10 systems addressed in the SBH10 paper. For this reason, and because their use will be more appropriate once systems are addressed for which electron transfer from the surface to the molecule is likely,^56^ we will not test such functionals here.

In rung 5 functionals,^28,29^ virtual orbitals are added in addition to exact exchange. The random phase approximation (RPA)^57−60^ is a well-known example of such functionals. The RPA has been used in one specific study of reaction barriers in a DC molecule-metal surface system that we know of^61^ and in a limited number of benchmark studies of molecular adsorption on metal surfaces.^17,62^ However, its use is even more computationally expensive than that of hybrid functionals. For this reason, we have not tested the RPA, nor have we tested any other rung 5 DFs.

### II.2 Semiempirical Approaches to Obtaining Reference Values of Barrier Heights

In determining reference values for barrier heights of gas phase reactions for use in databases, theorists have often benefited from the availability of electronic structure methods and associated algorithms delivering reaction barrier heights with chemical accuracy. For instance, barriers for the NHBTH38 database (a database for 38 non-hydrogen atom transfer reactions) were obtained with an algorithm in which results obtained with the highly accurate CCSD(T)^27^ method were extrapolated to the basis set limit.^63^ In the construction of the HBTH38 database, theorists likewise relied on barrier heights obtained from high level ab initio electronic structure methods, although in this case, the *ab initio* results were also compared to experiment to extract best guesses (i.e., reference values) of barrier heights.^64,65^

As already noted in the Introduction, the situation is quite different in the field of reaction dynamics on metal surfaces. In this field, semilocal density functionals are routinely applied to the DC reactions occurring on metal surfaces. However, the results are semiquantitative at best, as one might expect from the performance of these functionals on gas phase reactions.^10,51,66,67^ In attempts to do better, the first-principles diffusion Monte Carlo (DMC) method has been used to compute barrier heights for e.g. N_2_ + Cu(111)^68^ and for H_2_+ Mg(0001),^69^ Cu(111),^70^ and Al(110).^71^ The results for H_2_ + Cu(111)^70^ suggested that DMC can deliver near chemical accuracy for barrier heights for the DC on transition metal surfaces (accuracy better than 2 kcal/mol), in line with results for the HBTH38 and NHBTH38 gas phase reaction barrier databases.^10,72,73^ However, chemical accuracy was not yet achieved for this benchmark reaction, and DMC calculations are computationally expensive. Embedded correlated wave function (ECW) calculations based on multireference perturbation theory embedded in DFT provided near chemical accuracy for a DC reaction on a simple metal surface (O_2_ + Al(111)^74^). However, the computational expense of such calculations is presently too high for molecules interacting with transition metals (TMs), as calculations^75^ on H_2_ + Cu(111) suggest. Zhao et al. made a positive assessment of their ECW method on the basis of the comparison of the emb-CASPT2 barrier height for the DC of H_2_ on Cu(111) (0.15 eV)^75^ with an “experimental” value^76^ from the literature (0.05 eV). However, this value was extracted through an invalid extrapolation procedure (over temperature, to 0 K, see Figure 15 of ref (76)) in an analysis that was at best approximate for higher temperatures and originally meant to make contact with kinetics experiments.^76^ The best value of the H_2_ + Cu(111) barrier height is however 0.63 eV^11^ and not 0.05 eV.

As argued in great detail in ref (10) (to which we refer for these details), accurate reaction barriers heights for DC reactions on metals are therefore best determined through an SE approach. This approach is best based on supersonic molecular beam experiments that probe the reactivity on the ideal surface, whereas rate measurements usually probe the reaction at (often unknown) defects,^77,78^ making the latter experiments less useful for benchmarking purposes.^10,79^ The basic idea of the SE SRP-DFT approach used to extract reference barrier heights is to adjust a DF until appropriate dynamics calculations on the basis of that DF yield agreement with measured DC probabilities. The correctness of this procedure can be argued^10^ on the basis of the so-called hole model,^80^ which essentially states that computed reaction probabilities will be correct if the potential energy surface (and the minimum barrier height extracted from it) is correct. We deem the approach to deliver chemical accuracy because numerous instances have now shown that with appropriate dynamics methods and models measured DC probability curves can be reproduced to within energy shifts less than 1 kcal/mol on the basis of appropriately constructed functionals. Essentially the spirit of the method is not so different from the approach taken to originally construct the HTBH38 gas phase reaction barrier database, which also combined theoretical and experimental information.^64,65^ We also recall that in any case a reaction barrier height is not an observable. The procedure to validate a computed barrier height through comparison with an experiment must necessarily take recourse to the use of a measured observable that is as closely related to the barrier height as possible.

The SE SRP-DFT approach discussed above is used for most reactions in the SBH17 database (i.e., for 14 out of 17 cases). With this approach, an appropriate dynamical method and model was used to model supersonic molecular beam experiments in all but one case (CH_4_ + Ni(211), see below).^10^ This means, for instance, that all (or all relevant) molecular degrees of freedom were usually modeled in dynamics calculations. We will discuss the SRP-DFT electronic structure method used for these cases in Section II.2.a below. In the earlier SBH10 database,^40^ four systems were introduced for which reference values were derived using experiments and their analysis by a more primitive SE approach. These analyses were carried out before 2009, when SRP-DFT became available.^11^ Reference values for three of these systems in our present SBH17 database were inherited from the earlier SBH10 database, which we will briefly discuss in Section II.2.b below. (For one of the four systems (CH_4_ + Ni(211), called the ‘CH_4_/Ni(111) step’ in ref (40), accurate results are now available, and we have moved this system to the SRP-DFT part of the database.) As will also be discussed below, it would be good if the reference values for these three systems be replaced in the future by more accurate values from for instance SRP-DFT. For each system in the SBH17 database, Section II.3 describes what the specific reference value used for the system is and how it was derived.

#### II.2.a The Specific Reaction Parameter Approach to Density Functional Theory (SRP-DFT)

The SRP-DFT method as introduced is an SE method and was originally applied to reactions in the gas and condensed phases by Truhlar and co-workers.^35,36^ SRP-DFT was first applied to the DC on a metal surface by Díaz et al.^11^ They used an implementation in which the SRP-DF is a weighted average of two GGA DFs according to a mixing parameter ***x***. Changing the mixing parameter “tunes” the functional to reproduce S_0_, which is strongly correlated with the minimum barrier height. In the most straighforward approach, a GGA XC DF that underestimates and one that overestimates the barrier height is used:
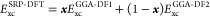
1Standard GGA DFs often used for mixing in applications to molecule-metal surface reactions are the RPBE^39^ and PBE^46^ functionals discussed in section II.1. For weakly activated H_2_-metal and for CH_4_-metal systems, the correlation part of the SRP-DF is best substituted by the van der Waals nonlocal correlation functional of Dion et al.^44^ (vdW-DF1) or of Lee et al.^45^ (vdW-DF2), changing eq 1 to become
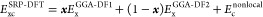
2In eq 2, the mixing parameter only tunes the exchange part of the XC DF in the SRP-DF. Instead of a weighted average of two XC or exchange DFs, one can also use an inherently tunable DF, such as the PBEα DF or the exchange part of it, in which α can be adjusted. Using nonlocal correlation, the equation for the SRP-DF then becomes
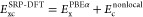
3As originally defined, a DF is only considered to be an SRP-DF if after fitting ***x*** it not only reproduces a particular sticking experiment with chemical accuracy but also reproduces another experiment on the same system with comparable accuracy.^11^ In contrast, if a parametrized DF only reproduces the sticking experiment it was fitted to, it was originally called a candidate SRP-DF.^10^ Here, we drop this distinction and refer to both categories of DFs as SRP-DFs. Additionally, the SRP-DF can be considered to be transferable if it can reproduce experimental results for a system it was not fitted to.^81^ For example, in some cases, the SRP-DF fitted to reproduce molecular beam dissociation chemisorption experiments for H_2_ and D_2_ was shown to be transferable among systems in which H_2_ interacts with different crystal faces of the same metal.^82−85^ One downside of the SE SRP-DFT approach to the DC of the molecules on the metal surfaces used so far, in which semilocal exchange DFs are used, is that successful applications of this approach have only been demonstrated to systems for which the difference of the metal work function (W) and the molecule’s electron affinity (EA) is larger than 7 eV. The SRP-DF approach has allowed the construction of chemically accurate barriers for 14 systems^10,84^ with (W-EA) > 7 eV, as shown in Table 2 and now discussed further below.

**Table 2:** Summary of the SBH17 Database^a^

N_*s*_	system	functional	site	*r*_*b*_	*Z*_*b*_	θ	φ/β	E_*b*_
1	H_2_ + Cu(111)^11^	SRP43	brg	1.03	1.16	90	90	0.628
2	H_2_ + Cu(100)^85^	SRP43	brg	1.23	1.0054	90	90	0.740
3	H_2_ + Cu(110)^88^	optPBE-vdW-DF1	short-brg	1.20	0.89	64	90	0.789
4	H_2_ + Pt(111)^52^	PBEα57-vdW-DF2	top (early)	0.769	2.202	90	0	–0.008*
5	H_2_ + Pt(211)^82^	PBEα57-vdW-DF2	top	0.75	2.79	90	90	–0.083*
6	H_2_ + Ru(0001)^51^	PBE-vdW-DF2	top (early)	0.751	2.605	90	0	0.004*
7	H_2_ + Ni(111)^110^	PBE-vdW-DF2	top (early)	0.763	2.083	90	0	0.024*
8	H_2_ + Ag(111)^84^	MS-PBEl-rVV10	brg	1.224	1.157	90	0	1.082*
9	N_2_ + Ru(0001)^116^	RPBE	brg	1.741	1.318	84	30	1.840
10	N_2_ + Ru(101̅0)^77,120^	experiment						0.40
11	CH_4_ + Ni(111)^13^	SRP32-vdW-DF1	top	1.606	2.176	135.7	164.7	1.015 (1.055)
12	CH_4_ + Ni(100)^122^	experiment						0.76
13	CH_4_ + Ni(211)^123^	SRP32-vdW-DF1	top	1.632	2.033	126.0		0.699
14	CH_4_ + Pt(111)^81^	SRP32-vdW-DF1	top	1.56	2.28	133.4	168.3	0.815 (0.856)
15	CH_4_ + Pt(211)^14^	SRP32-vdW-DF1	top	1.53	2.27	133	168	0.559 (0.581)
16	CH_4_ + Ir(111)^121^	SRP32-vdW-DF1	top					0.836
17	CH_4_ + Ru(0001)^135^	experiment						0.80

a Barrier heights (in eV) and the most important co-ordinates defining the barrier geometry are presented. The “site” defines the projection of the molecule’s center-of-mass position on the surface, *r*_*b*_ (in Å) is the length of the dissociating bond, and *Z*_*b*_ (in Å) is the distance of the molecule’s center-of-mass to the surface. The molecule’s orientation is defined by the polar angles of orientation (θ) of the diatomic molecule or partly defined by the (θ,φ) pair of angles giving the polar angle of the breaking CH-bond and the umbrella axis of the remaining methyl fragment makes with the surface normal, respectively. Barrier heights obtained from PESs used in the dynamics are marked with an asterisk (*). For some CH_4_ + metal systems, the barrier height is also given without residual correction (in brackets, see the text).

The supersonic beam experiments referred to above need to be modeled with an appropriate dynamical method (e.g., quasi-classical or quantum dynamics) and dynamical model. Here, the latter refers to whether or not all molecular degrees of freedom, the motion of the surface atoms, and electron–hole pair (ehp) excitation are considered.^10^ Because dynamics rather than transition state theory is used, and because the surface atoms usually do not have time to respond to the incoming molecule, it makes the most sense to tabulate “classical reaction barrier heights”. By this we mean barrier heights arising directly from electronic structure calculations without corrections for zero-point energies (zpes) and entropy effects, for the molecule interacting with the “ideal” surface, i.e., with the surface atoms sitting in their equilibrium lattice positions for a classical 0 K surface. The SRP-DFT barriers reported below all are classical barrier heights computed with an SRP-DF or with a PES based on SRP-DFT calculations.

#### II.2.b Ad Hoc Semiempirical Approaches

As noted above, for three systems (CH_4_ + Ni(100), CH_4_ + Ru(0001), and N_2_ + Ru(101̅0)), reference values were taken from the paper on the SBH10 database, and these were extracted using a more primitive SE approach than used in SRP-DFT. As will be detailed below in Section II.3, reduced dimensionality modeling of supersonic molecular beam sticking experiments was used to derive a minimum barrier height for CH_4_ + Ni(100). Thermal S_0_ measured for N_2_ dissociating on Ru(0001) was fitted to an Arrhenius type equation to derive an activation energy for the DC at defects, which were considered to be the steps occurring in Ru(101̅0). Finally, an activation energy for CH_4_ dissociation on Ru(0001) was derived from associative desorption experiments as described below, invoking detailed balance. Even though activation energies were derived for N_2_ and CH_4_ dissociation on Ru(101̅0) and Ru(0001), respectively, we felt that the approaches used were too crude to attempt extracting classical minimum barrier heights for these systems by subtracting zpe corrections using known approximate values.^40^ Instead, we simply use the semiempirically extracted activation energies as reference values for the minimum barrier heights for these two systems.

### II.3 The SBH17 Database

The systems that constitute our SBH17 benchmark database of barrier heights for the DC on transition metal surfaces are listed in Table 2. This table contains reference barrier heights and data concerning the barrier geometries for 17 systems. The bulk of the data comes from SRP-DFT, such that 14 entries in Table 2 may also be viewed as constituting a database that can be named SBH14/SRP. Three entries in Table 2 come from more ad-hoc SE approaches, as also discussed in the original SBH10 paper.^40^ In this section, we justify our choice of the reference values of the barrier height and our reference geometries, which is important to do especially in cases where conflicting data exists. Note that barrier heights obtained from SRP-DFT are given in eV using 3 significant digits behind the decimal place (i.e., expressed in meV), even though the accuracy claimed for these numbers is only one kcal/mol ≈ 43 meV, with the claim based on the energy shift between the sticking probabilities that were measured and computed on the basis of the SRP DF yielding the minimum barrier height being smaller than 1 kcal/mol,^10,11^ as more fully discussed in Section II.2. In doing this, we follow a rather common practice in computational chemistry, as this will allow other researchers to check whether they can reproduce our numbers. The barrier heights extracted using more ad-hoc approaches (Section II.2.a) have been stated with the amount of significant digits used originally by the scientists providing these benchmark results, and the errors in these reference values may well be larger than 1 kcal/mol. Finally, we note that the average value of the absolute barrier heights of SBH17 is 14.8 kcal/mol.

#### II.3.a Dissociative Chemisorption of H_2_ on Transition Metals

#### H_2_ on Cu(111), Cu(100), and Cu(110)

The DC of H_2_ on copper surfaces perhaps represents the most widely studied class of DC systems by both theory^11,84,86−89^ and experiment.^76,89−93^ Being activated systems, in the absence of strong effects of ehp excitation and energy transfer involving phonons^94^ on reactive scattering, they represent perfects systems for benchmarking electronic structure methods for their capability to accurately predict barriers.

#### H_2_ + Cu(111)

The first system for which an SRP-DF was derived for the DC on a metal surface was H_2_ on Cu(111),^11^ and the first SRP-DF for this system (SRP43) was a weighted average of the PW91^47^ (57%) and the RPBE^39^ (43%) DF. With the PES developed with this SRP-DF and using the BOSS model quasi-classical trajectory and time-dependent wave packet calculations reproduced measured molecular beam S_0_ for H_2_ and D_2_, initial-state selected reaction probabilities for H_2_,^76,91^ and data for rotationally inelastic scattering^95^ to within chemical accuracy. Density functional molecular dynamics (DFMD) calculations with the subsequently developed SRP48-DF^96^ (48% RPBE^39^ and 52% PBE^46^) also accurately reproduced measured^97^ rotational quadrupole alignment parameters A_0_^(2)^(J) and enabled a chemically accurate description of initial-state selected reaction probabilities of D_2_ on Cu(111), after an appropriate reanalysis of the experimental data.^86^ Recent studies^88,98^ using the optPBE-vdW-DF1 exchange combined with nonlocal vdW-DF1 correlation (reparameterized PBE for vdW-DF1)^99^ also provided a chemically accurate description of S_0_ for H_2_ and D_2_ on Cu(111). Additionally, three different combinations of GGA exchange DFs combined with nonlocal vdW-DF2 correlation^98^ allowed chemically accurate descriptions of the reaction of H_2_ and D_2_ on Cu(111), and the same was true for three newly developed meta-GGA DFs.^55^ The barriers reported for the vdW-DF1 and vdW-DF2 combinations and the new meta-GGA DFs were somewhat different from the one obtained with the original SRP43 DF (The SRP48 DF was designed to reproduce the SRP43 energy at the SRP43 barrier geometry^96^.). As a reference for our benchmark database, the SRP43 barrier height (0.636 eV)^11^ will be used. While calculations with some of the other mentioned DFs in cases described the sticking experiments more accurately than SRP48^96^ or SRP43,^11^ only calculations with the latter 2 DFs reproduced initial-state selected reaction probabilities extracted from associative desorption experiments with chemical accuracy, suggesting that these two DFs should be the DFs best describing H_2_+Cu(111).^98^

#### H_2_ + Cu(100)

H_2_ on Cu(100) is the second system for which an SRP-DF was demonstrated.^85^ The SRP-DF(SRP43^11^) originally developed for H_2_ on Cu(111) could also be used to reproduce the measured S_0_^90^ for H_2_ on Cu(100) within the BOSS model.^85^ This also represents an example of the transferability that SRP-DFs may exhibit for chemically closely related systems,^10^ in this case systems in which the same molecule interacts with different low index faces of the same metal. As a reference value for our database, we use the value of the barrier height reported for SRP43^85^ (0.74 eV).

#### H_2_ + Cu(110)

In a recent study, a new SRP-DF was demonstrated for H_2_ + Cu(110).^88^ The optPBE-vdW-DF1 functional was used to develop PESs based on embedded atom neural network (EANN) fits for H_2_ on Cu(111), Cu(100), and Cu(110) by Jiang and co-workers.^88^ Dynamics calculations employing the resulting PES for H_2_ + Cu(110) yield a chemically accurate description of molecular beam sticking experiments on H_2_ + Cu(110).^100^ The optPBE-vdW-DF1 functional had previously^101^ been shown to yield a chemically accurate description of molecular beam sticking experiments of D_2_ on Cu(111).^102^ Jiang and co-workers also demonstrated chemically accurate descriptions of sticking experiments on H_2_ + Cu(111) and Cu(100). This therefore represents another example of transferability of SRP-DFs among chemically related systems,^10^ where one DF (optPBE-vdW-DF1) can be used for model sticking of one and the same molecule on several low index faces of the same metal. The barrier height reported by Jiang and co-workers for their PES (0.789 eV)^88^ will be used as the reference value for our database.

#### H_2_ on Pt(111) and Pt(211)

#### H_2_ + Pt(111)

H_2_ on Pt(111) is considered as a weakly activated system because of its low minimum barrier height. Three DFs have been found that describe the sticking of D_2_ on Pt(111) with chemical accuracy.^52,98^ The SRP-DF first developed for D_2_ + Pt(111) was the PBEα57-vdW-DF2 functional (see Section II.2 and Tables 1 and 2). With this DF measured^103^ S_0_ for both normal and off-normal incidence of D_2_ was reproduced with chemical accuracy.^52^ The SRP48^96^ and a DF consisting of 68% B86r exchange^104^ and 32% RPBE exchange^39^ combined with vdW-DF2 correlation^45^ (SRPB86r68-vdW-DF2) also both reproduced the measured^103^ S_0_ for normal incidence with overall chemical accuracy.^98^ However, the PBEα57-vdW-DF2 resulted in the most accurate results near the reaction threshold,^52^ suggesting that this DF yields the barrier height with the highest accuracy.^98^ Furthermore, recent work has shown that this DF can reproduce experiments of D_2_ on chemically related curved Pt crystals with (111) terraces and (100) steps with chemical accuracy.^105^ The barrier height reported for PBEα57-vdW-DF2 was −0.008 eV. We retain this value as the reference value (see Table 2), even though it was set to 0.0 eV in the previous SBH10 database.^40^

#### H_2_ + Pt(211)

The PBEα57-vdW-DF2 functional developed for H_2_ on Pt(111) was also employed to test transferability to H_2_ on Pt(211).^82^ This SRP-DF also yields^82^ a chemically accurate description of experiments on the DC of H_2_ and D_2_ on the stepped Pt(211) surface.^106^ The lowest barrier height found in reduced dimensionality (by finding saddle points in the reduced 2D spaces formed by the elbow plots in Figure 4 of ref (82)) was −0.083 eV, and this is the value that we use, along with the “top1 (φ = 90°)” geometry defined in ref (82).

#### H_2_ + Ru(0001)

Like H_2_ + Pt(111), H_2_ on Ru is a weakly activated system. For this system, two DFs were found^51^ to reproduce measured^107^ S_0_ for H_2_ + Ru(0001) with chemical accuracy. These DFs were the PBE-vdW-DF2 functional (see Tables 1 and 2) and the functional containing 50% PBE^46^ and 50% RPBE^39^ exchange combined with vdW-DF1 correlation^44^ (SRP50-vdW-DF1). The barrier height reported for both DFs was 0.004 eV. This is the value we use in our database, even though it was set to 0.0 in the previous SBH10 database.^40^

#### H_2_ + Ni(111)

The DC of H_2_ on Ni(111) is also weakly activated. Similar to the case of H_2_ on Ru(0001), agreement with existing sticking experiments^108,109^ was achieved^110^ to within chemical accuracy with dynamics calculations based on the PBE-vdW-DF2 functional (see Tables 1 and 2). The PBE-vdW-DF2 calculations for H_2_ + Ni(111) were done with the spin-corrected vdW-DF2 functional^111^ (spin-vdW-DF2) to take into account the magnetic character of the Ni(111) surface, whereas for all other considered systems, the original nonspin corrected vdW-DF1 and vdW-DF2 functionals were used. The barrier height reported is that of the early top site barrier (as also used for H_2_ + Pt(111) and Ru(0001)), which is 0.024 eV.^110^ In all VASP calculations we perform here, we employ the nonspin corrected vdW-DF1 and vdW-DF2 functionals; however, we note that earlier calculations suggested little influence of the spin-correction on the barrier height computed for CH_4_ + Ni(111) with a functional featuring vdW-DF1 correlation.^13^ The barrier height we use as the reference value (obtained with PBE-spin-vdW-DF2) in our database is 0.024 eV.

#### H_2_ + Ag(111)

H_2_ + Ag(111) is a highly activated system, for which molecular beam sticking experiments were performed by Hodgson and co-workers.^112^ Recently it was shown^84^ that the measurements^112,113^ can be reproduced with chemical accuracy using recently developed made-simple meta-GGA exchange DFs^55^ combined with rVV10 nonlocal correlation.^114^ Here, we use the barrier height obtained with the functional yielding the best agreement with experiment (MS-PBEl-rVV10)^84^ as the reference value for our database (1.082 eV).

#### II.3.b N_2_ Dissociation on Ru Surfaces

#### N_2_ + Ru(0001)

Ru is well-known as a catalyst for the Haber-Bosch process used to make ammonia, which is a raw material for artificial fertilizer.^115^ As noted in the original SBH10 paper,^40^ for N_2_ + Ru(0001), barrier heights are available from both SRP-DFT^116,117^ and from a direct estimate based on experimental results.^118^ The directly estimated barrier height based on a laser-assisted associative desorption experiment^118^ was 1.8 eV, whereas the calculations based on the RPBE DF that were found to give a chemically accurate description^116,117^ of the best experimentally measured S_0_^119^ gave a barrier height of 1.84 eV. Specifically, computed S_0_ on the basis of the RPBE DF and a dynamical model in which energy transfer was allowed to surface atom vibrations and ehp excitation gave good agreement^117^ with the best estimates of measured S_0_.^119^ Table S1 in the Supporting Information presents data concerning the dependence of the computed barrier height on the pseudopotentials used for this system. In the calculations presented here, we used for both N- and Ru- atoms a hard pseudopotential, i.e. *Ru*_*pv*_ and *N*_*h*_. As the reference value for our database, we will use 1.84 eV, which value was obtained using a hard pseudopotential for Ru (*Ru*_*pv*_), but an ordinary pseudopotential for N(*N*)^116^ in the DFT calculations performed to produce the PES underlying the good agreement with experiment.

#### N_2_ + Ru(101̅0)

Because of the absence of SRP-DFT data for N_2_ + Ru(101̅0), as was done in the original SBH10 paper,^40^ we use a reference value of 0.4 eV for the barrier height. Note that this value actually represents an activation energy obtained from thermal rate measurements on the DC of N_2_ on Ru(0001),^77,120^ suggesting that the barrier height contains zpe corrections. Another presumption implicitly used in refs (77 and 120), and therefore in ref (40), is that the activation energy derived from measurements^77,120^ on (necessarily defected) Ru(0001) should be the same as the activation energy that would be obtained for Ru(101̅0), i.e., that the steps occurring on the latter surface have the same promoting effect on the reaction on Ru(0001) as do the unspecified defects on Ru(0001).

#### II.3.c CH_4_ Dissociation on Transition Metals

The DC of CH_4_ on metal surfaces is important to industry as it constitutes the first step in the steam reforming of natural gas, producing CO, which can be used for alcohol synthesis and for the Fischer–Tropsch process, and hydrogen, which can be used as a fuel and for ammonia production. The dissociation of CH_4_ on metal surfaces has been the subject of many theoretical^13,14,121−124^ and experimental studies.^13,14,125−135^

#### CH_4_ + Ni(111)

CH_4_ + Ni(111) is the first CH_4_ on the metal system for which an SRP-DF was derived.^13^ The generic expression given by eq 2 was employed, using a weighted average of the RPBE (32%) and the PBE DF (68%) combined with nonlocal vdW-DF1.^44^ This SRP-DF (SRP32-vdW-DF1^13^) was fitted to laser-off experiments performed on CHD_3_ + Ni(111) for T_*N*_ = 600 and 650 K using DFMD calculations. Subsequent DFMD calculations also reproduced measured S_0_ for CH-stretch excited CHD_3_ on Ni(111) with chemical accuracy. The barrier height that was computed with an appropriate residual energy correction for the vacuum distance was 1.015 eV^13^ (Table 2, see also table S6 of ref (14)). This is the reference value that should be used for calculations in which CH_4_ is placed far enough from the surface to obtain a value of the asymptotic energy that is converged with respect to the vacuum length^13^ (i.e, the value of E_*b*_^*e*^ in table S6 of ref (14), see also the discussion in Section 3.1 of the Supporting Information to ref (14)). This is the reference value we use to compare results to those that were computed with the GGA and meta-GGA calculations, as with these DFs the asymptotic energy is converged with respect to the vacuum length used in our calculations. For the calculations with vdW-DF1 and vdW-DF2 correlation DFs, we take into account that a correction has to be applied for the fact that in the present calculations the vacuum distance was too short (at 13 Å) and the molecule was too close to the surface (at 6 Å) for these DFs. Instead, for these DFs, we use the value of E_*b*_^13^ quoted in table S6 of ref (14) (i.e, 1.055 eV, see Table 2).

#### CH_4_ + Ni(100)

Sticking of CH_4_ on Ni(100) has been simulated with quantum dynamics calculations explicitly modeling motion in eight molecular degrees of freedom,^66^ with QCT calculations^136^ and with reaction path Hamiltonian (RPH) calculations.^136−140^ In none of these calculations, agreement with existing molecular beam experiments was achieved to within chemical accuracy. Therefore, for this system, we instead use the same reference value of the barrier height as the value quoted in the previous SBH10 database.^40^ However, we note that the earlier paper gave an incomplete explanation of how this value (0.76 eV) of the barrier height was obtained in the paper referenced.^122^ The value used refers to the barrier height employed in calculations^122^ with a three-dimensional dynamical model augmented with the so-called hole model,^80^ which approximately reproduced previously measured S_0_.^125^ The value quoted for the minimum barrier height (0.76 eV) is in fact not a minimum barrier height in the model employed in ref (122) but rather the barrier height averaged over the impact points on the surface and the orientations of the dissociating molecule. We will analyze the consequences of this misinterpretation below and make a recommendation as to whether and how this value should be replaced in a future version of the database.

#### CH_4_ on Ni(211)

The SRP32-vdW-DF1 developed for CH_4_ on Ni(111) has also been used in RPH calculations on sticking of CH_4_ + Ni(211).^123^ However, molecular beam sticking experiments are not yet available for this system. A recent study of Guo and Jackson^124^ also reported computed thermal S_0_ for step and terrace sites calculated for CH_4_ on Ni(211) with harmonic and anharmonic transition state theory. It was possible to compare these results to analogous results extracted from experiments on CH_4_ + Ni(14 13 13),^141^ which surface also consists of (100) steps and (111) terraces, albeit that the terraces are much wider than on Ni(211). Excellent agreement was obtained for the sticking at the step sites, suggesting that the SRP-DF for CH_4_ + Ni(111) should also describe sticking of methane on Ni surfaces consisting of (111) terraces and (100) steps (like Ni(211)) with chemical accuracy. For our benchmark study, we will use therefore as the reference value the minimum barrier height reported by Jackson and co-workers for the DC at the steps of Ni(211), which is 0.699 eV.

#### CH_4_ + Pt(111) and Pt(211)

For the DC of CH_4_ on metals, several cases of transferability were observed. DFMD calculations with the SRP32-vdW-DF1 functional developed for CHD_3_ on Ni(111) also reproduced molecular beam sticking experiments on CHD_3_ + Pt(111) and Pt(211) with chemical accuracy.^14^ The barrier heights reported for these two systems, again including a residual energy correction for the short vacuum distance and the short distance of the methane to the surface in the initial state used in the DFMD calculations, are E_*b*_^*e*^ = 0.815 eV^14^ and 0.559 eV, and these are the reference values we use when testing GGA and meta-GGA DFs.^13,142^ As for CH_4_ + Ni(111), for our benchmark purposes, when testing DFs with vdW-DF1 and vdW-DF2 correlation, we will use the values with residual energy correction (0.856 and 0.581 eV, respectively) as reported by Migliorini et al.^14^ (table S6 of ref (14) and Table 3 of ref (142)).

#### CH_4_ + Ir(111)

As was the case for CH_4_ + Ni(211), the SRP32-vdW-DF1 developed for CH_4_ + Ni(111) has also been used in RPH dynamics calculations on CH_4_ + Ir(111).^121^ The S_0_ computed with this method for sticking of CH_4_ in its vibrational ground state has been compared with values measured in molecular beam experiments.^126,128,134^ An analysis of how these data compare (see Figure 67 of ref (10)) shows that the RPH dynamics calculations reproduce the measured S_0_ with chemical accuracy. For this system, we therefore used the barrier height reported by ref (121), which is 0.836 eV, as the reference value.

#### CH_4_ + Ru(0001)

As already noted in the SBH10 paper,^40^ this reference value was extracted from experiments on laser assisted associative desorption (LAAD).^135^ Specifically, the “adiabatic minimum barrier height V*(0)” was extracted from the experiments by taking temperature dependent values of the highest CH_4_ translational energy observed as a function of the surface temperature (T_*s*_) and extrapolating the maximum translational energy observed to T_*s*_ = 0 K. While this gave values not too different from the V*(0) values extracted in an approximate fashion^135^ from earlier molecular beam sticking experiments^127^ and from earlier DFT calculations,^135,143^ the method used was approximate. Moreover it is clear from the paper^135^ that the V*(0) value should be interpreted as an activation energy, i.e., in DFT it would be the minimum barrier height with zpe corrections added.

### II.4 Algorithms for Computing Minimum Barrier Heights

The minimum barrier height to the DC may be computed with DFT as
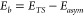
4Here, *E*_*TS*_ is the energy of the system with the molecule at the transition state (TS) or minimum barrier geometry, and *E*_*asym*_ is the energy of the system with the molecule in its equilibrium gas phase geometry and far enough from the surface that the molecule and surface no longer interact with each other. This coincides with an approach that is usually taken to extract barrier heights from PESs used in dynamics calculations. We also suggest that this approach might benefit from cancellation of errors, which might not result if the energies of the reactants (the bare surface and the incoming molecule) are calculated separately, in calculations that might differ in the size of the supercell and k-points used. In any case, the asymptotic state will somehow have to be represented in the PES used for the dynamics calculations, so that it makes sense to compute it in the same manner as the minimum barrier height.

Ideally, these geometries would be known to high accuracy from theory or experiment. While this is true for the equilibrium geometry of the small molecules investigated here and usually also for the structure of the metal surfaces investigated here, it is not true for the transition state geometries. In this sense, the field of molecule-metal surface chemistry differs from that of gas phase chemistry,^23−26^ where transition state geometries of at least small systems are often well-known from accurate ab initio (CCSD(T)^27^) calculations. When benchmarking electronic structure methods on gas phase systems, the availability of CCSD(T) geometries carries the advantage with it that only single point calculations have to be performed and that geometry optimizations can be omitted.

This is not the case for calculations on the DC on metals. Choices have to be made regarding several issues. These issues are (i) how to choose the equilibrium gas phase geometry of the molecule, (ii) how to choose the geometry of the molecule in the transition state, and (iii) how to choose the geometry of the metal surface in the TS and asymptotic geometries. In this work, we have tested how the results depend on different choices regarding these issues. We have tested this using three algorithms, which we called high, medium, and light according to the computational effort associated with the algorithms.

#### II.4.a Light Algorithm

Calculations with the light algorithm are the least expensive as only single point calculations are involved. The following choices are made: (i) the experimental equilibrium geometry of the molecule is used for the asymptotic state, (ii) the TS geometry of the molecule relative to that of the surface is taken as the SRP-DFT geometry of the molecule relative to the metal surface (see Table 2), and (iii) the metal surface is built up by simply using the experimental lattice constant at 0 K, without relaxation of the interlayer distances in the slab.

#### II.4.b Medium Algorithm

In the case of the medium algorithm, for (ii) the same choice is made for the geometry of the molecule relative to the surface in the system’s TS geometry as in the light algorithm. However, for (i) and (iii), different choices are made: the molecule’s equilibrium geometry is now computed on the basis of the DF tested, and the lattice constant of the metal surface as well as the relaxed interlayer distances of the metal surface at the interface with the vacuum are now also optimized separately for each functional tested. This takes into account that the lattice constant and the relaxed interlayer distances may depend strongly on the DF tested,^144^ while in turn the minimum barrier height may depend rather strongly on the parameters determining the geometry of the metal surface. The dependence of the minimum barrier height on the geometry of the metal surface is relevant to DFMD calculations,^145,146^ as incorrect initial geometries of the metal may lead to surface strain, which can in turn affect the barrier height to the DC.^147^ In the medium as well as in the high algorithm below, the geometry of the metal surface in the TS is taken the same as that in the asymptotic state, as the metal surface atoms will usually not have time to respond to the fast incoming motion of the molecule in the hypersonic molecular beam experiments to which comparison is made for assessing the accuracy of SRP DFs.^10^ We note that for CH_4_ the molecule’s geometry has only been optimized once, with the RPBE functional, and the RPBE geometry was used with all other DFs. Table S2 shows that this leads to errors no greater than 5 meV.

#### II.4.c High Algorithm

The high algorithm differs from the medium algorithm only in that now the TS geometry of the molecule relative to the surface is determined by geometry optimization using the dimer method as implemented in the VASP Transition State Tools (VTST) package.^148−151^ As stated above, in the TS search process, the metal surface was kept frozen in its relaxed 0 K geometry. The optimization of the TS geometry of the molecule was stopped when the maximum force on any degree of freedom was smaller than 5 meV/Å. All the TS geometries reported here have been confirmed to be the first-order saddle points in the molecular coordinate space by frequency analysis (by checking that one and only one imaginary frequency was found).

### II.5 Computational Details

All the new calculations presented here are performed using the Vienna *ab initio* simulation package^152−155^ (Vasp5.4.4). The calculations with DFs incorporating vdW-DF1^44^ or vdW-DF2^45^ correlation have therefore been performed with the Vasp implementation of these DFs,^50^ except the calculations with the BEEF-vdW-DF2 DF,^16^ for which the libbeef library^156^ was used. Through the way these DFs were implemented, they all inherit the LDA correlation from the PBE DF,^46^ which means that the PW92 variant of the LDA correlation^157^ is used. All calculations with vdW-DF1 or vdW-DF2 were performed with the algorithm due to Román-Pérez and Soler,^158^ which speeds up the evaluation of these DFs. Because of the amount of the calculations that had to be done, the Atomic Simulation Environment (ASE) was used as a convenient interface package.^159,160^ Typically, the default projected augmented wave (PAW) pseudopotentials were used; however, for N_2_ + Ru(0001) and N_2_ – Ru(101̅0), we used hard core pseudopotentials: ***Ru***_*pv*_ (a ***Z***_*n*_ core pseudopotential leaving 14 of the electrons of ***Ru*** in its 4*p*^6^5*s*^1^4*d*^7^ configuration to be modeled) and ***N***_*h*_ (a ***H***_*e*_ core pseudopotential leaving 5 electrons of ***N*** in its 2*s*^2^2*p*^3^ configuration to be modeled). For all systems containing a ***Ni*** surface, spin polarization has been taken into account. A complete description of the input parameters (e.g., number of metal layers in the metal slab, size of the surface unit cell, the plane wave cutoff energy, the number of k-points, the vacuum distance, etc.) used in this work can be seen in Table S3 of the Supporting Information. In the optimization of the metal slab, for all systems, we used a 1 × 1 surface unit cell and kept the bottom layer frozen, and the upper n-1 layers of the metal surface were allowed to relax. For the 3 systems for which only ad-hoc SE results are available (CH_4_ + Ru(0001), CH_4_ + Ni(100), and N_2_ + Ru(101̅0)), the geometries we used for the medium and light algorithms were obtained from the calculations where we used the high algorithm based on the SRP32-vdW-DF1 for CH_4_ on metal systems and on the RPBE DF for N_2_ – Ru(101̅0).

## III Results

### III.1 Structure of the Metals

Table 3 presents, for all metals in the database, the calculated lattice constants as computed with all DFs tested, comparing with zpe corrected experimental values^161,162^ and also showing the MAE and MSE with respect to the experiment for each DF. The lowest MAEs are found for the meta-GGA DFs, and the highest MAEs are found for the DFs consisting of GGA exchange but vdW-DF1 or vdW-DF2 correlation, with the vdW-DF2 functional exhibiting the poorest performance. For this property, the GGA-DFs are found to be of intermediate accuracy.

**Table 3:** Comparison of Metal Lattice Constants Computed in This Work with Experiment and with Other Computational Results^a^

	Ag	Ir	Cu	Pt	Ni	Ru			
						a	c		
Exp	4.062^161^	3.831^161^	3.597^161^	3.912^161^	3.499^161^	2.703^162^	4.274^162^		
functional								MAE	MSE
PBE	4.146^†^	3.889^†^	3.624^†^	3.985^†^	3.519^†^	2.722^†^	4.293^†^	0.0506	0.0506
	4.152^161^	3.887^161^	3.632^161^	3.985^161^	3.518^161^	2.73^51^	4.304^51^		
		3.9^193^							
		3.873^194^							
		3.877^195^							
RPBE	4.201^†^	3.903^†^	3.673^†^	4.010^†^	3.553^†^	2.734^†^	4.315^†^	0.0824	0.0824
		3.908^193^				2.744^51^	4.325^51^		
		3.891^195^							
SRP50	4.173^†^	3.896^†^	3.648^†^	3.997^†^	3.535^†^	2.727^†^	4.304^†^	0.0678	0.0678
vdW-DF1	4.226^†^	3.934^†^	3.703^†^	4.052^†^	3.573^†^	2.753^†^	4.338^†^	0.115	0.115
						2.761^51^	4.351^51^		
vdW-DF2	4.288^†^	3.988^†^	3.775^†^	4.126^†^	3.615^†^	2.791^†^	4.398^†^	0.176	0.176
		3.987^195^							
SRP32-vdW-DF1	4.203^†^	3.928^†^	3.680^†^	4.042^†^	3.558^†^	2.747^†^	4.330^†^	0.100	0.100
		3.923^195^							
PBE-vdW-DF2	4.204^†^	3.927^†^	3.681^†^	4.040^†^	3.518^†^	2.747^†^	4.330^†^	0.092	0.092
						2.754^51^	4.341^51^		
PBEα57-vdW-DF2	4.173^†^	3.918^†^	3.653^†^	4.025^†^	3.537^†^	2.739^†^	4.319^†^	0.0792	0.0792
	4.176^196^			4.015^52^					
BEEF-vdW-DF2	4.196^†^	3.899^†^	3.656^†^	4.014^†^	3.536^†^	2.730^†^	4.306^†^	0.0782	0.0782
optPBE-vdW-DF1	4.160^†^	3.907^†^	3.641^†^	4.007^†^	3.526^†^	2.730^†^	4.306^†^	0.066	0.066
revTPSS	4.064^†^	3.851^†^	3.561^†^	3.927^†^	3.457^†^	2.700^†^	4.268^†^	0.0244	–0.010
						2.69^51^	4.246^51^		
SCAN	4.091^†^	3.808^†^	3.580^†^	3.906^†^	3.460^†^	2.696^†^	4.249^†^	0.0246	–0.013
MS-B86bl	4.101^†^	3.841^†^	3.583^†^	3.907^†^	3.472^†^	2.700^†^	4.260^†^	0.0208	–0.001
	4.092^55^		3.583^55^	3.906^55^					
MS2	4.0745^†^	3.8407^†^	3.5543^†^	3.9115^†^	3.4498^†^	2.7002^†^	4.2631^†^	0.0248	–0.016

a Lattice constants computed in this work are marked with a “^†^” and listed for each tested DF, also providing other computational results for the DFs tested where available. The experimental values (Exp) have been corrected for zpe effects. The MAE and MSE represent the means of the absolute and signed deviations of the lattice constants computed in this work from the experimental values, for each DF tested. All results are in Å.

Table 4 shows, for each DF tested, the computed percentage change of the distance between the top two layers of the relaxed (111) metal surface relative to the ideal bulk interlayer distance, for the (111) surfaces relevant to SBH17, also comparing to the corresponding experimental results. Again, the best results are found with the meta-GGA DFs. For instance, with the revTPSS DF, the correct sign was found for all four metal surfaces for which experimental results are available. The GGA DFs get the sign wrong for Pt(111), while the functionals with vdW-DF1 and vdW-DF2 correlation all get the sign wrong for Ag(111). With the functionals and input parameters used, neither experiment nor other DFT calculations presented in Table 4 are quantitatively reproduced.

**Table 4:** Comparison of Computed and Measured Results Characterizing Surface Relaxation^a^

	Ag	Ir	Cu	Pt	Ni
Exp	–2.5%^197^		–1.0%^198^	1.1%^199^	–0.07%^198^
	–0.5%^200^		–0.7%^201^		
			GGA		
PBE	–0.34^†^	–2.66^†^	–0.26^†^	–0.07^†^	–1.38^†^
	–0.20^144^		–0.30^144^	0.90^144^	
	–0.30^165^			0.90^165^	
RPBE	0.38^†^	–2.58^†^	–0.47^†^	–0.05^†^	–0.80^†^
SRP50	–0.04^†^	–2.62^†^	–0.33^†^	–0.06^†^	–1.32^†^
			GGA+vdW		
vdW-DF1	1.19^†^	–2.37^†^	–0.38^†^	0.00^†^	–1.26^†^
	0.10^196^		–0.20^196^	1.30^196^	–1.10^196^
vdW-DF2	2.24^†^	–1.99^†^	–1.63^†^	0.31^†^	–1.46^†^
	0.50^196^		0.00^196^	1.50^196^	–1.10^196^
SRP32-vdW-DF1	0.73^†^	–2.44^†^	–0.20^†^	–0.06^†^	–1.21^†^
PBE-vdW-DF2	0.77^†^	–2.42^†^	–0.13^†^	–0.07^†^	0.66^†^
PBEα57-vdW-DF2	0.51^†^	–2.14^†^	–0.02^†^	–0.10^†^	–1.16^†^
	0.00^196^		–0.40^196^	–0.80^196^	–0.80^196^
BEEF-vdW-DF2	0.54^†^	–2.51^†^	–0.09^†^	0.03^†^	–1.17^†^
			meta-GGA		
revTPSS	–0.86^†^	–2.81^†^	–0.31^†^	0.35^†^	–0.92^†^
SCAN	–0.95^†^	–2.70^†^	–0.99^†^	2.39^†^	–1.57^†^
	–0.40^144^		–0.40^144^	2.50^144^	
MS-B86bl	–0.73^†^	–2.76^†^	–0.85^†^	1.16^†^	–1.00^†^
	–0.50^55^		–1.00^55^	1.00^55^	
MS2	–0.54^†^	–2.77^†^	–0.94^†^	0.4^†^	–0.96^†^

a The relaxation of the interlayer lattice spacing between the upper two layers of the surface relative to the bulk value is given in % for all (111) surfaces relevant to the SBH17 database and for all DFs tested in this work, also comparing to experimental results (Exp) and other DFT results where available. Values computed in this work are marked with a “^†^”.

### III.2 Dissociative Chemisorption Barriers

**Figure 1:**
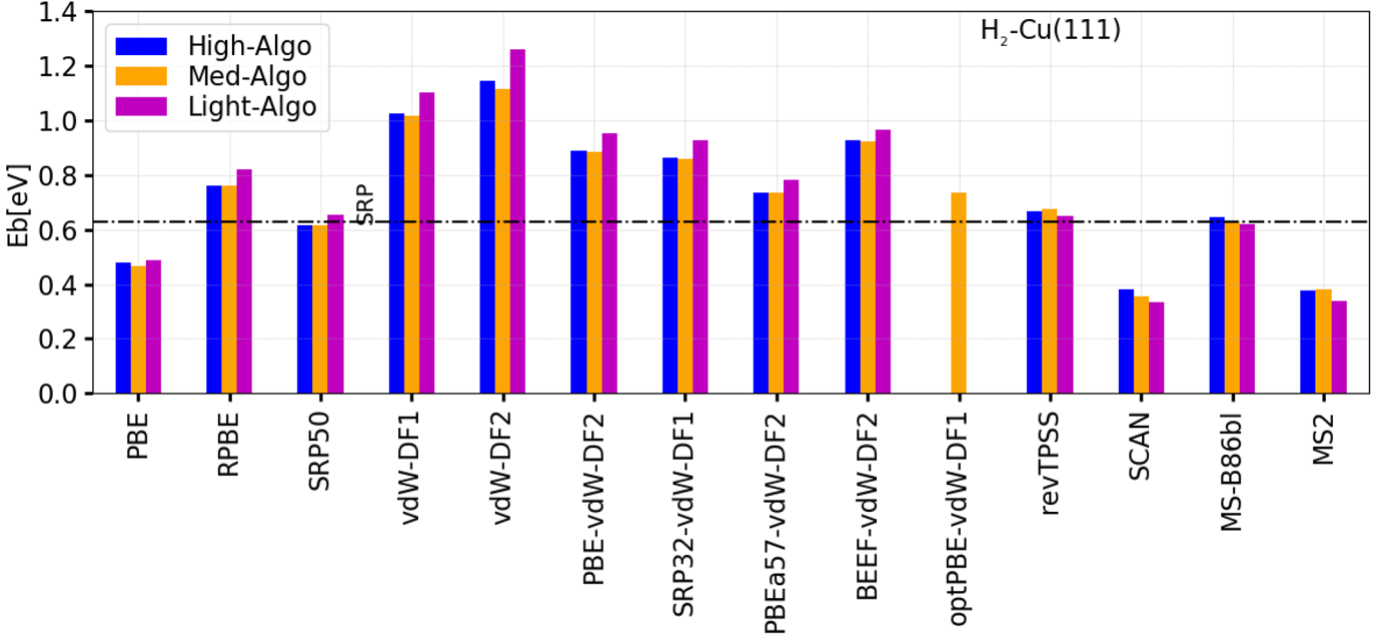
Performance of the DFs and algorithms tested on the DC of H_2_ on Cu(111). Computed barrier heights are compared with the reference value for this system, which is indicated by the horizontal dot-dashed line (see also Table 2).

To give an idea of the size of the error that may arise from the DF and algorithm used for a particular system, Table 5 and Figure 1 present the barrier heights computed for H_2_ + Cu(111) (The barrier heights for the other systems in the database and geometries can be found in Tables S4 to S19 and Figures S1 to S16 of the Supporting Information.). With the medium algorithm, three DFs (SRP50, revTPSS, and MS-B86bl) yield barrier heights close to the SRP reference value of 0.636 eV.^11^ However, other DFs yield barriers that are far off the mark, with the largest overestimate (by 0.48 eV) coming from the vdW-DF2 and the largest underestimate (by 0.28 eV) coming from the SCAN functional.

**Table 5:** Barrier Heights for H_2_ + Cu(111) (in eV) for All the DFs and Algorithms Tested^a^

functional	high algo	light algo	medium algo	literature values
		GGA		
PBE	0.478	0.488	0.467	0.484(CRP)^101^
RPBE	0.762	0.819	0.762	0.797(CRP)^202^
SRP50	0.618	0.654	0.616	0.636(SRP48)^202^
		GGA+vdW		
vdW-DF1	1.026	1.102	1.019	1.004(CRP)^202^
vdW-DF2	1.144	1.260	1.117	
PBE-vdW-DF2	0.889	0.952	0.885	0.863(CRP)^202^
SRP32-vdW-DF1	0.863	0.926	0.860	
PBEa57-vdW-DF2	0.736	0.781	0.735	0.72(CRP)^202^
BEEF-vdW-DF2	0.928	0.966	0.925	
optPBE-vdW-DF1			0.736	
		meta-GGA		
revTPSS	0.667	0.648	0.674	0.605(CRP)^202^
SCAN	0.382	0.334	0.354	0.398(CRP)^202^
MS-B86bl	0.647	0.619	0.634	0.683(CRP)^55^
MS2	0.378	0.340	0.382	

a Values marked with “CRP” come from an accurate fit of the H_2_ + Cu(111) PES to DFT data computed with the DF listed.^202^

Table 6 shows MAEs and MSEs for all algorithms and DFs. To compare the results obtained with different algorithms, the average is always taken over the number of systems for which reliable saddle point geometries could be obtained with the high algorithm for a given DF. As Table 6 shows, with the high algorithm, reliable saddle point geometries were obtained for 16 systems using the PBE, SRP50, and the MS-B86bl DFs, for 15 systems using the SCAN DF, and for all 17 systems for all remaining DFs. Table 6 shows that in general the errors obtained with the medium algorithm are close to those obtained with the high algorithm, which is much more CPU intensive. Interestingly, this was not true for the majority of the meta-GGA DFs: for these DFs, the medium and high algorithms only give similar results for the revTPSS DF.

**Table 6:** Performance of the DFs and Algorithms Tested on the SBH17 Database^a^

			high algo		med algo		light algo		
N_*ex*_	functional	type	MAE	MSE	MAE	MSE	MAE	MSE	N_*s*_ system missing
1	PBE	GGA	0.116	–0.075	0.106	–0.065	0.148	–0.067	5
0	RPBE	GGA	0.230	0.230	0.228	0.228	0.263	0.263	
1	SRP50	GGA	0.126	0.081	0.127	0.085	0.161	0.102	5
0	vdW-DF1	GGA+vdW	0.230	0.230	0.220	0.220	0.297	0.297	
0	vdW-DF2	GGA+vdW	0.329	0.329	0.311	0.311	0.461	0.461	
0	optPBE-vdW-DF1	GGA+vdW			0.131	–0.033			
0	PBEα57-vdW-DF2	GGA+vdW	0.139	–0.053	0.124	–0.040	0.135	–0.002	
0	SRP32-vdW-DF1	GGA+vdW	0.127	0.060	0.115	0.057	0.170	0.119	
0	PBE-vdW-DF2	GGA+vdW	0.144	0.109	0.141	0.112	0.191	0.166	
0	BEEF-vdW-DF2	GGA+vdW	0.190	0.190	0.191	0.191	0.228	0.222	
2	SCAN	meta-GGA	0.185	–0.172	0.154	–0.120	0.249	–0.242	5, 7
0	revTPSS	meta-GGA	0.147	–0.060	0.146	–0.025	0.165	–0.105	
0	MS2	meta-GGA	0.196	–0.176	0.117	–0.074	0.173	–0.149	
1	MS-B86bl	meta-GGA	0.173	0.157	0.214	0.199	0.191	0.133	5
average			0.179	0.065	0.166	0.075	0.218	0.092	

a Mean absolute errors (MAEs) and mean signed errors (MSEs, both in eV) measure average deviations of the barrier heights computed with each DF and algorithm from the reference values listed in Table 2. N_*ex*_ represents the number of systems that had to be excluded for specific DFs, and N_*s*_ their numbers (see Table 2 and the text).

Table 7 shows the MAEs and MSEs for all DFs tested with averaging over all 17 systems, using the medium algorithm. With the MSE as accuracy criterion, the revTPSS meta-GGA comes out as the best for DC barrier heights. The next three highest-ranked DFs all combine GGA exchange with vdW-DF1 or vdW-DF2 correlation, with the optPBE-DF1 showing the best performance. The PBE DF ranks fifth and is the best performing GGA DF. If the DFs are ranked according to their performance for the MAE, the PBE DF actually performs best, with SRP32-vdW-DF2 coming out second, and the MS2 meta-GGA DF ranking third, thereby outperforming the revTPSS meta-GGA, which now ranks ninth.

**Table 7:** Performance of the DFs Tested on the SBH17 Database Using the Medium Algorithm^a^

	med algo			
functional	MAE	*r*_MAE_	MSE	*r*_|MSE|_
PBE	0.103	1	–0.058	5
RPBE	0.228	13	0.228	13
SRP50	0.125	5	0.085	7
vdW-DF1	0.219	12	0.219	12
vdW-DF2	0.312	14	0.312	14
PBE-vdW-DF2	0.141	8	0.112	9
SRP32-vdW-DF1	0.115	2	0.057	4
PBEα57-vdW-DF2	0.124	4	–0.040	3
BEEF-vdW-DF2	0.191	10	0.191	10
optPBE-vdW-DF1	0.131	6	–0.033	2
revTPSS	0.146	9	–0.025	1
SCAN	0.140	7	–0.105	8
MS-B86bl	0.210	11	0.195	11
MS2	0.117	3	–0.074	6
average	0.164		0.076	

a The MAE and MSE (in eV) are computed with averaging over all 17 systems. The values of *r*_MAE_ and *r*_|MSE|_ rank the DFs according to best performance for the MAE and |MSE| error criteria, respectively.

Table 8 shows the performance of the DFs for the smaller and older SBH10 database. The three DFs featuring GGA exchange and vdW-DF1 or vdW-DF2 correlation that performed well for the SBH17 database with the absolute value of the MSE as the accuracy criterion again do well, with SRP32-vdW-DF now ranking first. The PBE performance is also consistent, with PBE ranking fifth, but as a GGA DF, PBE is now outperformed by SRP50, which takes third place. The DFs performing well in terms of their absolute value of the MSE also do well on the MAE for SBH10.

**Table 8:** Performance of the DFs and Algorithms Tested on the SBH10 Database^a^

		high algo			med algo		light algo		SBH10^40^
functional	type	MAE	MSE	|MSE|	MAE	MSE	MAE	MSE	MAE/MSE
SRP32-vdW-DF1	GGA+vdW	0.134	0.041	0.041	0.126	0.034	0.151	0.066	
SRP50	GGA	0.132	0.063	0.063	0.133	0.067	0.155	0.061	
optPBE-vdW-DF1	GGA+vdW			0.074	0.141	–0.074			
PBEα57-vdW-DF2	GGA+vdW	0.135	–0.076	0.076	0.132	–0.073	0.143	–0.060	
PBE-vdW-DF2	GGA+vdW	0.142	0.093	0.093	0.148	0.098	0.163	0.120	
PBE	GGA	0.149	–0.098	0.098	0.143	–0.091	0.170	–0.112	
revTPSS	meta-GGA	0.198	–0.124	0.124	0.177	–0.117	0.237	–0.185	
MS-B86bl	meta-GGA	0.196	0.165	0.165	0.154	0.123	0.173	0.064	
SCAN	meta-GGA	0.198	–0.178	0.178	0.179	–0.156	0.253	–0.245	
BEEF-vdW-DF2	GGA+vdW	0.182	0.182	0.182	0.179	0.179	0.193	0.183	0.12/0.03
MS2	meta-GGA	0.220	–0.188	0.188	0.171	–0.117	0.223	–0.198	0.36/–0.34
vdW-DF1	GGA+vdW	0.214	0.214	0.214	0.211	0.211	0.256	0.256	
RPBE	GGA	0.223	0.223	0.223	0.224	0.224	0.235	0.235	
vdW-DF2	GGA+vdW	0.331	0.331	0.331	0.324	0.324	0.435	0.435	
average		0.189	0.050		0.174	0.045	0.214	0.047	

a Both MAE and MSE (in eV) are calculated by including, and averaging over, the ten systems present in the previous SBH10^40^ database. The DFs have been ranked according to the best performance of the high algorithm according to the |MSE| criterion. For the BEEF-vdW-DF2 and MS2 DFs, we also present the values computed earlier while allowing surface relaxation in the TS.^40^

**Figure 2:**
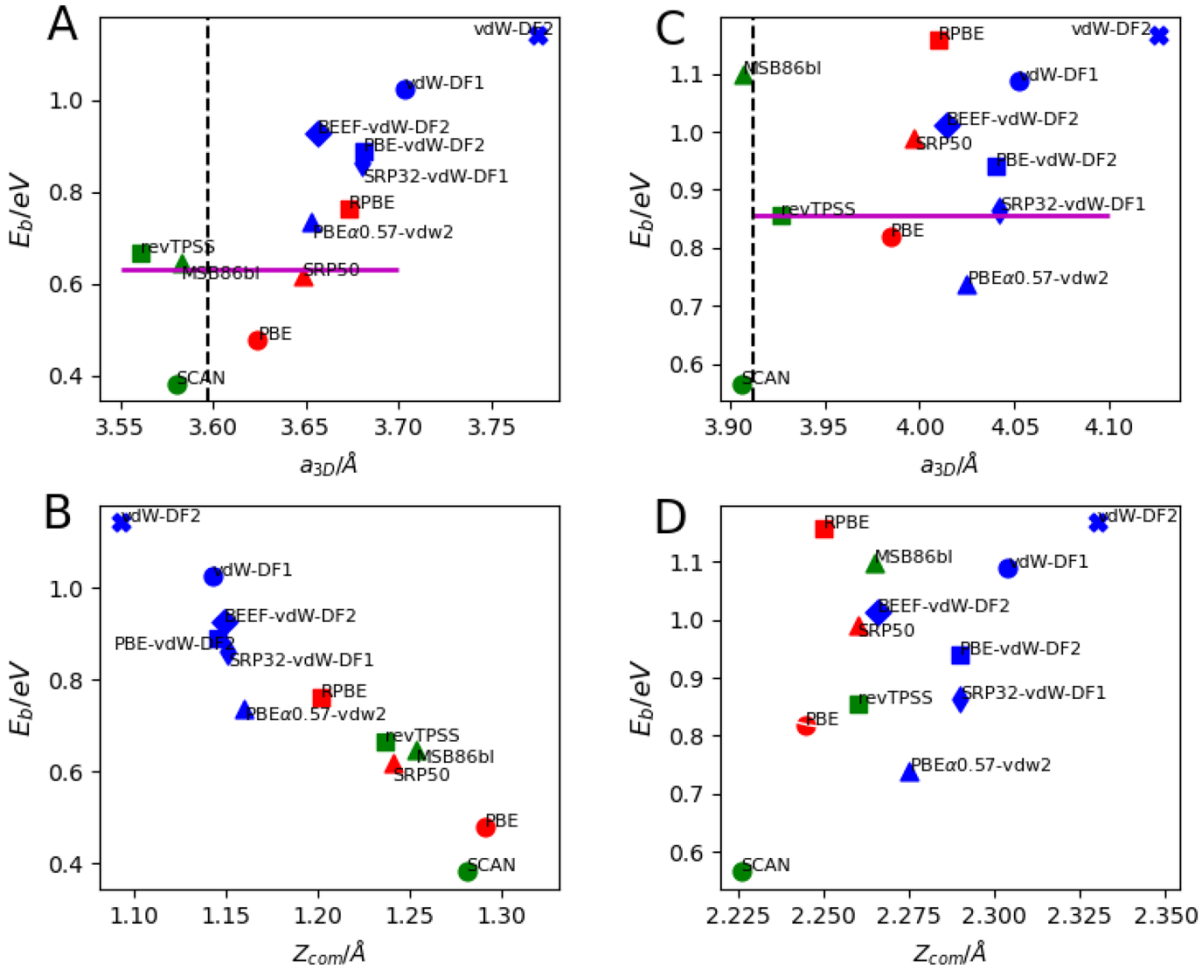
Correlation of the barrier height for the DC with the optimized lattice constant (a_3*D*_) of the metal (upper panels) and of the barrier height with the distance of the molecule to the surface at the transition state (Z_*com*_), as computed with all DFs tested in this work. The high algorithm was used. The left panels present results for H_2_ + Cu(111), and the right panels present results for CH_4_ + Pt(111). The vertical black dashed lines in the upper panels represent the experimental lattice constants, and the horizontal magenta solid lines in the upper panels represent the reference values of the barrier heights.

The top panels of Figure 2 present the correlation of the minimum barrier height of the whole system with the computed lattice constant of the metal for the DFs tested, also comparing to the SE and the experimental values of these parameters, respectively, for H_2_ + Cu(111) and CH_4_ + Pt(111). The bottom panels show the correlation of the computed minimum barrier height with the distance of the molecule to the surface in the optimized minimum barrier geometry for these two systems. An interesting feature of the revTPSS DF is that it predicts both the lattice constant of the metal and the minimum barrier height with reasonably high accuracy, while the computed distance of the molecule to the metal surface also agrees well with that obtained using the SRP-DFT approach.

Table 9 presents the errors made with the medium algorithm for the 8 H_2_-metal systems in the database (see also Figures S17 and S18). For these systems and with the absolute value of the MSE as accuracy criterion, the PBE GGA DF does best, with the SRP50 DF as the runner up. The three DFs in which GGA exchange was combined with nonlocal correlation and which did well for SBH17 also do reasonably well for the H_2_-metal reactions. The same is true for revTPSS which came out as best for SBH17 but is not best for the H_2_-metal systems. Table 10 presents the errors made with the medium algorithm for the 2 N_2_-metal systems in the database (see also Figures S19 and S20). For these systems, DFs that did well for SBH17 generally are not very good. MS-B86bl, BEEF-vdW-DF2, and RPBE perform best for the N_2_-metal systems. Table 11 presents the errors made with the medium algorithm for the 7 CH_4_-metal systems in the database (see also Figures S21 and S22). The DFs that did well for SBH17 also do reasonably well for the CH_4_ + metal systems. However, for the latter category, SCAN is now the best performing DF using the MSE as accuracy criterion. Using the MAE as accuracy criterion, the best CH_4_-metal results are obtained with the SRP32-vdW-DF1, PBE, and revTPSS DFs, respectively.

**Table 9:** Performance of the DFs Tested on the 8 H_2_-Metal Systems Present in the SBH17 Database Using the Medium Algorithm^a^

	med algo			
functional	MAE	*r*_MAE_	MSE	*r*_|MSE|_
PBE	0.080	2	–0.049	1
RPBE	0.167	10	0.167	10
SRP50	0.070	1	0.063	2
vdW-DF1	0.264	13	0.264	13
vdW-DF2	0.290	14	0.290	14
PBE-vdW-DF2	0.174	11	0.174	11
SRP32-vdW-DF1	0.152	9	0.147	9
PBEα57-vdW-DF2	0.090	5	0.071	3
BEEF-vdW-DF2	0.227	12	0.227	12
optPBE-vdW-DF1	0.096	6	0.091	6
revTPSS	0.086	4	0.086	5
SCAN	0.121	7	–0.117	7
MS-B86bl	0.128	8	0.128	8
MS2	0.084	3	–0.084	4
average	0.145		0.104	

a The MAE and MSE (in eV) are computed with averaging over all 8 systems. The values of *r*_MAE_ and *r*_|MSE|_ rank the DFs according to best performance for the MAE and |MSE| error criteria, respectively.

**Table 10:** Performance of the DFs Tested on the 2 N_2_-Metal Systems Present in the SBH17 Database Using the Medium Algorithm^a^

	med algo			
functional	MAE	*r*_MAE_	MSE	*r*_|MSE|_
PBE	0.409	10	–0.409	10
RPBE	0.088	3	0.088	4
SRP50	0.157	6	–0.157	6
vdW-DF1	0.048	2	0.048	3
vdW-DF2	0.372	8	0.372	8
PBE-vdW-DF2	0.123	5	–0.123	5
SRP32-vdW-DF1	0.217	7	–0.217	7
PBEα57-vdW-DF2	0.378	9	–0.378	9
BEEF-vdW-DF2	0.026	1	0.026	2
optPBE-vdW-DF1	0.434	11	–0.434	11
revTPSS	0.723	14	–0.723	14
SCAN	0.525	13	–0.525	13
MS-B86bl	0.102	4	–0.024	1
MS2	0.454	12	–0.454	12
average	0.290		–0.208	

a The MAE and MSE (in eV) are computed with averaging over all 2 systems. The values of *r*_MAE_ and *r*_|MSE|_ rank the DFs according to best performance for the MAE and |MSE| error criteria, respectively.

**Table 11:** Performance of the DFs Tested on the 7 CH_4_-Metal Systems Present in the SBH17 Database Using the Medium Algorithm^a^

	med algo			
functional	MAE	*r*_MAE_	MSE	*r*_|MSE|_
PBE	0.045	2	–0.016	2
RPBE	0.336	14	0.336	14
SRP50	0.177	9	0.177	9
vdW-DF1	0.218	11	0.218	11
vdW-DF2	0.319	12	0.319	12
PBE-vdW-DF2	0.108	8	0.108	8
SRP32-vdW-DF1	0.040	1	0.032	3
PBEα57-vdW-DF2	0.090	7	–0.071	7
BEEF-vdW-DF2	0.196	10	0.196	10
optPBE-vdW-DF1	0.086	6	–0.060	6
revTPSS	0.05	3	0.047	5
SCAN	0.077	5	–0.007	1
MS-B86bl	0.333	13	0.333	13
MS2	0.059	4	0.046	4
average	0.153		0.121	

a The MAE and MSE (in eV) are computed with averaging over all 7 systems. The values of *r*_MAE_ and *r*_|MSE|_ rank the DFs according to best performance for the MAE and |MSE| error criteria, respectively.

**Figure 3:**
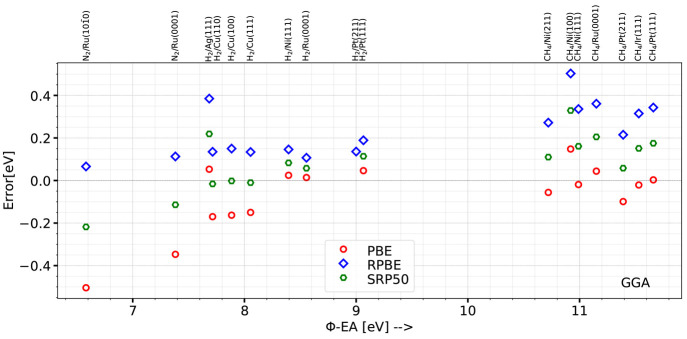
Correlation between the signed error and the difference of the work function of the metal surface and the electron affinity of the molecule for all the systems investigated. The results are for the high algorithm, for the GGA DFs tested.

**Figure 4:**
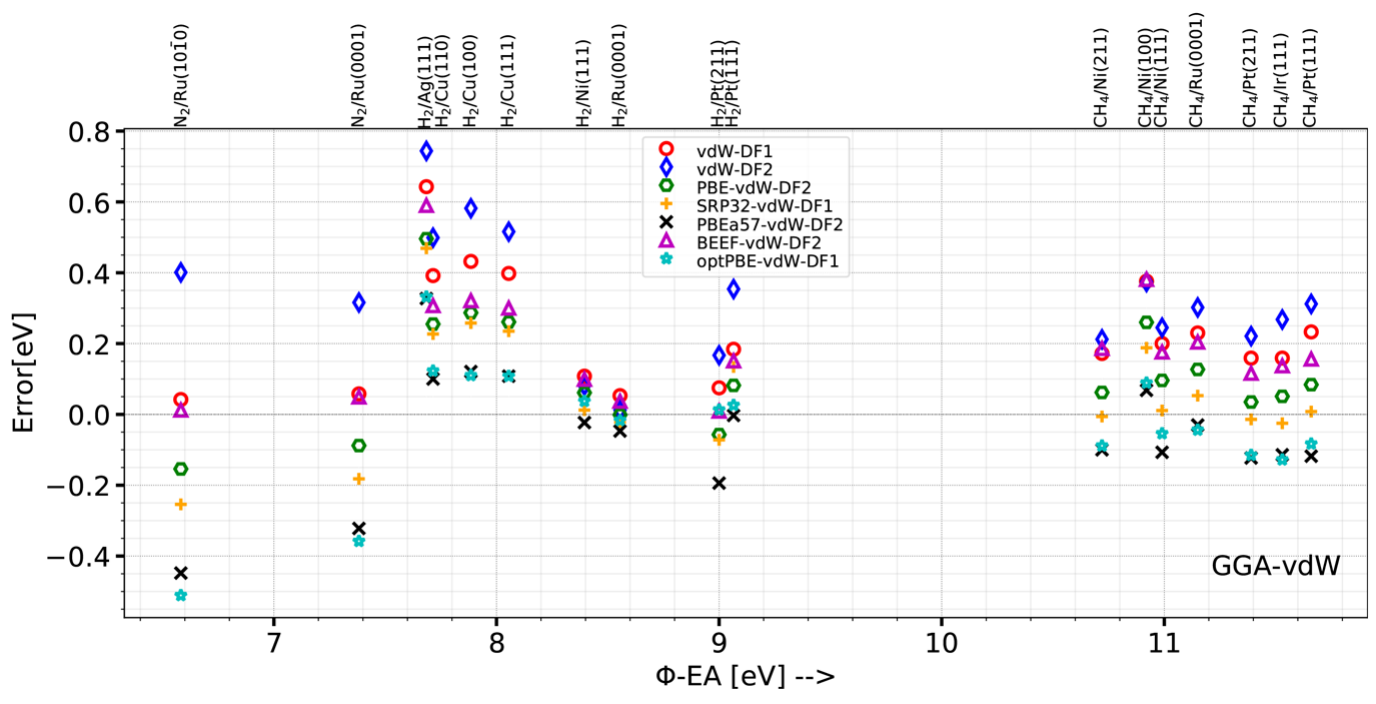
Correlation between the signed error and the difference of the work function of the metal surface and the electron affinity of the molecule for all the systems investigated. The results are for the high algorithm, for the GGA-vdW-DF1,2 DFs tested.

**Figure 5:**
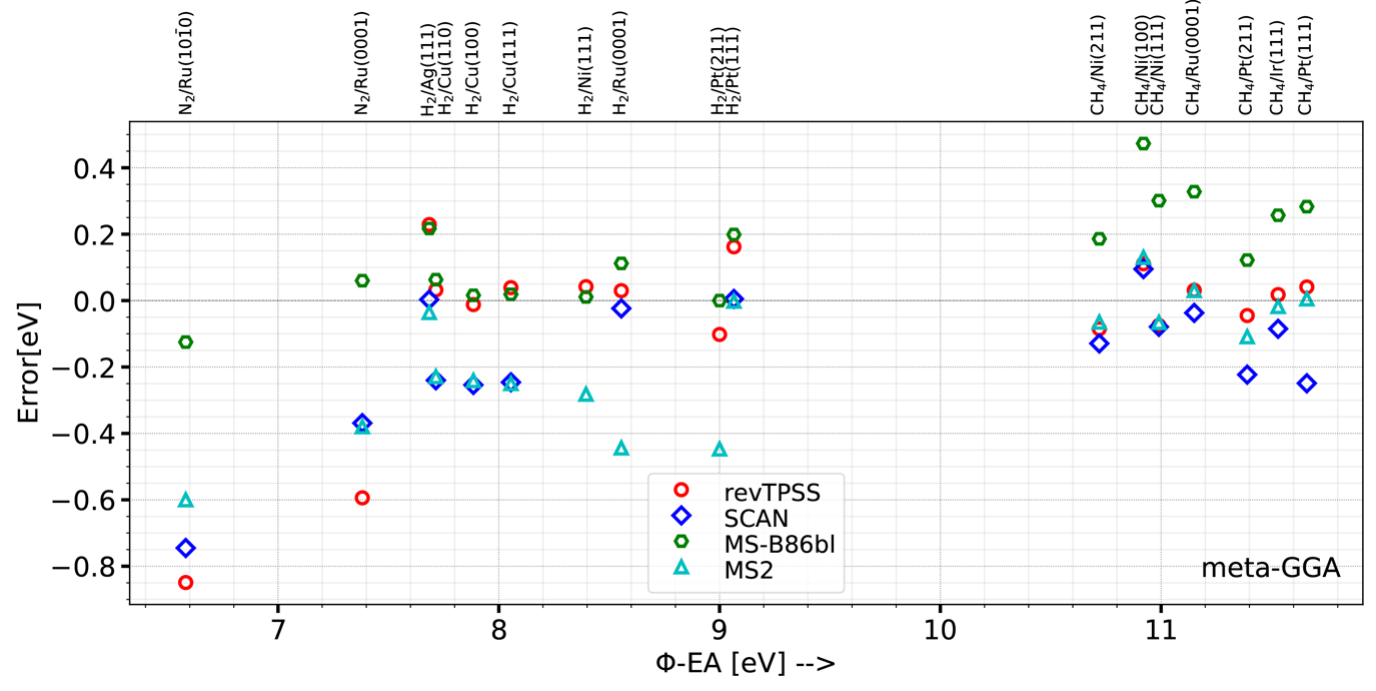
Correlation between the signed error and the difference of the work function of the metal surface and the electron affinity of the molecule for all the systems investigated. The results are for the high algorithm, for the meta-GGA DFs tested.

Table 12 shows the MAEs and the MSEs for the 17 systems investigated here, where now the averaging is done over the DFs. For both the medium and the high algorithms, the largest MAEs are found for the H_2_ + Ag(111), N_2_ + Ru(101̅0), and CH_4_ + Ni(100) systems. If results for these 3 systems are left out (leading to the database SBH14–3SBER, i.e, SBH17 with the 3 systems with the biggest errors removed), the MAEs and MSEs obtained with averaging over the systems now come out as shown in Table 13. As can be seen, omitting the systems for which the largest errors are made does not lead to large changes in the conclusions: according to the MSE criterion, revTPSS comes still out as best, followed by the same three DFs made up of GGA-exchange and nonlocal correlation (although now with a slightly different order) and PBE (see Tables 7 and 13). Omitting the three systems for which reference barrier heights came from an ad-hoc SE analysis rather than from SRP-DFT (resulting in the SBH14-SRP database) also does not yield large differences: the revTPSS and optPBE-vdW1 DFs still come out as the two best ranking DFs according to the |MSE| accuracy criterion (see Tables 7 and 13). Finally, the correlation of the signed error with (W-EA) is shown in Figures 3, 4, and 5 for the GGA-DFs, the DFs consisting of GGA exchange and vdW-DF1 or vdW-DF2 correlation, and the meta-GGAs tested here, respectively. A weak correlation seems to be present, with the GGA and meta-GGA DFs producing lower (higher) signed errors for systems with lower (higher) (W-EA).

**Table 12:** Overall Accuracy Achieved for Each System in the SBH17 Database with the Algorithms Tested^a^

	high algo		med algo		light algo	
system	MAE	MSE	MAE	MSE	MAE	MSE
H_2_/Cu111	0.205	0.104	0.197	0.098	0.245	0.133
H_2_/Cu100	0.218	0.115	0.209	0.118	0.240	0.101
H_2_/Cu110	0.205	0.104	0.165	0.120	0.286	0.171
H_2_/Ag111	0.339	0.334	0.335	0.330	0.380	0.375
H_2_/Pt211	0.126	–0.048	0.054	0.048	0.056	0.056
H_2_/Pt111	0.125	0.124	0.084	0.082	0.135	0.132
H_2_/Ru0001	0.074	–0.008	0.039	0.016	0.046	0.028
H_2_/Ni111	0.075	0.028	0.063	0.049	0.078	0.059
N_2_/Ru0001	0.230	–0.138	0.231	–0.141	0.318	–0.203
N_2_/Ru1010	0.340	–0.259	0.349	–0.275	0.400	–0.293
CH_4_/Ni100	0.264	0.264	0.266	0.266	0.270	0.270
CH_4_/Ni111	0.144	0.091	0.132	0.100	0.182	0.132
CH_4_/Ni211	0.126	0.058	0.120	0.090	0.134	0.045
CH_4_/Pt111	0.155	0.098	0.146	0.084	0.280	0.246
CH_4_/Pt211	0.118	0.024	0.117	0.068	0.164	–0.032
CH_4_/Ru0001	0.152	0.142	0.157	0.144	0.187	0.176
CH_4_/Ir111	0.124	0.084	0.131	0.094	0.239	0.221
average	0.177	0.065	0.164	0.076	0.214	0.095

a For a given system, mean absolute errors (MAEs) and mean signed errors (MSEs, both in eV) measure average deviations of the barrier heights computed using the DFs tested in this work from the reference values listed in Table 2. The averaging is done over the DFs, so that large deviations are likely to be indicative of inaccurate reference values.

**Table 13:** Density Functional Performance on Two Smaller Databases with 14 Barrier Heights in Them^a^

	database							
	SBH14-3SBER				SBH14-SRP			
density functional	MAE	*r*_MAE_	MSE	*r*_|MSE|_	MAE	*r*_MAE_	MSE	*r*_|MSE|_
PBE	0.074	1–2	–0.050	5	0.074	1	–0.050	4
SRP50	0.098	7	0.080	7	0.098	3	0.081	7
RPBE	0.208	13	0.208	13	0.210	12	0.210	12
vdW-DF1	0.191	11	0.191	11	0.219	13	0.219	13
vdW-DF2	0.267	14	0.267	14	0.294	14	0.294	14
PBE-vdW-DF2	0.107	8	0.093	9	0.128	9	0.115	9
SRP32-vdW-DF1	0.074	1–2	0.041	3	0.103	4–5	0.070	6
PBEα57-vdW-DF2	0.091	5	–0.045	4	0.112	7	–0.020	3
BEEF-vdW-DF2	0.163	10	0.163	10	0.189	11	0.189	11
optPBE-vdW-DF1	0.093	6	–0.033	2	0.114	8	–0.007	1
revTPSS	0.089	4	0.004	1	0.103	4–5	0.017	2
SCAN	0.108	9	–0.084	8	0.107	6	–0.082	8
MS-B86bl	0.196	12	0.196	12	0.186	10	0.186	10
MS2	0.086	3	–0.058	6	0.085	2	–0.064	5

a SBH14-3SBER was obtained from SBH17 by removing the 3 systems yielding the largest MAE when averaging over all DFs tested (see Table 12). SBH14-SRP only contains the 14 systems for which reference values of barrier heights were obtained from SRP-DFT. For each database, *r*_MAE_ and *r*_|MSE|_ rank the DFs according to best performance for the MAE and |MSE|criterion, respectively. All errors are in eV.

## IV Discussion

We should be clear that with the large amount of data here considered, a full analysis is beyond the scope of this paper. Instead, in our discussion below, we will focus on (i) the description of the metal and (ii) how well the different algorithms do for describing the barriers for the DC for the new database. Having determined an optimal algorithm, we then discuss (iii) how the different DFs perform overall for the new SBH17 database and (iv) how this depends on the three different types of systems in our database. Then, we (v) compare to new and old results for the earlier SBH10 database. We also (vi) compare to the performance of DFs with earlier results for molecular chemisorption and for gas phase reaction kinetics and thermochemistry. Finally, we also discuss future improvements and extensions of our database.

### IV.1 Description of the Metal

The trends in how accurately the tested DFs describe the lattice constants of the metals investigated here (Ag, Ir, Cu, Pt, Ni, and Ru), as revealed through Table 3, agree well with earlier work done on different sets of bulk solids. For instance, the RPBE DF is known to overestimate lattice constants more than the PBE DF,^16,163^ and it makes sense that the lattice constant computed with their 50/50 weighted average (SRP50) falls in between. It is also known that the vdW-DF1 and vdW-DF2 DFs substantially overestimate lattice constants, and much more so than PBE, but that the performance of optPBE-vdW-DF1 is similar to that of PBE, in agreement with Table 3.^16,50^ Our finding that BEEF-vdW-DF2 performs somewhat worse than optPBE-vdW-DF1 is likewise in agreement with earlier findings,^16^ and the same is true for the earlier finding that PBEα57-vdW-DF2 and optPBE-vdDF1 perform similarly for lattice constants.^98^ The SRP32-vdW-DF1 and PBE-vdW-DF2 DFs, which to our knowledge have not been widely tested on solids yet, show a performance that is just a little better than that of vdW-DF1 and vdW-DF2.

Our finding that the four meta-GGA DFs tested here are better for lattice constants than PBE is likewise in agreement with earlier work. This has been confirmed in refs (16 and 163) for revTPSS and in refs (163 and 164) for SCAN. Tran et al.^163^ found a similarly good performance for MS2 as for revTPSS and SCAN, in agreement with Table 3. Finally, like MS2^41^ the MS-B86bl^55^ was developed to perform like the PBEsol^48^ GGA for metals, and its resulting good performance for metals is in agreement with earlier findings.^98^

Interlayer distances computed with the tested DFs (Table 4) are not always in good agreement with experimental values and with literature values obtained with the same DFs. This is not any reason for concern: converging the values of interlayer distances requires thicker slabs (a larger number of layers, of the order of eight or more^144,165^) than needed for converging reaction barrier heights (typically 4 or 5). As the focus in this work is on reaction barrier heights, no attempts were made to compute interlayer distances that were converged with slab thickness.

### IV.2 Description of Barrier Heights to the DC

#### IV.2.1 Preferred Algorithm

Table 6 can be used to select the optimal algorithm for testing DFs on reaction barrier heights for the DC. In selecting this algorithm, we also take into account that, for a typical system, the high algorithm requires more “human time” and roughly an order of magnitude more CPU time than the medium algorithm, due to the need to find the saddle point geometry corresponding to the DF tested and the system described. The light algorithm requires even less “human time” than the medium algorithm, as the lattice constant(s) of the metal and the geometry of the metal slab representing the surface also do not need to be optimized for each metal and metal surface, respectively. However, the light algorithm is not much less CPU-intensive than the medium algorithm.

Table 6 suggests the use of the medium algorithm for the following two reasons. The first reason is that for all GGA DFs, for all DFs combining GGA exchange with nonlocal correlation, and for revTPSS the medium algorithm leads to results that hardly differ from the results of the much more expensive high algorithm. In contrast, the light algorithm leads to results that differ considerably from those of the medium algorithm, i.e., higher MAEs and MSEs. This result suggests that, at least for now and while DFs are developed that yield a simultaneously good description of interaction energies and metal structure, the medium algorithm should be used. Figure 2 suggests an explanation: for GGA DFs, and apparently also for the DFs combining GGA exchange with nonlocal correlation, the predicted barrier height and metal lattice constant are correlated, with higher barriers corresponding to larger lattice constants, which has been known for some time.^48,49^ Apparently reaction barrier heights are then best computed with the metal surface appropriately relaxed with the DF tested (as done in the high and medium algorithms), which may be related to the observation that reaction barrier heights may be strongly affected by lattice strain.^147^ We note that the problem that with GGA DFs barrier heights are usually correctly predicted at the cost of overestimated lattice constants may in principle be solved by resorting to a meta-GGA DF, as the use of the kinetic energy density allows the DF to distinguish between metallic and covalent bonding.^166^ This should also explain why the correlation observed in the upper two panels of Figure 2 between lattice constant and barrier height is not observed for the meta-GGA DFs.

The second reason to use the medium algorithm is simply that it produces the lowest averaged MAE when the MAEs of the barrier heights are averaged over all DFs tested (Table 6). The simplest explanation being that the medium algorithm allows the best description of the reaction barrier height; Occam’s razor then suggests the use of the medium algorithm. From now on, our discussion will therefore focus on results obtained with the medium algorithm.

#### IV.2.2 Performance of DFs for SBH17 with the Medium Algorithm

If we take the MSE as the accuracy criterion, of the DFs tested the revTPSS meta-GGA comes out as best with an MSE of 25 meV, which corresponds to 0.58 kcal/mol (see also Table 7). Of the five best performing DFs, three are made of GGA exchange and nonlocal correlation, and the DF ranked fifth is the PBE GGA DF. Both the revTPSS and PBE DFs may be described as nonempirical, constraint-based DFs, and interestingly, both have been cast as general purpose, workhorse functionals.

The MAE is probably the best accuracy criterion, as this quantity tells us by how much the barrier height we compute with a given DF will typically be off from the real value. According to this criterion, the PBE DF comes out best, with an MAE of 0.103 eV (2.4 kcal/mol). With this criterion, revTPSS comes out as ninth, with an MAE of 0.146 eV (3.4 kcal/mol). The MS2 DF now comes out as the best meta-GGA DF (MAE = 0.117 eV = 2.7 kcal/mol). The highest ranked GGA+vdW DF now is SRP32-vdW-DF1, which has a second overall ranking (MAE = 0.115 eV = 2.7 kcal/mol).

The major conclusions regarding the accuracy of DFs for the type of DC reactions on SBH17 are robust in the sense that if we remove the three systems from the database that lead to the largest errors (leading to the SBH14–3SBER database) the order of the best performing DFs remains more or less the same. As Table 13 shows, revTPSS is still the best in terms of MSEs, and PBE still ranks first in terms of MAEs (although now together with SRP32-vdW-DF2). The best five performing DFs in terms of MSEs and the best three in terms of MAEs remain the same (compare Tables 7 and 13).

The major conclusions regarding DF accuracy also remain unchanged if we use the SBH14-SRP database instead of the SBH17 database (compare Tables 7 and 13). For instance, the PBE DF remains the best performing DF according to the MAE criterion. SRP32-vdW-DF1 ranks second according to this criterion for SBH17 and still fourth (together with revTPSS) for SBH14-SRP; MS2 ranks third for SBH17 and second for SBH14-SRP. Removing the three systems for which reference barrier heights were obtained using an ad-hoc SE approach does lead to considerably smaller absolute values of the MAE, e.g. 74 meV (1.7 kcal/mol) for PBE under SBH14-SRP vs 103 meV (2.4 kcal/mol) under SBH17. This suggests that the conclusions regarding DF performance on DC barrier heights in SBH17 would be even more favorable than now obtained if the reference values for the three systems discussed were to be replaced with more accurate SRP-DFT values. The following two observations provide additional evidence that the reference values for at least two of the three systems left out in SBH14-SRP are inaccurate: (i) the SRP32-vdW-DF1 functional, which performs so well for CH_4_ + metal surface systems, shows a comparatively poor performance on CH_4_ + Ni(100) (Table 11 and Figure 4), and (ii) the PBE DF, which shows the lowest MAE for SBH17, shows a larger error on the N_2_ + Ru(101̅0) system than on any other system (Figure 3).

If we compare trends found for barriers for the DC on metals to trends found for gas phase reaction barriers, a number of important differences stand out. First of all, the MAEs tend to be smaller for DC barriers than for gas phase reaction barriers. To give an example, the MAE of the PBE DF for the BH76 database for hydrogen atom transfer and non-hydrogen atom transfer reactions is 8.9 kcal/mol,^26^ while the MAE found here is 2.4 kcal/mol. It is important to note that this difference does not arise from the barrier heights being much larger for the BH76 database: the average over the absolute values of the barrier heights is 18.6 kcal/mol for BH76,^23^ which is not much smaller than for SBH17 (14.8 kcal/mol). Second, while RPBE clearly outperforms PBE for gas phase reactions,^24,26,167^ the opposite is the case for the DC barriers we consider here. Third, and most importantly, while the PBE and RPBE DFs both systematically underestimate gas phase reaction barrier heights,^167^ here we find that the RPBE DF systematically overestimates reaction barrier heights, while the PBE DF neither systematically underestimates nor systematically overestimates DC barriers for the systems we consider. We consider this last point a key point, which should be a telltale concerning semilocal DFT and fundamental differences between gas phase reactions and the DC on metals. For this, we note that the deficiency of semilocal DFT for gas phase reactions has often been rationalized in terms of the delocalization error of Yang and co-workers.^168−170^ The following hand waving explanation has been put forward for explaining the comparatively good performance of semilocal DFT for DC barriers in the systems in the database:^13^ of the electrons responsible for the formation of bonds between the molecular fragments and the surface, the ones coming from the molecule become more delocalized in the transition state, while the opposite is true for the electrons coming from the metal, which are quite delocalized to start with. This leads to error cancellation. A weakness of this explanation is that it is hard to see how it can be tested or falsified, and more research is needed to clarify the origin of the differences between the performance of semilocal DFT for reaction kinetics in the gas phase and on metal surfaces.

Considering specific DFs, we note that, as found in other studies of molecules interacting with metal surfaces,^55,171^ the maximally constrained meta-GGA DF SCAN does not outperform the PBE GGA DF for DC barriers, showing a similar performance to the revTPSS DF for the MAE. The somewhat weak performance of SCAN for adsorption of molecules on metal surfaces has been attributed to density driven errors.^171^ The MS2 meta-GGA DF performs reasonably well for DC barriers, ranking third according to the MAE criterion, with an MAE of 0.117 eV (2.7 kcal/mol). The MS86bl DF, which has been constructed in such a way that its performance should be biased in favor of systems containing hydrogen,^55^ is the meta-GGA DF performing least well for DC barriers here.

Of the DFs built from GGA exchange and nonlocal correlation, the optPBE-vdW-DF1, the SRP32-vdW-DF1, and the PBEα57-vdW-DF2 DFs perform quite well here, ranking among the best 4 according to the MSE and among the best 6 according to the MAE criterion. For the SRP32-vdW-DF1 and the PBEα57-vdW-DF2 DFs, this is not so surprising as they are known to be SRP-DFs for some of the systems in our database (see Table 2). However, the optPBE-vdW-DF1 DF was first developed to obtain an improved description of weak interactions,^99^ and only later was this DF shown to accurately model systems in which H_2_ interacts with copper surfaces.^88,101^ The original vdW-DF1 and vdW-DF2 DFs do not exhibit a very good performance for the DC, ranking 12^*th*^ and 14^*th*^ on both accuracy criteria. PBE-vdW-DF2 exhibits a reasonable performance. The performance of BEEF-vdW-DF2 would seem to be disappointing as well, as it seemed to perform much better in the earlier tests on the SBH10 database.^40^ This issue will be further considered below.

#### IV.2.3 Dependence on the Type of System

The performance of the tested DFs on H_2_-metal systems (Table 9) does not contain great surprises. The SRP50 DF performs better on this subdatabase than on SBH17, but this is no great surprise as this DF is close to the SRP48 DF, which is an SRP-DF for H_2_ + Cu(111).^96^ The SRP32-vdW-DF is also less good for the H_2_-metal subdatabase than for SBH17, which may be explained from this DF being an SRP-DF for several CH_4_ + metal systems, while it performs poorly for the DC of H_2_ on Cu and Ag surfaces (see Table 2 and Figure 4).

The performance of the tested DFs on N_2_-metal systems (Table 10) is rather different from that on the SBH17 database. Specifically, the best four performing DFs for N_2_-metal systems (MS-B86bl, BEEF-vdW-DF2, vdW-DF1, and RPBE according to both the MSE and MAE criteria) show a rather poor overall performance on SBH17. The origin of this discrepancy is not entirely clear. However, there appears to be a weak correlation between the MSE of a given functional and (W-EA) (see Figures 3-5). A trend that may be discerned is that the MSE increases with the (W-EA). The N_2_-metal systems have a low (W-EA) and lie on one of the outer edges of the range of (W-EA) spanned by the systems investigated here (see Figures 3-5). These two observations together perhaps explain why the DFs that come out best for N_2_-metal systems do not do well for SBH17 as a whole: for many of the systems in the database with higher (W-EA), these DFs will produce much higher unsigned errors.

Finally, coming to the CH_4_-metal systems (Table 11), the only real surprise is that SCAN performs quite well for these systems. The good performance of SCAN for systems with high (W-EA) (see Figures 3-5) is consistent with the explanation that for this DF errors in molecule-metal surface interactions are density driven: for the methane-metal systems, little if no electron transfer will occur from the metal surface to the molecule. This would suggest that errors associated with electron delocalization and self-interaction should be small,^56^ which would in turn suggest that density driven errors should be small.

#### IV.2.4 Comparison to Present and Previous Results for SBH10

To allow a better comparison between the results for the present SBH17 and the older SBH10 database, in Table 14, we compare the MAEs obtained for both databases for the 9 DFs that performed best for SBH17 according to the MAE accuracy criterion. In Table 14, we also show how these DFs ranked according to both the MAE and the MSE accuracy criterion in both databases.

**Table 14:** Comparison of DF Performance on the SBH17 and SBH10 Databases^a^

DF	*r*_MAE_SBH17	MAE SBH17	*r*_MAE_SBH10	MAE SBH10	*r*_|MSE|_
PBE	1	0.103	5	0.143	5 (5)
SRP32-vdW-DF1	2	0.115	1	0.126	4 (1)
MS2	3	0.117	8	0.171	6 (7)
PBEα57-vdW-DF2	4	0.124	2	0.132	3 (3)
SRP50	5	0.125	3	0.133	7 (2)
optPBE-vdW-DF1	6	0.131	4	0.141	2 (4)
SCAN	7	0.140	7	0.179	8 (10)
PBE-vdW-DF2	8	0.141	6	0.148	9 (6)
revTPSS	9	0.146	9	0.177	1 (7)

a For the nine DFs that performed best for SBH17 according to the MAE (eV) criterion, a comparison is made with their performance for the SBH10 database. For this, the rank *r*_MAE_ of the DF is presented according to the MAE (eV) criterion for both SBH17 and SBH10, as well as the MAE (eV) for the DF for each database. The last column lists the ranks *r*_|MSE|_ according to the MSE (eV) as accuracy criterion, for both SBH17 and SBH10 (with *r*_|MSE|_ for the latter given in parentheses). All results are for the medium algorithm.

The comparison shows that, on the whole, not much changes when comparing our new results for SBH10 to our new results for SBH17. Only in one case is the MSE changed by more than 1 kcal/mol (∼43 meV), i.e., for the meta-GGA MS2 functional (by 54 meV). The second largest change occurred for the GGA PBE DF (40 meV), and the third largest change occurred for the meta-GGA revTPSS DF (by 31 meV). In all three cases, the MAE is increased going from SBH17 to SBH10. Inspection of Figures 3 and 5 suggests that for these 3 DFs the discrepancy could to a large extent be due to the larger weight of the N_2_-metal systems in the SBH10 database (20%) compared to that in the SBH17 database (12%), as the three DFs mentioned all perform rather poorly for the systems containing N_2_.

Finally, there is the matter of how the old results for SBH10^40^ compare to the new results for SBH10 and for SBH17. The old study compared results for three DFs where each is a representative of a specific class of DFs, i.e., rung 2 (GGA) exchange with vdW-DF2 correlation (BEEF-vdW-DF2), rung 3 exchange and rung 3 correlation (MS2), and a rung 4, screened hybrid DF (HSE06).^42^ With the latter DF, only results were obtained for the H_2_ metal systems. For this reason, and because we did not test any rung 4 DFs here, we will not discuss the old HSE06 results here.

**Figure 6:**
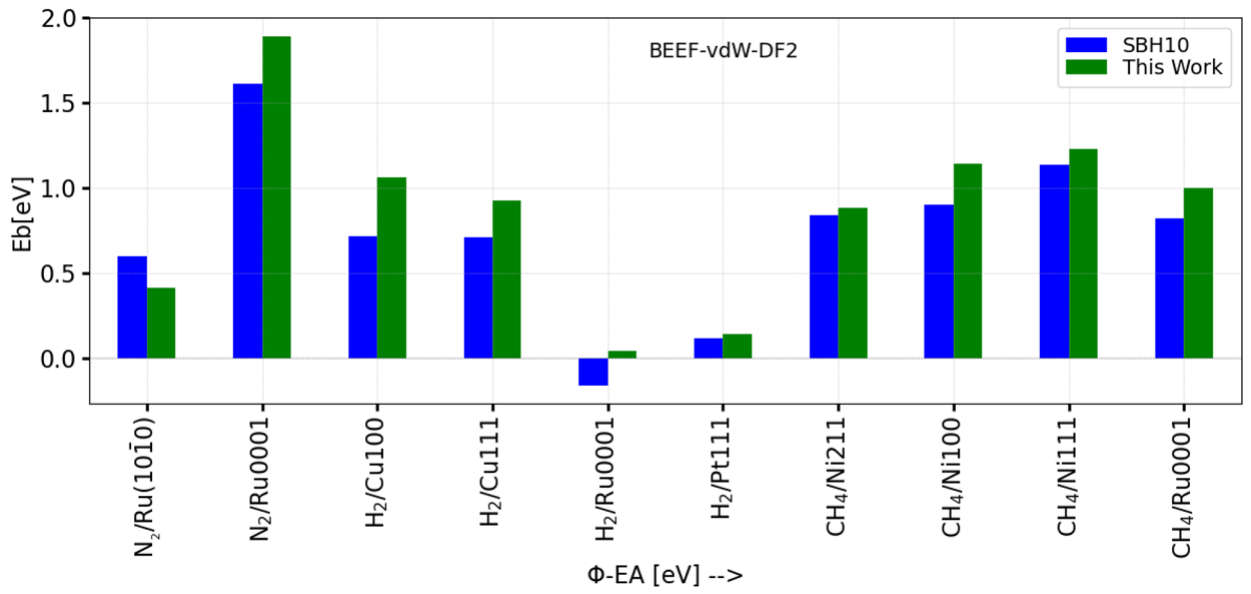
Comparison of barrier heights computed with the BEEF-vdW-DF2 DF for the systems in the SBH10 database, allowing the surface to relax in the TS (SBH10, results from ref (40)) and using the medium algorithm, in which the surface is held fixed at the metal-vacuum interface geometry (this work).

First, comparing the old SBH10 to the new SBH10 results here (see Table 8), fairly large differences are noted for the two DFs tested. The old results showed a somewhat better performance for the BEEF-vdW-DF2 DF (MAE, MSE = 0.12, 0.03 eV) than here obtained (MAE, MSE = 0.18, 0.18 eV for the medium algorithm, see also Table 8). On the other hand, the old results showed a considerably worse performance for the MS2 DF (MAE, MSE = 0.36, −0.34 eV) than here obtained (MAE, MSE = 0.17, −0.12 eV for the medium algorithm, see also Table 6). The explanation for this difference is as follows. A shortcoming of the method to compute barrier heights in the older work was that the metal surface was allowed to relax in the presence of the molecule for 9 of the 10 systems in the database in the calculation of the transition state energy. From a physical point of view, this is incorrect when interpreting the outcome of supersonic molecular beam experiments, where the molecule comes in fast and the surface atoms do not have time to respond to its presence.^10^ Using this incorrect procedure should lead to an underestimate of the classical barrier height relative to SRP-DFT or experimentally estimated values obtained from supersonic molecular beam sticking experiments, which should reflect the situation where the surface atoms have not relaxed in response to the incoming molecule. How this affects the results for a given DF depends on its MSE. The BEEF-vdW-DF2 DF has a small positive MSE for SBH10 with the old algorithm, which should then go up with the new algorithm, as should the MAE. This explains the worse performance of BEEF-vdW-DF2 for SBH10 with the newer and better algorithm (As Figure 6 shows, barrier heights increase with the new algorithm, the reason being that the TS energy comes out higher because the surface is not allowed to relax.). The MS2 DF has a large negative MSE for SBH10 with the old algorithm, which should then become smaller but still negative with the new algorithm, and this should lead to a smaller MAE, as indeed observed.

We now compare the old SBH10 results to the new SBH17 results. The old results showed a somewhat better performance for the BEEF-vdW-DF2 DF (MAE, MSE = 0.12, 0.03 eV) than here obtained for SBH17 (MAE, MSE = 0.19, 0.19 eV, medium algorithm, see also Table 7). On the other hand, the old SBH10 results showed a considerably worse performance for the MS2 DF (MAE, MSE = 0.36, −0.34 eV) than here obtained for SBH17 (MAE, MSE = 0.12, −0.07 eV, medium algorithm, see also Table 7). In contrast to the older SBH10 work, we thus find a better performance of the MS2 DF than of the BEEF-vdW-DF2 DF. However, this better performance could in principle reflect the smaller proportion of N_2_-metal systems in SBH17 than in SBH10. If it turns out that, as discussed above in Section IV.2.2, MS2 also systematically underestimates barrier heights for N_2_-metal systems, then the performance of this DF for a more balanced database (which should contain more N_2_-metal systems relative to H_2_- and CH_4_-metal systems than now is the case) could be somewhat worse than now found. However, our results do not support the conclusion that might be drawn from the older SBH10 work that meta-GGA functionals systematically underestimate reaction barrier heights for the DC on metals: this is not true for revTPSS (MSE = −25 meV), for MS2, and even for SCAN (MAE = 140 meV, MSE = −105 meV, see Table 7), and it is certainly not true for MS-B86bl (MSE = 195 meV). Our new study also does not support the idea that meta-GGA DFs should be worse for the DC on metals than GGA DFs.

### IV.3 Comparison to Results for Adsorption and to Gas Phase Results

In Table 15, our new results for SBH17 are compared to results for adsorption of molecules to metal surfaces, focusing on strong molecule-metal surface interactions, i.e., on chemisorption. The data we compare to come from calculations on the CE26 database^18^ and from calculations on the CE21b database,^172^ where the latter may be viewed as a subdatabase of the former. We use the MAE (or, if not available, the RMSE) as the accuracy criterion, and the DFs are listed in order of increasing RMSE for the CE26 database. The most important observation that can be made is that the DFs that perform best for DC barrier heights (a kinetic property) usually are not best for chemisorption energies (a thermochemical property) and vice versa. To give a few examples: PBE performs best for DC barriers in SBH17 but ranks sixth of the DFs listed in Table 15 for chemisorption energies. Similarly, the three best DFs for chemisorption (BEEF-vdW-DF2, vdW-DF1, and RPBE) did not perform particularly well for dissociation barriers, ranking 10^*th*^, 12^*th*^, and 13^*th*^ among the 14 DFs tested on SBH17. A DF performing reasonably well on both chemisorption and the DC is MS2, which ranks 4^*th*^ for chemisorption and 3^*rd*^ for DC barriers in Table 15, and may be said to yield the best overall performance on molecule-metal surface interactions. On the basis of the results in Table 15 we do not agree with the statement that “a functional that predicts chemisorption energies accurately can also predict barrier heights with comparable accuracy”.^18^ In ref (18), this conclusion referred to the BEEF-vdW-DF2, which performs well for chemisorption. However, as shown here, its performance for barrier heights is not particularly good if the metal surface is treated appropriately (see Section IV.2.2), which was not the case in ref (18).

**Table 15:** DF Performance for Kinetics and Thermochemistry of Molecules Reacting with Metal Surfaces^b^

		database					
		CE21b		CE26		SBH17	
DF	type DF	MAE	MSE	RMSE	MSE	MAE	rank
BEEF-vdW-DF2	GGA+vdW			0.21	0.0	0.19	10
vdW-DF1	GGA+vdW			0.21	0.09	0.22	12
RPBE	GGA	0.15	0.07	0.23	0.09	0.23	13
MS2	meta-GGA			0.27	–0.15	0.12	3
vdW-DF2	GGA+vdW			0.29	0.15	0.31	14
PBE	GGA	0.30	–0.28	0.31	–0.19	0.10	1
revTPSS	meta-GGA	0.30	–0.28	0.31^a^		0.15	9
SCAN	meta-GGA	0.47	–0.46	0.45	–0.39	0.14	7
optPBE-vdW-DF1	GGA+vdW			0.54	–0.42	0.13	6

a Inferred from PBE value for CE26 and similar performance of PBE and revTPSS on the MAE in CE21b.

b Errors for adsorption energies as present in the CE21b^172^ and CE26^18^ databases are compared to MAEs computed for DC barriers for the new SBH17 database, for the DFs for which results were provided in the chemisorption databases. All errors are in eV.

In Table 16, kinetic data coming from barrier height databases (the present SBH17 results for surface reactions and BH76 and BH206 for gas phase reactions) and thermochemical data (the CE26 results for chemisorption at metal surfaces and AE6 for atomization energies and TCE for “easy” thermochemical gas phase interactions) are compared for a selection of the GGA and meta-GGA DFs tested here. We see that some of the observations for surface reactions also hold for gas phase interactions. For example, the functional of PBE and RPBE that is best for gas phase reaction barriers (RPBE in BH76 and BH206) is not necessarily best for gas phase thermochemistry (with RPBE outperformed by PBE for the large TCE database, although not for the small AE6 database). For the databases listed in Table 16, MS2 has the best overall performance. A striking observation is that RPBE is good for chemisorption (for which it was optimized^39^), while PBE is good for DC barrier heights (for which it was not optimized), as already noted above. In Section IV.2, the point that RPBE is better than PBE for gas phase reactions but not for metal surface reactions was already discussed. The revTPSS DF exhibits a fairly robust performance for all the databases in Table 16. SCAN is robust for the gas phase databases, poor for chemisorption, but rather good for DC barriers.

**Table 16:** DF Performance for Kinetics and Thermochemistry of Molecules Reacting with Metal Surfaces and for Gas Phase Chemistry^a^

		database					
		SBH17	BH76	BH206	CE26	AE6	TCE
DF	type DF	MAE	MAE	RMSE	RSME	MAE	RMSE
PBE	GGA	0.10	0.43	0.40	0.31	1.02	0.40
MS2	meta-GGA	0.12	0.27^203^	0.27	0.27	0.19^204^	0.29
SCAN	meta-GGA	0.14	0.34^54^	0.33	0.45	0.15	0.23
revTPSS	meta-GGA	0.15	0.35	0.32	0.31	0.28	0.27
RPBE	GGA	0.23	0.34	0.33	0.23	0.42	0.42

a Comparison of performance of a selection of GGA and meta-GGA DFs for gas phase and metal-surface interactions. Unless indicated otherwise with explicit references the data come from the present results for the SBH17 database (this work) and works presenting data for the BH76 database,^26^ the BH206 database,^24^ the CE26 database,^18^ the AE6 database,^172^ and the TCE database.^24^ All errors are in eV.

### IV.4 Future Improvements

On the basis of the above, we see the following possible improvements of the present database for DC barriers on metals and for testing DFs on the database.

First, we suggest that in the future the entries in the database are as much as possible based on SRP-DFT and not on more ad-hoc SE procedures. This would require dynamics calculations with trial DFs on CH_4_ + Ru(0001) and CH_4_ + Ni(100), for which molecular beam experiments are already available,^122,127^ and new experiments and dynamics calculations on N_2_ + Ru(101̅0), for which molecular beam sticking experiments are, to our knowledge, not yet available. As noted above, our comparison between MAEs computed with PBE for SBH17 and SBH14-SRP suggests that replacing the reference values with SRP-DFT values for the three systems mentioned is likely to lead to smaller MAEs for a thus improved version of the SBH17 database. Second, we suggest that the database be extended with additional N_2_-metal systems. It may be possible to do this by semiempirically fitting SRP-DFs to supersonic molecular beam sticking data on N_2_ + Fe(111),^173,174^ W(110),^175,176^ and W(100).^176−179^ Adding these data is desirable to make the database more balanced, as it is now dominated by data for the DC of H_2_ and CH_4_ on metal surfaces. Also, it would show whether our results for the MS2 DF are robust to addition of more N_2_-metal systems to the database, for which this DF did not perform so well, and the same holds for the optPBE-vdW-DF1 and PBE DFs.

On the longer term, it should be necessary to extend the database with systems for which the charge transfer energy, which equals (W-EA), is less than 7 eV. As noted in ref (56), DFs with semilocal exchange would appear to systematically overestimate the reactivity of such systems, suggesting that DFs with screened exact exchange are required for a good description. Examples of systems for which molecular beam sticking data are available include e.g. H_2_O + Ni(111),^180^ HCl + Au(111),^181^ O_2_ + Al(111),^182,183^ Ag(110),^184,185^ Cu(100),^186^ and Cu(111).^187^ Inclusion of such systems in the database would certainly alter the view of the performance of DFs for the DC on metal surfaces, where the view offered in the present work is specific to systems with (W-EA) > 7 eV, the only exception being N_2_ + Ru(101̅0).

Finally, of course, a far larger number of DFs exist than here tested. While we could mention specific DFs here that would be nice to test, this might not do justice to others, as several DFs exist (see e.g. the DFs tested in refs (23, 24, and 26)). However, a particular DF we would like to mention is the new machine learned DF DM21.^188^ Even though this DF has not been trained on interactions involving transition metals, it would be good to see how it performs on SBH17. It would also be good to test recently developed functionals combining screened exact exchange with vdW-DF1 and vdW-DF2 correlation,^189,190^ which may work especially well for the representative database we envisage. We advocate that such future benchmark tests would also incorporate calculations employing the CE26 database for chemisorption on metals.^18^

Last but not least, it would also be good to mention something we would like to keep the same for now. A nice conclusion from the present work is that benchmarking of DFs on the SBH17 database can be done with the “medium algorithm”. While this requires some additional work to what is needed for benchmarking DFs on kinetic and thermochemical data on chemical reactions, the overall extra effort required (of determining the lattice constant of the 6 metals present in the database for each DF and the interlayer relaxation of the metal slabs of the 12 different metal surfaces used here) is manageable. For this reason, we also hope that others will start using the SBH17 database and that it will be incorporated in the larger databases that are now used for extensive benchmarks of gas phase reactions,^23,24,26^ which unfortunately do not yet include data for reactions on metal surfaces.

## V Conclusions and Outlook

We have presented a new database with barrier heights for the DC on metal surfaces that can be used for benchmarking electronic structure methods. The new database is called SBH17 and contains barriers for 17 systems, including 8 H_2_ metal systems, 2 N_2_ metal systems, and 7 CH_4_ metal systems. For 16 systems, (W-EA) exceeds 7 eV. The barrier heights come from SRP-DFT (14 systems) and from more ad-hoc SE procedures (3 systems). The new database is meant to replace an older database (SBH10) that contained barriers for 10 of the 17 systems now treated.

We have tested 14 DFs on the new database, of which three were GGA DFs, 4 meta-GGA DFs, and 7 DFs containing GGA exchange and vdW-DF1 or vdW-DF2 nonlocal correlation. We first tested how the performance of these DFs depends on the algorithm or procedure used. Three different algorithms were tested, which were labeled “high”, “medium”, and “light” according to the investment of computer time that was required for the calculation. In the algorithm that is the best compromise between accuracy and invested computer time (the medium algorithm), for each DF tested, one computes the lattice constant of the metals in the database. Next, for each DF tested, for each metal surface in the database one performs a relaxation of the interlayer distances between the top layers. Then, for each system in the database and for each DF, the barrier height is computed on the basis of two single point calculations. One of these calculations is for a geometry where the molecule is in the gas phase, and one is for a geometry where the molecule is in the saddle-point geometry with respect to the surface obtained from the previous calculations. This saddle point geometry is either the one previously obtained from an SRP-DFT calculation (if the barrier height comes from SRP-DFT) or from a calculation with a functional that is expected to perform best (if the barrier height is a guess based on experimental data).

Of the DFs tested, the meta-GGA DFs perform best at describing the metal, followed by PBE and optPBE-DF1. When the MAE is taken as the accuracy criterion, the workhorse PBE GGA DF performs best on the SBH17 database, with an MAE of 2.4 kcal/mol. Other top performers are the MS2 meta-GGA functional and two functionals consisting of GGA exchange and nonlocal correlation (SRP32-vdW-DF1 and PBEα57-vdW-DF2). Surprisingly, none of the DFs tested systematically underestimates reaction barriers for the DC on metals, in contrast to findings for gas phase reactions. This finding should be a telltale on the origin of flaws of semilocal DFs for gas phase reaction barriers and differences between gas phase reactions and DC reactions on metals, suggesting further research on these topics.

Our results for the accuracy of the DFs for DC barriers are robust to the extent that their ranking according to MAE is rather insensitive to removing the three systems yielding the biggest errors in the database, to removing the three systems for which reference barrier heights were obtained with an ad-hoc SE analysis, and to applying the functionals to the older SBH10 database. Improving SBH17 by ensuring that all reference barrier heights come from SRP-DFT is likely to reduce the MAEs of the best performing functionals considerably, e.g. to an error less than 2 kcal/mol for PBE. We obtain different results regarding the relative accuracy of the MS2 and BEEF-vdW-DF2 functionals than obtained in an earlier study of the SBH10 database, which we attribute to an incorrect treatment of the surface atoms in the transition states in the earlier study.

For the subdatabases with H_2_-metal systems, N_2_-metal systems, and CH_4_-metal systems, rankings are obtained that differ from the overall ranking for the complete database. The SRP50-DF (the 50/50 mixture of the PBE and RPBE GGA DFs) performs best for H_2_-metal systems. BEEF-vdW-DF2 performs best for N_2_-metal systems, and SRP32-vdW-DF1 performs best for CH_4_-metal systems.

The DFs performing best for DC barriers (i.e., kinetics) are not the ones that perform best for databases (CE26, CE21b) of chemisorption energies on metals (i.e., thermochemistry). This trend is paralleled in the performance of DFs on databases for kinetics (BH76, BH206) and thermochemistry (AE6, TCE) in the gas phase. The meta-GGA MS2 DF is the functional with the best overall performance for DC barriers and chemisorption energies on metals. Of the five GGA and meta-GGA DFs considered for their performance on 6 databases for kinetics and thermochemistry on metal surfaces and in the gas phase (PBE, RPBE, revTPSS, MS2, and SCAN), again MS2 showed the best overall performance.

Future improvements of the present database include replacing estimates of barrier heights from ad-hoc SE procedures with SRP-DFT values, adding data for the underrepresented N_2_-metal systems, and extending the databases with systems for which (W-EA) is less than 7 eV. Chemically accurate barriers for the latter category of systems do not yet exist, and obtaining them may require a fundamentally different approach than the SE SRP-DFT approach forming the basis of the present database. Adding such systems should be important because they include systems relevant to sustainable chemistry (e.g., oxygen containing molecules like water and methanol), and because conclusions regarding the performance of DFs for the more general database also including such systems might be different from the present conclusions. In spite of the present limitations of the database, we hope that the new database finds its way into benchmark tests of new and already existing DFs, as it is rather odd that such tests do not yet include the type of reactions that arguably is most important for producing chemicals.

## Supporting Information Available

Pseudopotential convergence tests for N_2_ + Ru(0001) (Table S1), CH_4_ convergence tests for each DF (Table S2), details of parameter setup for all calculations (Table S3), barrier heights for other systems in database other than H_2_ + Cu(111) (Tables S4–S19 and Figures S1–S16), and bar plots of MAE and MSE for H_2_-metal systems (Figures S17 and S18), for N_2_-metal systems (Figures S19 and S20), and for CH_4_-metal systems (Figures S21 and S22) (PDF)
Transition state and gas phase geometries input for medium algorithm, for VASP calculations with PBE DF for each system (ZIP)
