# Supplementary material for: SBH17: Benchmark Database of Barrier Heights for Dissociative Chemisorption on Transition Metal Surfaces
Source: J Chem Theory Comput. 2022 Dec 19;19(1):245–70. doi: 10.1021/acs.jctc.2c00824 (PMC9835835; doi:10.1021/acs.jctc.2c00824)
Supplement: Supplementary file 1 — ct2c00824_si_001.pdf [file ct2c00824_si_001.pdf]

# Supporting information for : SBH17:Benchmark Database of Barrier Heights for Dissociative Chemisorption on Transition Metal Surfaces.


T. Tchakoua,† N. Gerrits,†,‡ E. W. F. Smeets,†,¶ and G.-J. Kroes*,†

†*Leiden Institute of Chemistry, Gorlaeus Laboratories, Leiden University, P.O. Box 9502, 2300 RA Leiden, The Netherlands*

‡*PLASMANT, Department of Chemistry, University of Antwerp, BE-2610 Antwerp, Belgium*

¶*ALTEN Nederland, Technology, Fascinatio Boulevard 582, 2909 VA Capelle a/d IJssel, The Netherlands*

E-mail: g.j.kroes@chem.leidenuniv.nl

Phone: +31 71 527 4396

Table S1 – Test of the effect of the pseudo-potential used on the barrier height for $N_2$ on Ru(0001) as obtained with the RPBE DF. Eb1: hard core pseudo-potential used only for $\boldsymbol{Ru}_{pv}$. Eb2: hard core pseudo-potential used for $\boldsymbol{Ru}_{pv}$ and $\boldsymbol{N}_h$.

| Ecut(eV) | Eb1(eV) | Eb2(eV) |
|---|---|---|
| 450 | 1.8385 | - |
| 500 | 1.8497 | 1.9233 |
| 550 | 1.8541 | 1.9603 |
| 600 | 1.8551 | 1.9734 |
| 650 | 1.8543 | 1.9755 |
| 700 | 1.8529 | 1.9776 |
| 750 | 1.8519 | 1.9805 |
| 800 | 1.8513 | 1.9834 |
| 850 | 1.8511 | 1.9852 |

Table S2 – The $r_{CH}$* value obtained through geometry optimization of $CH_4$(g) with each DF, which maybe compared with the RPBE value used throughout this work (1.097Å). E* is the energy obtained from $CH_4$ using the optimized $r_{CH}$* values and E is the value obtained using the RPBE value of $r_{CH}$.

| Functional | $r_{CH}$*(Å) | E*(eV) | E(eV) | dE (E-E*) |
|---|---|---|---|---|
| PBE | 1.096 | -24.000648 | -24.000562 | 0.000086 |
| RPBE | 1.097 | -24.006808 | -24.006808 | 0.000000 |
| SRP50 | 1.097 | -23.999024 | -23.999024 | 0.000000 |
| VdW-DF1 | 1.094 | -22.548263 | -22.547634 | 0.000629 |
| VdW-DF2 | 1.090 | -22.909629 | -22.906468 | 0.003161 |
| PBEvdW2 | 1.093 | -22.810216 | -22.809052 | 0.001164 |
| PBE$\alpha$57-vdW-DF2 | 1.092 | -22.808433 | -22.806933 | 0.001500 |
| SRP32-vdW-DF1 | 1.093 | -22.521826 | -22.520880 | 0.000946 |
| BEEF-vdW-DF2 | 1.089 | -23.239031 | -23.234939 | 0.004092 |
| revTPSS | 1.091 | -25.677106 | -25.675045 | 0.002061 |
| SCAN | 1.088 | -25.096148 | -25.091181 | 0.004967 |
| MS-B86bl | 1.097 | -25.457381 | -25.457381 | 0.000000 |
| MS2 | 1.094 | -26.620795 | -26.620189 | 0.000606 |

Table S3 – Parameters used in this work, in the earlier work on the SBH10 database,[1] and in calculations providing the reference energy from theory (here labelled "SRP14") are compared.

| basis | Layer | $N_x$ | $N_y$ | cut off(eV) | k-points | potcar | smear | vacuum(in Å) |
|---|---|---|---|---|---|---|---|---|
| | | | | $H_2$ + Cu(111) | | | | |
| SBH10 | 6 | 2 | 2 | 350 | 4 | paw | gaussian | 21 |
| SRP14[2] | 4 | 2 | 2 | 350 | 8 | uspp | fermi | 13 |
| this work | 5 | 3 | 3 | 450 | 11 | paw | methfessel-paxton | 13 |
| | | | | $H_2$ + Cu(100) | | | | |
| SBH10 | 6 | 2 | 2 | 350 | 4 | paw | gaussian | 21 |
| SRP14[3] | 4 | 2 | 2 | 350 | 8 | uspp | fermi | 13 |
| this work | 5 | 3 | 3 | 450 | 11 | paw | methfessel-paxton | 13 |
| | | | | $H_2$ +Cu(110) | | | | |
| SBH10 | | | | | | | | |
| SRP14[4] | 5 | 2 | 2 | 400 | 9 | paw | fermi | 15 |
| this work | 5 | 3 | 2 | 450 | 9×7×1 | paw | methfessel-paxton | 13 |
| | | | | $H_2$ + Pt(111) | | | | |
| SBH10 | 6 | 2 | 2 | 350 | 4 | paw | gaussian | 15 |
| SRP14[5] | 5 | 3 | 3 | 400 | 9 | paw | fermi | 13 |
| this work | 5 | 3 | 3 | 400 | 9 | paw | fermi | 13 |
| | | | | $H_2$ +Pt(211) | | | | |
| SBH10 | | | | | | | | |
| SRP14[6] | 4 | 1 | 2 | 450 | 7 | paw | fermi | 18 |
| this work | 5 | 1 | 3 | 450 | 8 | paw | fermi | 18 |
| | | | | $H_2$ +Ni(111) | | | | |
| SBH10 | | | | | | | | |
| SRP14[7] | 4 | 3 | 3 | 400 | 5 | paw | methfessel-paxton | 18 |
| this work | 5 | 3 | 3 | 450 | 8 | paw | methfessel-paxton | 18 |
| | | | | $H_2$ +Ag(111) | | | | |
| SBH10 | | | | | | | | |
| SRP14[8] | 5 | 3 | 3 | 450 | 11 | paw | methfessel-paxton | 16 |
| this work | 5 | 3 | 3 | 450 | 11 | paw | methfessel-paxton | 16 |
| | | | | $H_2$ +Ru(0001) | | | | |
| SBH10 | 6 | 2 | 2 | 350 | 4 | paw | gaussian | 15 |
| SRP14[9] | 5 | 2 | 2 | 350 | 8 | paw | fermi | 13 |
| this work | 5 | 3 | 3 | 450 | 8 | paw | fermi | 13 |

Table S3 Continued.

| basis | Layer | $N_x$ | $N_y$ | cut off(eV) | k-points | potcar | smear | vacuum(in Å) |
|---|---|---|---|---|---|---|---|---|
| | | | | $N_2$ +Ru(0001) | | | | |
| SBH10 | 6 | 2 | 2 | 350 | 4 | paw | gaussian | 15 |
| SRP14[10] | 7 | 3 | 3 | 550 | 7 | paw | methfessel-paxton | 13 |
| this work | 7 | 3 | 3 | 550 | 8 | paw | methfessel-paxton | 13 |
| | | | | $N_2$ +Ru(10$\bar{1}$0) | | | | |
| SBH10 | 6 | 2 | 3 | 350 | 4 | paw | gaussian | 15 |
| SRP14 | | | | | | | | |
| this work | 6 | 2 | 4 | 550 | 8 | paw | methfessel-paxton | 16 |
| | | | | $CH_4$ + Ni(111) | | | | |
| SBH10 | 4 | 3 | 3 | 350 | 4 | paw | gaussian | 15 |
| SRP14[11] | 4 | 3 | 3 | 350 | 4 | paw | fermi | 13 |
| this work | 4 | 3 | 3 | 350 | 4 | paw | fermi | 13 |
| | | | | $CH_4$ +Ni(100) | | | | |
| SBH10 | 6 | 2 | 2 | 350 | 4 | paw | gaussian | 16 |
| SRP14 | | | | | | | | |
| this work | 4 | 3 | 3 | 450 | 8 | paw | fermi | 18 |
| | | | | $CH_4$ +Ni(211) | | | | |
| SBH10 | 4 | 1 | 3 | 350 | 4 | paw | gaussian | 16 |
| SRP14[12] | 4 | 1 | 3 | | 8 | paw | | 16.1 |
| this work | 4 | 1 | 3 | 450 | 8 | paw | fermi | 18 |
| | | | | $CH_4$ + Pt(111) | | | | |
| SBH10 | | | | | | | | |
| SRP14[13] | 5 | 3 | 3 | 350 | 4 | paw | fermi | 13 |
| this work | 5 | 3 | 3 | 350 | 4 | paw | fermi | 13 |
| | | | | $CH_4$ + Pt(211) | | | | |
| SBH10 | | | | | 4 | | | |
| SRP14[13] | 4 | 1 | 3 | 350 | 4 | paw | fermi | 13 |
| this work | 4 | 1 | 3 | 400 | 8 | paw | fermi | 13 |
| | | | | $CH_4$ +Ru(0001) | | | | |
| SBH10 | 6 | 2 | 2 | 350 | 4 | paw | gaussian | 16 |
| SRP14 | | | | | | | | |
| this work | 5 | 3 | 3 | 450 | 8 | paw | methfessel-paxton | 16 |
| | | | | $CH_4$ +Ir(111) | | | | |
| SBH10 | | | | | | | | |
| SRP14[14] | 5 | 3 | 3 | 450 | 5 | paw | | 13.6 |
| this work | 5 | 3 | 3 | 450 | 5 | paw | methfessel-paxton | 27 |

Table S4 – $H_2$+Cu(100) barrier heights (in eV) for all the DFs and algorithms tested. We also provide values from the literature when available. The acronym "CRP" means that the barrier height was obtained from a fit to the DFT data.

| Functional | High Algo | Light Algo | Medium Algo | Literature values |
|---|---|---|---|---|
| | | GGA | | |
| PBE | 0.577 | 0.553 | 0.578 | 0.57(CRP)[15] |
| RPBE | 0.890 | 0.896 | 0.900 | 0.927(CRP)[15] |
| SRP50 | 0.738 | 0.725 | 0.738 | |
| | | GGA+vdW | | |
| vdW-DF1 | 1.172 | 1.188 | 1.168 | 1.144(CRP)[15] |
| vdW-DF2 | 1.322 | 1.348 | 1.312 | |
| PBE-vdW-DF2 | 1.027 | 1.035 | 1.024 | 0.996(CRP)[15] |
| SRP32-vdW-DF1 | 0.998 | 1.007 | 0.995 | |
| PBEa57-vdW-DF2 | 0.861 | 0.859 | 0.859 | |
| BEEF-vdW-DF2 | 1.061 | 1.069 | 1.070 | |
| optPBE-vdW-DF1 | - | - | 0.85 | |
| | | meta-GGA | | |
| revTPSS | 0.728 | 0.699 | 0.746 | 0.665(CRP)[15] |
| SCAN | 0.486 | 0.409 | 0.500 | |
| MS-B86bl | 0.756 | 0.717 | 0.771 | |
| MS2 | 0.499 | 0.435 | 0.503 | |

Table S5 – $H_2$+Cu(110) barrier heights (in eV) for all the DFs and algorithms tested. We also provide values from the literature when available. The acronym "CRP" means that the barrier height was obtained from a fit to the DFT data.

| Functional | High Algo | Light Algo | Medium Algo | Literature values |
|---|---|---|---|---|
| | | GGA | | |
| PBE | 0.619 | 0.644 | 0.661 | 0.592(PW91-CRP)[16] |
| RPBE | 0.924 | 1.026 | 0.888 | |
| SRP50 | 0.773 | 0.835 | 0.777 | |
| | | GGA+vdW | | |
| vdW-DF1 | 1.181 | 1.351 | 1.097 | |
| vdW-DF2 | 1.288 | 1.510 | 1.072 | |
| PBE-vdW-DF2 | 1.044 | 1.173 | 0.995 | |
| SRP32-vdW-DF1 | 1.016 | 1.153 | 0.972 | |
| PBEa57-vdW-DF2 | 0.889 | 0.974 | 0.886 | |
| BEEF-vdW-DF2 | 1.096 | 1.214 | 1.088 | |
| optPBE-vdW-DF1 | - | - | 0.912 | |
| | | meta-GGA | | |
| revTPSS | 0.821 | 0.813 | 0.976 | |
| SCAN | 0.549 | 0.447 | 0.624 | |
| MS-B86bl | 0.852 | 0.813 | 0.976 | |
| MS2 | 0.56 | 0.524 | 0.782 | |

Table S6 – $H_2$+Pt(111) barrier heights (in eV) for all the DFs and algorithms tested. We also provide values from the literature when available. The acronym "CRP" means that the barrier height was obtained from a fit to the DFT data.

| Functional | High Algo | Light Algo | Medium Algo | Literature values |
|---|---|---|---|---|
| | | GGA | | |
| PBE | 0.0383 | 0.070 | 0.0299 | 0.039(CRP) [15] |
| RPBE | 0.181 | 0.236 | 0.181 | 0.177(CRP) [15] |
| SRP50 | 0.106 | 0.153 | 0.105 | 0.102(SRP48) [17] |
| | | GGA+vdW | | |
| vdW-DF1 | 0.176 | 0.216 | 0.139 | 0.172(CRP) [15] |
| vdW-DF2 | 0.346(late barr) | 0.217 | 0.099 | |
| PBE-vdW-DF2 | 0.074 | 0.133 | 0.063 | 0.071(CRP) [15] |
| SRP32-vdW-DF1 | 0.126(late barr) | 0.114 | 0.043 | |
| PBE$\alpha$57-vdW-DF2 | -0.011 | 0.050 | -0.011 | -0.008(CRP) [5] |
| BEEF-vdW-DF2 | 0.143 | 0.171 | 0.118 | |
| optPBE-vdW-DF1 | - | - | 0.018 | |
| | | meta-GGA | | |
| revTPSS | 0.154 | 0.096 | 0.088 | 0.302(CRP) [15] |
| SCAN | -0.003 | -0.012 | -0.006 | |
| MS-B86bl | 0.191 | 0.189 | 0.192 | 0.194(CRP) [18] |
| MS2 | -0.010 | -0.024 | -0.022 | |

Table S7 – $H_2$+Pt(211) barrier heights (in eV) for all the DFs and algorithms tested. We also provide values from the literature when available. The acronym "CRP" means that the barrier height was obtained from a fit to the DFT data.

| Functional | High Algo | Light Algo | Medium Algo | Literature values |
|---|---|---|---|---|
| | | GGA | | |
| PBE | - | -0.0285 | -0.0253 | |
| RPBE | 0.053 | 0.0407 | 0.0398 | |
| SRP50 | - | 0.006 | 0.007 | |
| | | GGA+vdW | | |
| vdW-DF1 | -0.008 | -0.018 | -0.027 | |
| vdW-DF2 | 0.084 | -0.035 | -0.047 | |
| PBE-vdW-DF2 | -0.140 | -0.049 | -0.056 | |
| SRP32-vdW-DF1 | -0.155 | -0.072 | -0.079 | |
| PBE$\alpha$57-vdW-DF2 | -0.277 | -0.083 | -0.088 | -0.083(CRP)[6] |
| BEEF-vdW-DF2 | -0.074 | -0.022 | -0.026 | |
| optPBE-vdW-DF1 | - | - | -0.070 | |
| | | meta-GGA | | |
| revTPSS | -0.185 | -0.013 | -0.005 | |
| SCAN | - | -0.062 | -0.045 | |
| MS-B86bl | - | 0.047 | 0.058 | |
| MS2 | -0.53 | -0.064 | -0.123 | |

Table S8 – $H_2$+Ni(111) barrier heights (in eV) for all the DFs and algorithms tested. We also provide values from the literature when available. The acronym "CRP" means that the barrier height was obtained from a fit to the DFT data.

| Functional | High Algo | Light Algo | Medium Algo | Literature values |
|---|---|---|---|---|
| | | GGA | | |
| PBE | 0.0484 | 0.032 | 0.025 | 0.011(CRP)[7] |
| RPBE | 0.170 | 0.192 | 0.169 | |
| SRP50 | 0.107 | 0.112 | 0.098 | |
| | | GGA+vdW | | |
| vdW-DF1 | 0.132 | 0.161 | 0.129 | |
| vdW-DF2 | 0.105 | 0.153 | 0.099 | |
| PBE-vdW-DF2 | 0.0853 | 0.093 | 0.0853 | 0.024(CRP)[7] |
| SRP32-vdW-DF1 | 0.0362 | 0.062 | 0.0366 | |
| PBE$\alpha$57-vdW-DF2 | 0.001 | 0.016 | 0.0017 | -0.05(CRP)[7] |
| BEEF-vdW-DF2 | 0.123 | 0.138 | 0.122 | |
| optPBE-vdW-DF1 | - | - | 0.0614 | |
| | | meta-GGA | | |
| revTPSS | 0.066 | 0.034 | 0.053 | |
| SCAN | 0.0163 | -0.011 | 0.006 | |
| MS-B86bl | 0.054 | 0.159 | 0.172 | |
| MS2 | -0.258 | -0.056 | -0.035 | |

Table S9 – $H_2$+Ag(111) barrier heights (in eV) for all the DFs and algorithms tested. We also provide values from the literature when available. The acronym "CRP" means that the barrier height was obtained from a fit to the DFT data.

| Functional | High Algo | Light Algo | Medium Algo | Literature values |
|---|---|---|---|---|
| | | GGA | | |
| PBE | 1.135 | 1.181 | 1.138 | |
| RPBE | 1.467 | 1.539 | 1.466 | |
| SRP50 | 1.301 | 1.361 | 1.302 | |
| | | GGA+vdW | | |
| vdW-DF1 | 1.725 | 1.821 | 1.714 | |
| vdW-DF2 | 1.826 | 1.992 | 1.811 | |
| PBE-vdW-DF2 | 1.578 | 1.662 | 1.569 | |
| SRP32-vdW-DF1 | 1.551 | 1.634 | 1.542 | |
| PBEa57-vdW-DF2 | 1.409 | 1.476 | 1.403 | 1.409(CRP) [19] |
| BEEF-vdW-DF2 | 1.672 | 1.731 | 1.660 | |
| optPBE-vdW-DF1 | - | - | 1.414 | |
| | | meta-GGA | | |
| revTPSS | 1.311 | | 1.313 | |
| SCAN | 1.085 | 1.103 | 1.091 | |
| MS-B86bl | 1.299 | 1.309 | 1.296 | 1.342(CRP) [18] |
| MS2 | 1.046 | 1.048 | 1.048 | |

Table S10 – $H_2$+Ru(0001) barrier heights (in eV) for all the DFs and algorithms tested. We also provide values from the literature when available. The acronym "CRP" means that the barrier height was obtained from a fit to the DFT data.

| Functional | High Algo | Light Algo | Medium Algo | Literature values |
|---|---|---|---|---|
| | | GGA | | |
| PBE | 0.0181 | 0.0188 | 0.0171 | 0.022[9] |
| RPBE | 0.111 | 0.121 | 0.110 | 0.116(CRP)[15] |
| SRP50 | 0.0617 | 0.07 | 0.0622 | 0.066(SRP48)[17] |
| | | GGA+vdW | | |
| vdW-DF1 | 0.057 | 0.068 | 0.0479 | 0.057(CRP)[15] |
| vdW-DF2 | 0.0159 | 0.047 | 0.011 | |
| PBE-vdW-DF2 | 0.003 | 0.0193 | 0.002 | 0.004[9] |
| SRP32-vdW-DF1 | -0.018 | -0.0017 | -0.019 | |
| PBE$\alpha$57-vdW-DF2 | -0.043 | -0.030 | -0.0439 | |
| BEEF-vdW-DF2 | 0.041 | 0.046 | 0.0363 | |
| optPBE-vdW-DF1 | - | - | -0.0127 | |
| | | meta-GGA | | |
| revTPSS | 0.034 | 0.0166 | 0.023 | 0.041(CRP)[15] |
| SCAN | -0.0196 | -0.034 | -0.03 | |
| MS-B86bl | 0.116 | 0.114 | 0.118 | |
| MS2 | -0.440 | -0.0389 | -0.0348 | |

Table S11 – $N_2$+Ru(0001) barrier heights (in eV) for all the DFs and algorithms tested. We also provide values from the literature when available.

| Functional | High Algo | Light Algo | Medium Algo | Literature values |
|---|---|---|---|---|
| | | GGA | | |
| PBE | 1.493 | 1.401 | 1.524 | |
| RPBE | 1.953 | 1.893 | 1.955 | 1.84[10] |
| SRP50 | 1.726 | 1.646 | 1.741 | |
| | | GGA+vdW | | |
| vdW-DF1 | 1.898 | 1.945 | 1.895 | |
| vdW-DF2 | 2.156 | 2.443 | 2.184 | |
| PBE-vdW-DF2 | 1.752 | 1.772 | 1.746 | |
| SRP32-vdW-DF1 | 1.658 | 1.682 | 1.659 | |
| PBE$\alpha$57-vdW-DF2 | 1.520 | 1.510 | 1.528 | |
| BEEF-vdW-DF2 | 1.890 | 1.812 | 1.885 | |
| optPBE-vdW-DF1 | - | - | 1.482 | |
| | | meta-GGA | | |
| revTPSS | 1.250 | 1.027 | 1.250 | |
| SCAN | 1.471 | 1.241 | 1.526 | |
| MS-B86bl | 1.900 | 1.673 | 1.918 | |
| MS2 | 1.463 | 1.263 | 1.510 | |

Table S12 – $N_2$+Ru(10$\bar{1}$0) barrier heights (in eV) for all the DFs and algorithms tested.

| Functional | High Algo | Light Algo | Medium Algo |
|---|---|---|---|
| | | GGA | |
| PBE | -0.104 | -0.160 | -0.098 |
| RPBE | 0.466 | 0.448 | 0.466 |
| SRP50 | 0.182 | 0.144 | 0.185 |
| | | GGA+vdW | |
| vdW-DF1 | 0.442 | 0.476 | 0.441 |
| vdW-DF2 | 0.801 | 0.972 | 0.804 |
| PBE-vdW-DF2 | 0.246 | 0.256 | 0.247 |
| SRP32-vdW-DF1 | 0.146 | 0.158 | 0.146 |
| PBE$\alpha$57-vdW-DF2 | -0.048 | -0.065 | -0.044 |
| BEEF-vdW-DF2 | 0.412 | 0.376 | 0.408 |
| optPBE-vdW-DF1 | - | - | -0.111 |
| | | meta-GGA | |
| revTPSS | -0.449 | -0.576 | -0.454 |
| SCAN | -0.345 | -0.480 | -0.335 |
| MS-B86bl | 0.275 | 0.162 | 0.274 |
| MS2 | -0.200 | -0.32 | -0.178 |

Table S13 – $CH_4$+Ni(111) barrier heights (in eV) for all the DFs and algorithms tested. We also provide values from the literature when available.

| Functional | High Algo | Light Algo | Medium Algo | Literature values |
|---|---|---|---|---|
| | | GGA | | |
| PBE | 0.996 | 1.022 | 1.006 | 1.065[20]<br>1.08[21] |
| RPBE | 1.351 | 1.404 | 1.349 | |
| SRP50 | 1.176 | 1.213 | 1.178 | |
| | | GGA+vdW | | |
| vdW-DF1 | 1.255 | 1.337 | 1.262 | |
| vdW-DF2 | 1.300 | 1.469 | 1.348 | |
| PBE-vdW-DF2 | 1.151 | 1.184 | 1.156 | |
| SRP32-vdW-DF1 | 1.066 | 1.128 | 1.068 | 1.015[11] |
| PBE$\alpha$57-vdW-DF2 | 0.948 | 0.986 | 0.948 | |
| BEEF-vdW-DF2 | 1.231 | 1.266 | 1.230 | |
| optPBE-vdW-DF1 | - | - | 1.001 | |
| | | meta-GGA | | |
| revTPSS | 0.94 | 0.939 | 1.005 | |
| SCAN | 0.936 | 0.900 | 0.971 | |
| MS-B86bl | 1.316 | 1.312 | 1.352 | |
| MS2 | 0.949 | 0.949 | 1.025 | |

Table S14 – $CH_4$+Ni(100) barrier heights (in eV) for all the DFs and algorithms tested. We also provide values from the literature when available.

| Functional | High Algo | Light Algo | Medium Algo | Literature values |
|---|---|---|---|---|
| | | GGA | | |
| PBE | 0.908 | 0.894 | 0.913 | 0.91 [20] |
| RPBE | 1.263 | 1.275 | 1.262 | |
| SRP50 | 1.089 | 1.085 | 1.088 | |
| | | GGA+vdW | | |
| vdW-DF1 | 1.136 | 1.182 | 1.147 | |
| vdW-DF2 | 1.134 | 1.264 | 1.187 | |
| PBE-vdW-DF2 | 1.02 | 1.027 | 1.02 | |
| SRP32-vdW-DF1 | 0.948 | 0.974 | 0.952 | |
| PBE$\alpha$57-vdW-DF2 | 0.828 | 0.831 | 0.829 | |
| BEEF-vdW-DF2 | 1.141 | 1.145 | 1.141 | |
| optPBE-vdW-DF1 | - | - | 0.849 | |
| | | meta-GGA | | |
| revTPSS | 0.871 | 0.847 | 0.907 | |
| SCAN | 0.855 | 0.789 | 0.874 | |
| MS-B86bl | 1.233 | 1.208 | 1.257 | |
| MS2 | 0.890 | 0.868 | 0.934 | |

Table S15 – $CH_4$+Ni(211) barrier heights (in eV) for all the DFs and algorithms tested. We also provide values from the literature when available.

| Functional | High Algo | Light Algo | Medium Algo | Literature values |
|---|---|---|---|---|
| | | GGA | | |
| PBE | 0.643 | 0.615 | 0.678 | |
| RPBE | 0.971 | 0.950 | 0.974 | |
| SRP50 | 0.809 | 0.782 | 0.826 | |
| | | GGA+vdW | | |
| vdW-DF1 | 0.871 | 0.870 | 0.875 | |
| vdW-DF2 | 0.911 | 0.952 | 0.951 | |
| PBE-vdW-DF2 | 0.761 | 0.752 | 0.778 | |
| SRP32-vdW-DF1 | 0.693 | 0.683 | 0.699 | 0.699 [12] |
| PBE$\alpha$57-vdW-DF2 | 0.599 | 0.578 | 0.613 | |
| BEEF-vdW-DF2 | 0.885 | 0.863 | 0.893 | |
| optPBE-vdW-DF1 | - | - | 0.610 | |
| | | meta-GGA | | |
| revTPSS | 0.614 | 0.602 | 0.723 | |
| SCAN | 0.57 | 0.532 | 0.686 | |
| MS-B86bl | 0.885 | 0.893 | 1.008 | |
| MS2 | 0.634 | 0.605 | 0.736 | |

Table S16 – $CH_4$+Pt(111) barrier heights (in eV) for all the DFs and algorithms tested. We also provide values from the literature when available.

| Functional | High Algo | Light Algo | Medium Algo | Literature values |
|---|---|---|---|---|
| | | GGA | | |
| PBE | 0.818 | 0.945 | 0.813 | 0.809 [22]<br>0.820 [23] |
| RPBE | 1.158 | 1.323 | 1.145 | 1.165 [22] |
| SRP50 | 0.990 | 1.134 | 0.979 | |
| | | GGA+vdW | | |
| vdW-DF1 | 1.089 | 1.293 | 1.041 | 1.061 [22] |
| vdW-DF2 | 1.168 | 1.525 | 1.138 | |
| PBE-vdW-DF2 | 0.94 | 1.128 | 0.900 | |
| SRP32-vdW-DF1 | 0.864 | 1.085 | 0.850 | 0.815 [13] |
| PBE$\alpha$57-vdW-DF2 | 0.738 | 0.929 | 0.726 | |
| BEEF-vdW-DF2 | 1.012 | 1.181 | 1.004 | |
| optPBE-vdW-DF1 | - | - | 0.773 | |
| | | meta-GGA | | |
| revTPSS | 0.856 | 0.896 | 0.878 | |
| SCAN | 0.566 | 0.594 | 0.606 | |
| MS-B86bl | 1.098 | 1.141 | 1.148 | |
| MS2 | 0.820 | 0.869 | 0.877 | |

Table S17 – $CH_4$+Pt(211) barrier heights (in eV) for all the DFs and algorithms tested. We also provide values from the literature when available.

| Functional | High Algo | Light Algo | Medium Algo | Literature values |
|---|---|---|---|---|
| | | GGA | | |
| PBE | 0.460 | 0.385 | 0.489 | 0.477[24] |
| RPBE | 0.774 | 0.722 | 0.789 | |
| SRP50 | 0.617 | 0.554 | 0.639 | |
| | | GGA+vdW | | |
| vdW-DF1 | 0.740 | 0.760 | 0.757 | |
| vdW-DF2 | 0.802 | 0.955 | 0.894 | |
| PBE-vdW-DF2 | 0.616 | 0.617 | 0.618 | |
| SRP32-vdW-DF1 | 0.567 | 0.574 | 0.584 | 0.559[24] |
| PBE$\alpha$57-vdW-DF2 | 0.458 | 0.439 | 0.473 | |
| BEEF-vdW-DF2 | 0.697 | 0.686 | 0.709 | |
| optPBE-vdW-DF1 | - | - | 0.466 | |
| | | meta-GGA | | |
| revTPSS | 0.514 | 0.3968 | 0.562 | |
| SCAN | 0.336 | 0.0543 | 0.688 | |
| MS-B86bl | 0.681 | 0.533 | 0.760 | |
| MS2 | 0.450 | 0.308 | 0.516 | |

Table S18 – $CH_4$+Ru(0001) barrier heights (in eV) for all the DFs and algorithms tested.

| Functional | High Algo | Light Algo | Medium Algo |
|---|---|---|---|
| | | GGA | |
| PBE | 0.844 | 0.859 | 0.853 |
| RPBE | 1.161 | 1.194 | 1.162 |
| SRP50 | 1.005 | 1.026 | 1.010 |
| | | GGA+vdW | |
| vdW-DF1 | 1.030 | 1.105 | 1.036 |
| vdW-DF2 | 1.102 | 1.301 | 1.155 |
| PBE-vdW-DF2 | 0.927 | 0.989 | 0.933 |
| SRP32-vdW-DF1 | 0.853 | 0.915 | 0.858 |
| PBE$\alpha$57-vdW-DF2 | 0.770 | 0.814 | 0.775 |
| BEEF-vdW-DF2 | 1.004 | 1.030 | 1.007 |
| optPBE-vdW-DF1 | - | - | 0.756 |
| | | meta-GGA | |
| revTPSS | 0.831 | 0.811 | 0.846 |
| SCAN | 0.763 | 0.73 | 0.779 |
| MS-B86bl | 1.128 | 1.104 | 1.142 |
| MS2 | 0.830 | 0.813 | 0.850 |

Table S19 – $CH_4$+Ir(111) barrier heights (in eV) for all the DFs and algorithms tested. We also provide values from the literature when available.

| Functional | High Algo | Light Algo | Medium Algo | Literature values |
|---|---|---|---|---|
| | | GGA | | |
| PBE | 0.817 | 0.953 | 0.846 | 0.83 [25] |
| RPBE | 1.153 | 1.305 | 1.162 | |
| SRP50 | 0.989 | 1.130 | 1.004 | |
| | | GGA+vdW | | |
| vdW-DF1 | 0.997 | 1.210 | 1.001 | |
| vdW-DF2 | 1.106 | 1.474 | 1.150 | |
| PBE-vdW-DF2 | 0.889 | 1.085 | 0.893 | |
| SRP32-vdW-DF1 | 0.813 | 1.011 | 0.816 | 0.836 [14] |
| PBE$\alpha$57-vdW-DF2 | 0.724 | 0.900 | 0.730 | |
| BEEF-vdW-DF2 | 0.975 | 1.105 | 0.981 | |
| optPBE-vdW-DF1 | - | - | 0.710 | |
| | | meta-GGA | | |
| revTPSS | 0.856 | 0.896 | 0.894 | |
| SCAN | 0.753 | 0.72 | 0.830 | |
| MS-B86bl | 1.095 | 1.134 | 1.151 | |
| MS2 | 0.820 | 0.851 | 0.875 | |

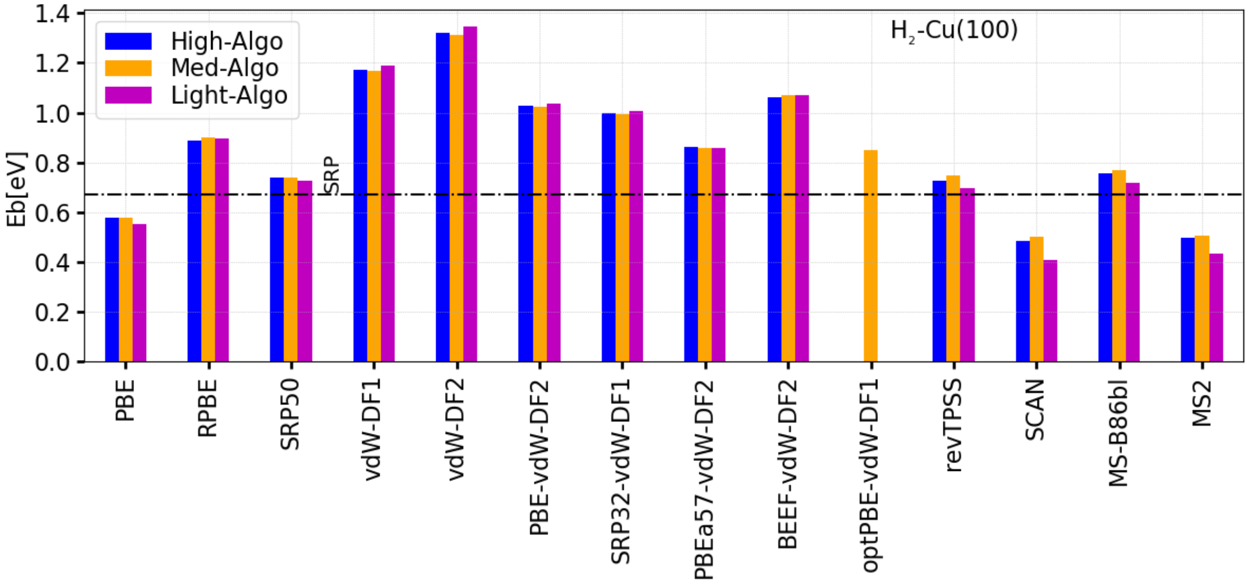


Fig. S1 – $H_2$+Cu(100) bar plot of barrier heights for all DFs and algorithms tested. The horizontal black dashed line is the SRP reference value.

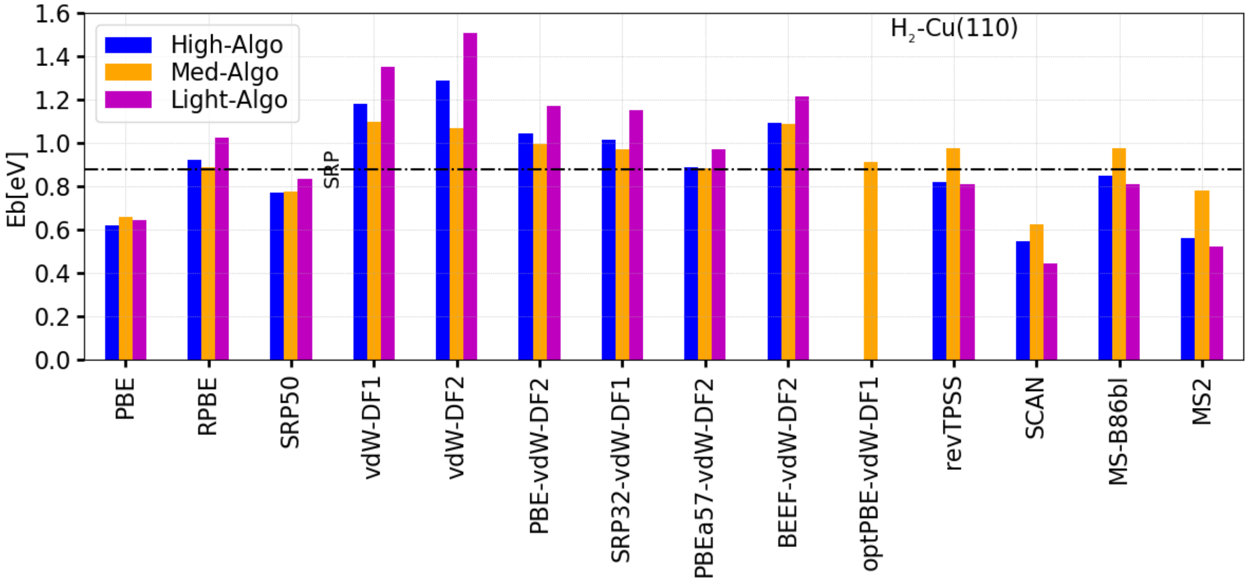


Fig. S2 – $H_2$+Cu(110) bar plot of barrier heights for all DFs and algorithms tested. The horizontal black dashed line is the SRP reference value.

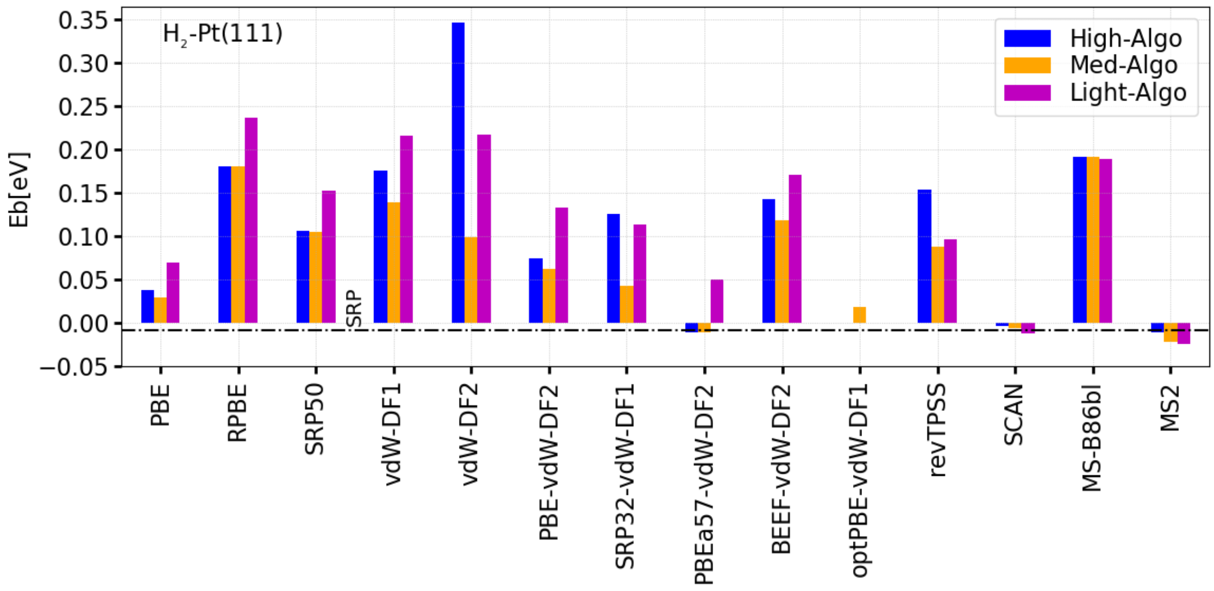


Fig. S3 – $H_2$+Pt(111) bar plot of barrier heights for all DFs and algorithms tested. The horizontal black dashed line is the SRP reference value.

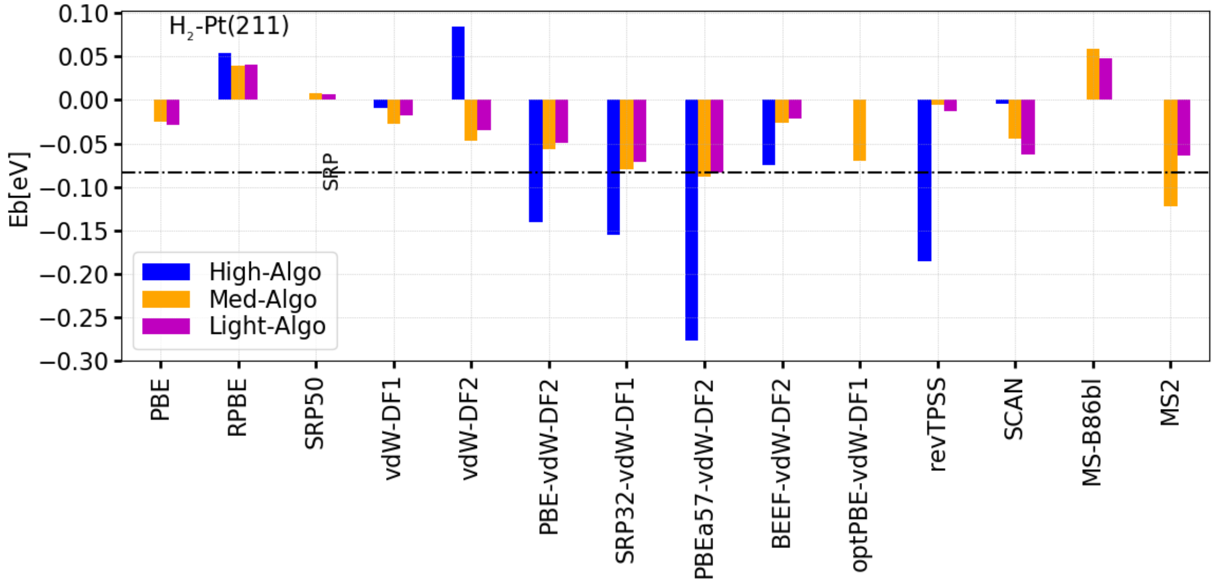


Fig. S4 – $H_2$+Pt(211) bar plot of barrier heights for all DFs and algorithms tested. The horizontal black dashed line is the SRP reference value.

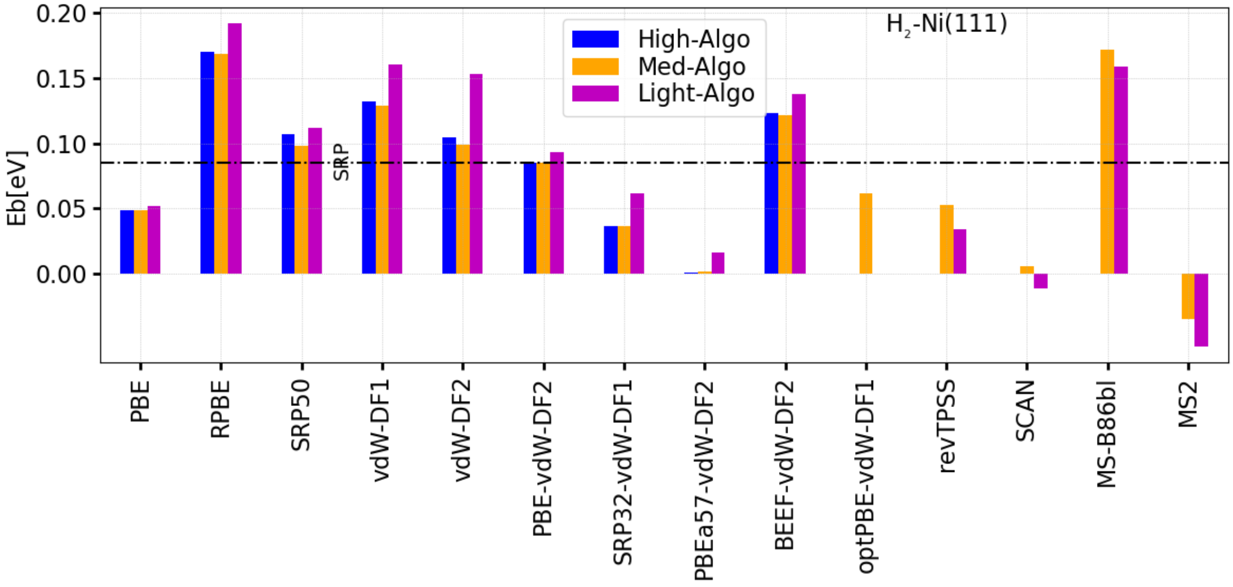


Fig. S5 – $H_2$+Ni(111) bar plot of barrier heights for all DFs and algorithms tested. The horizontal black dashed line is the SRP reference value.

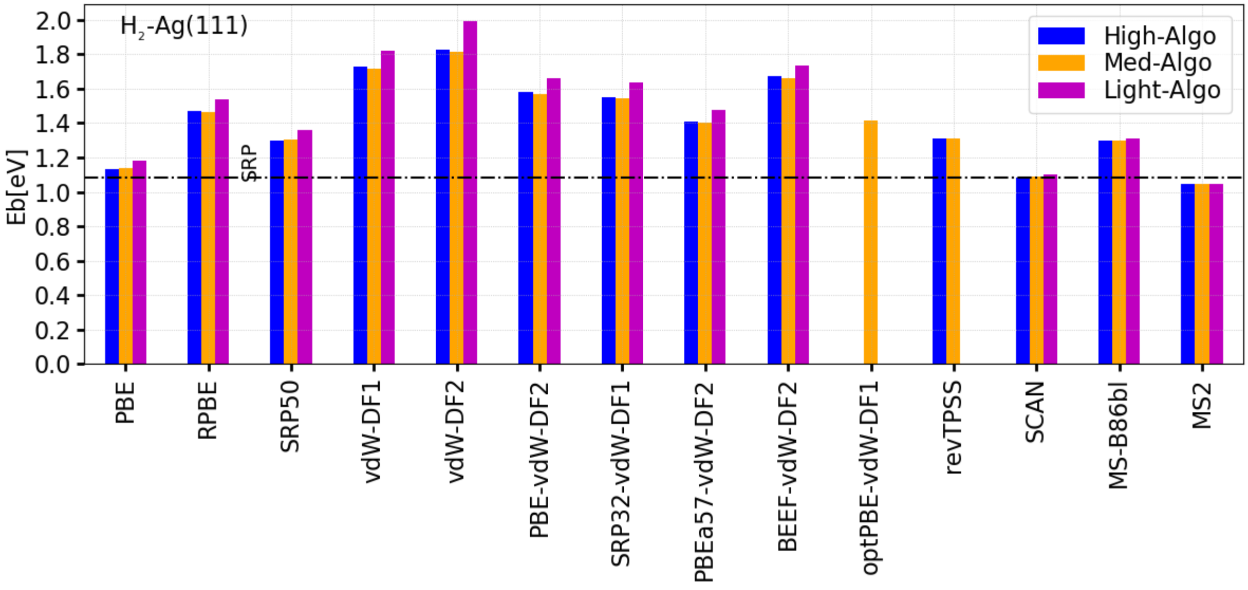


Fig. S6 – $H_2$+Ag(111) bar plot of barrier heights for all DFs and algorithms tested. The horizontal black dashed line is the SRP reference value.

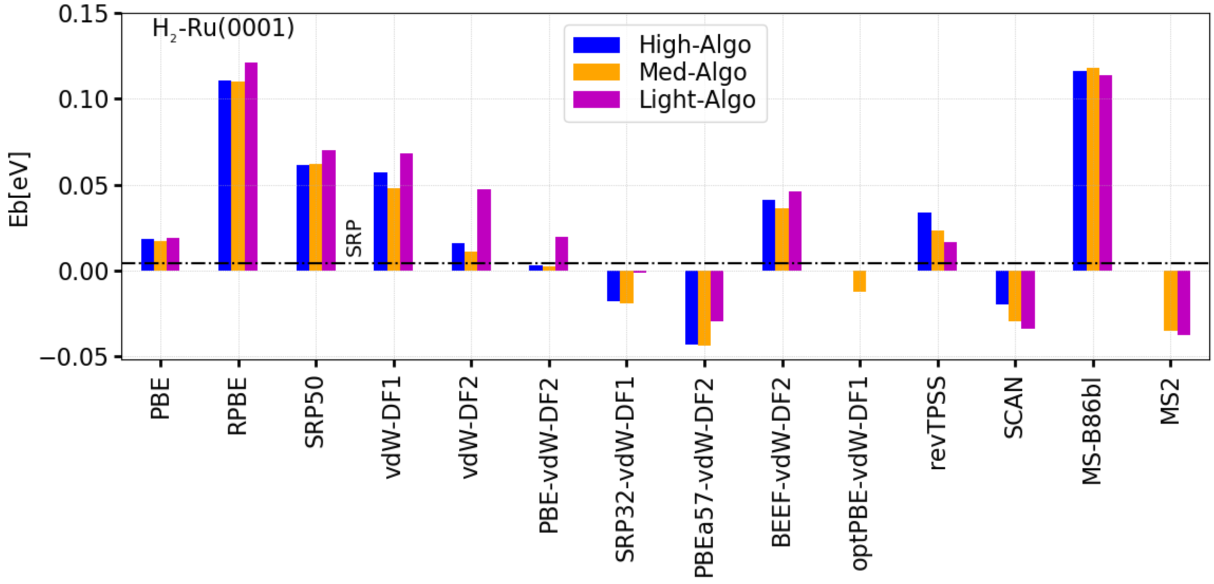


Fig. S7 – $H_2$+Ru(0001) bar plot of barrier heights for all DFs and algorithms tested. The horizontal black dashed line is the SRP reference value.

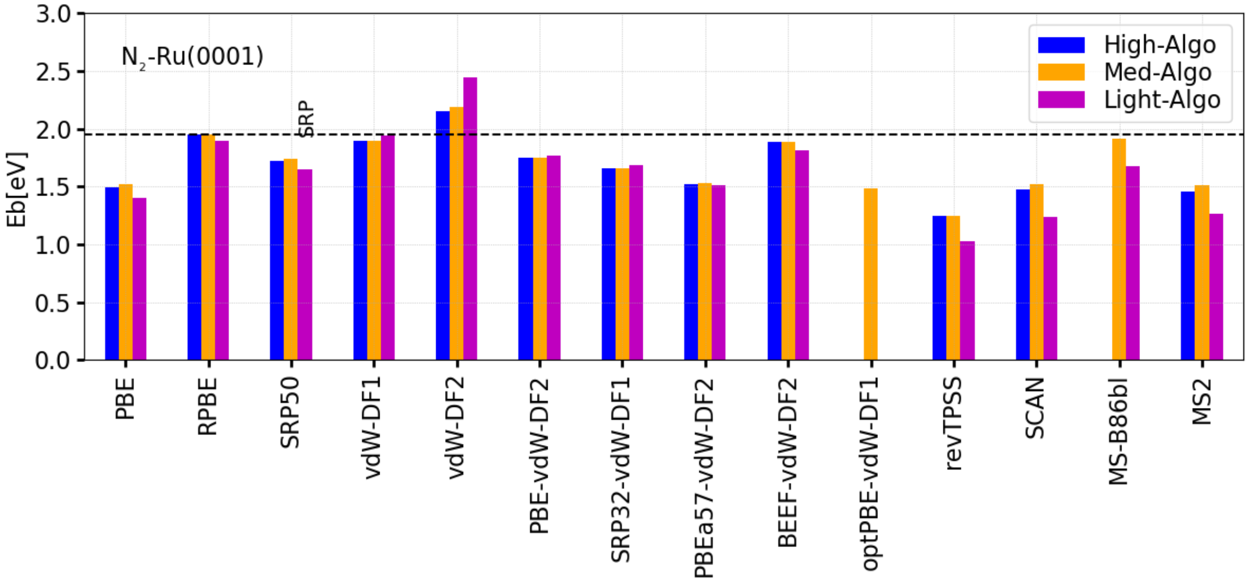


Fig. S8 – $N_2$+Ru(0001) bar plot of barrier heights for all DFs and algorithms tested. The horizontal black dashed line is the SRP reference value.

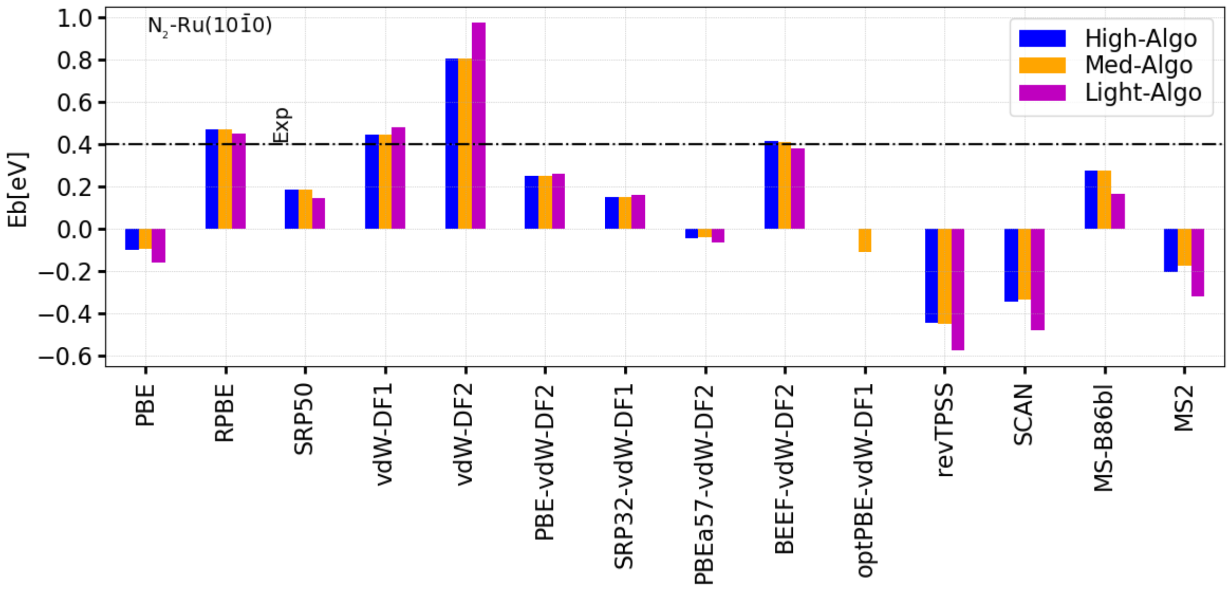


Fig. S9 – $N_2$+Ru(10$\bar{1}$0) bar plot of barrier heights for all DFs and algorithms tested. The horizontal black dashed line is the SRP reference value.

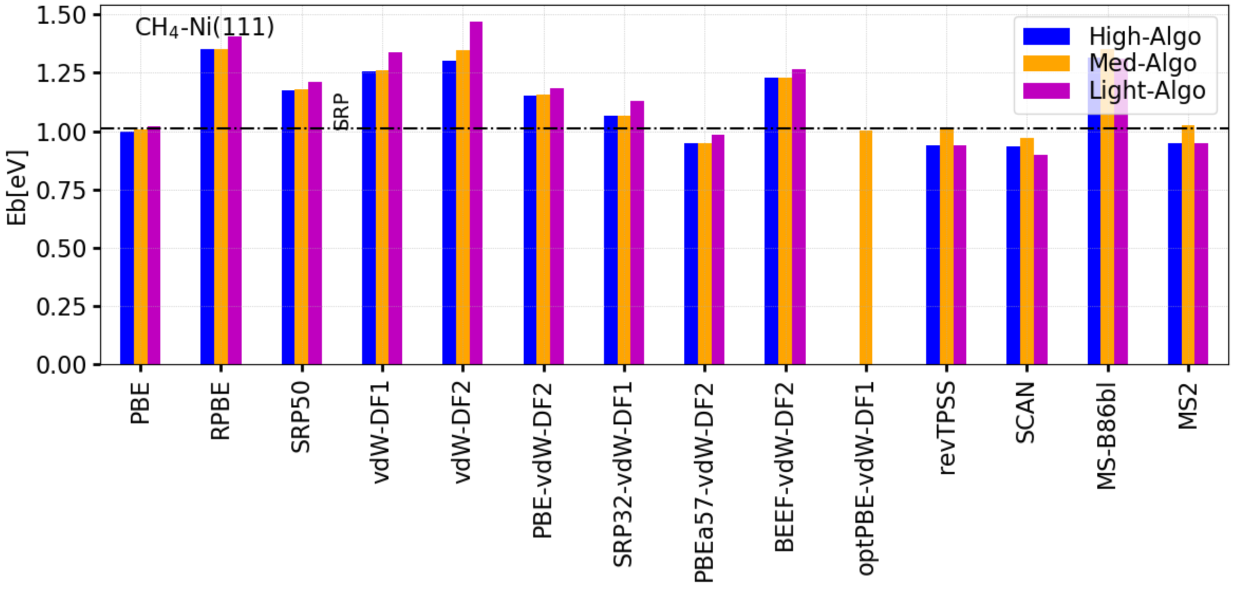


Fig. S10 – $CH_4$+Ni(111) bar plot of barrier heights for all DFs and algorithms tested. The horizontal black dashed line is the SRP reference value.

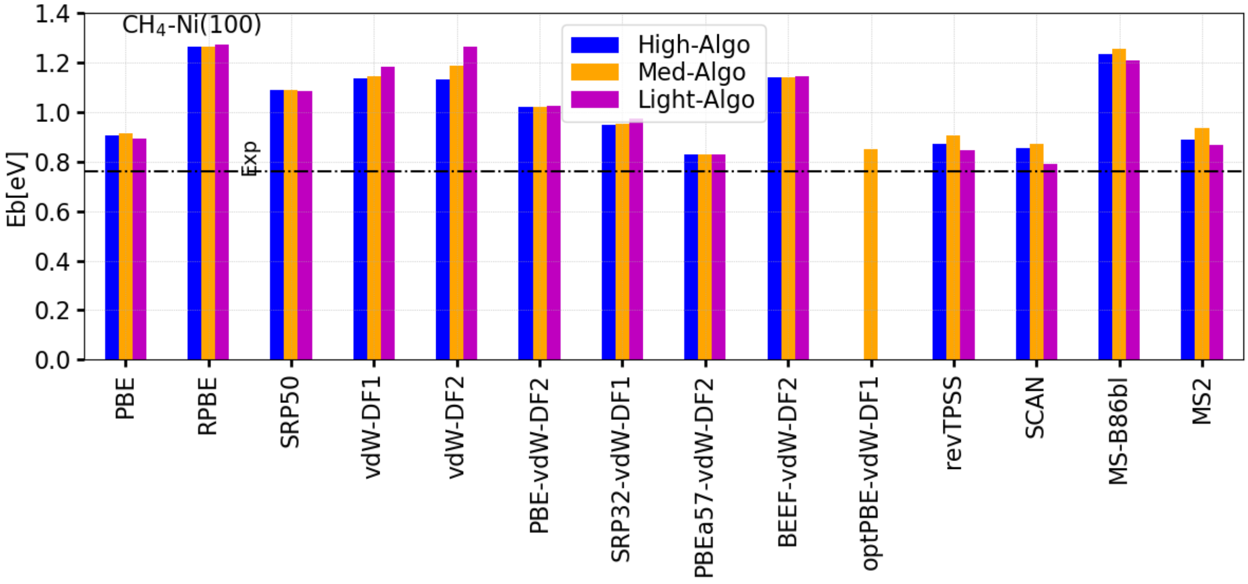


Fig. S11 – $CH_4$+Ni(100) bar plot of barrier heights for all DFs and algorithms tested. The horizontal black dashed line is the SRP reference value.

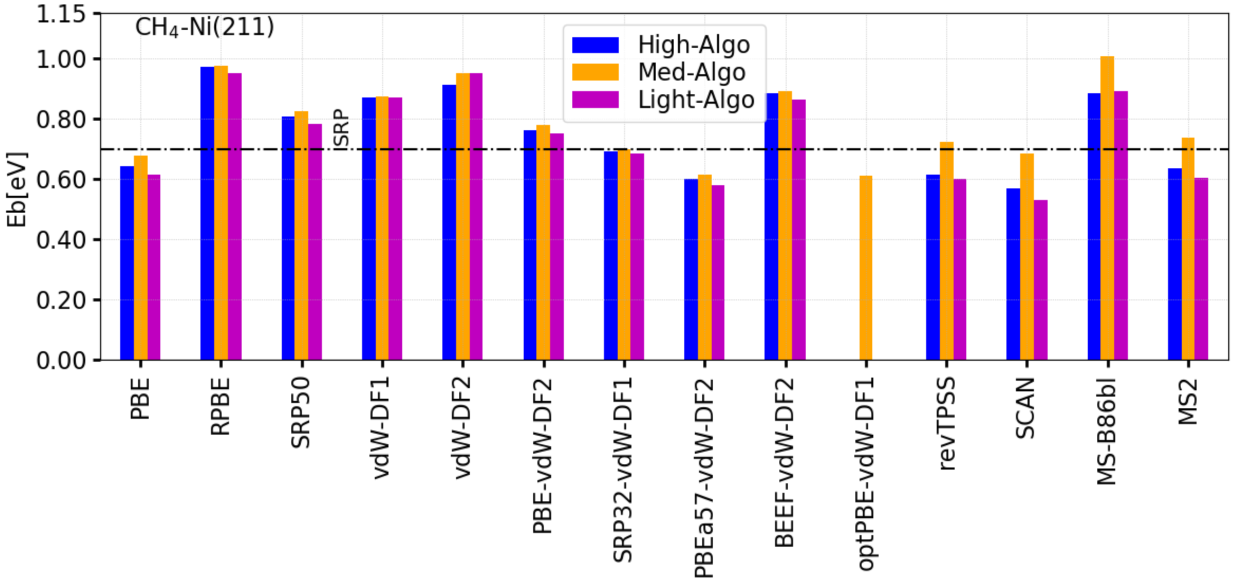


Fig. S12 – $CH_4$+Ni(211) bar plot of barrier heights for all DFs and algorithms tested. The horizontal black dashed line is the SRP reference value.

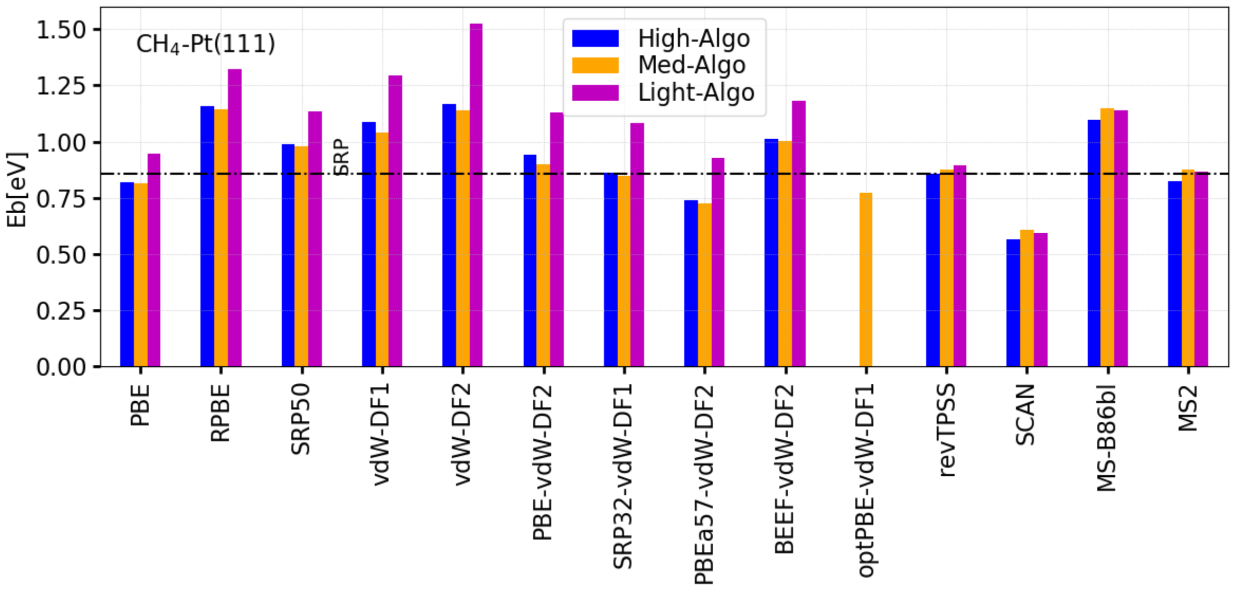


Fig. S13 – $CH_4$+Pt(111) bar plot of barrier heights for all DFs and algorithms tested. The horizontal black dashed line is the SRP reference value.

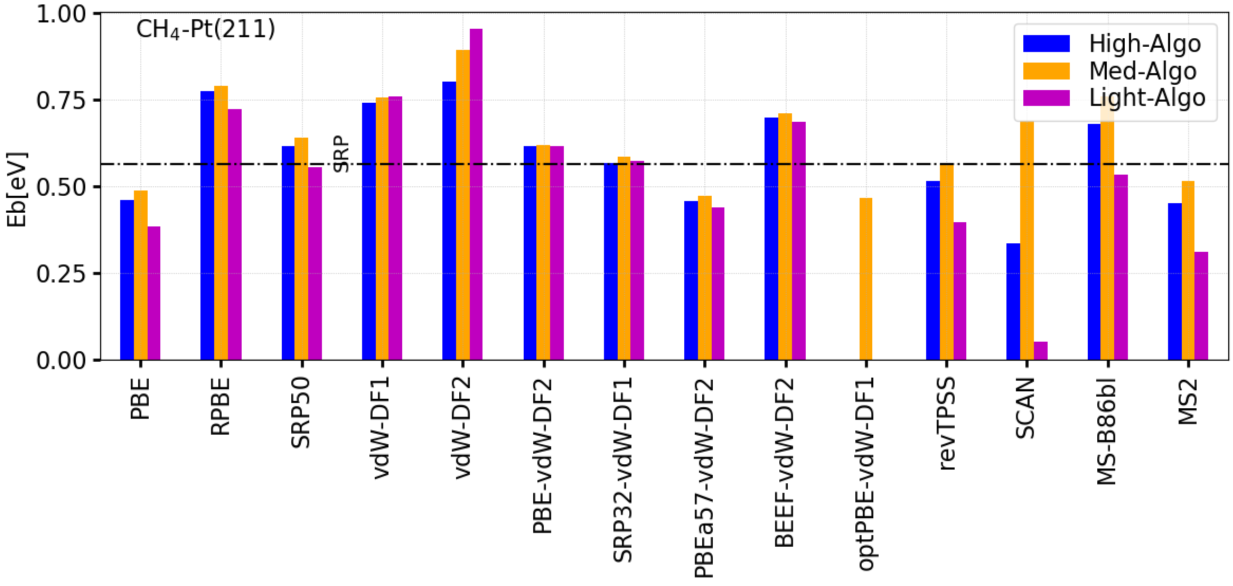


Fig. S14 – $CH_4$+Pt(211) bar plot of barrier heights for all DFs and algorithms tested. The horizontal black dashed line is the SRP reference value.

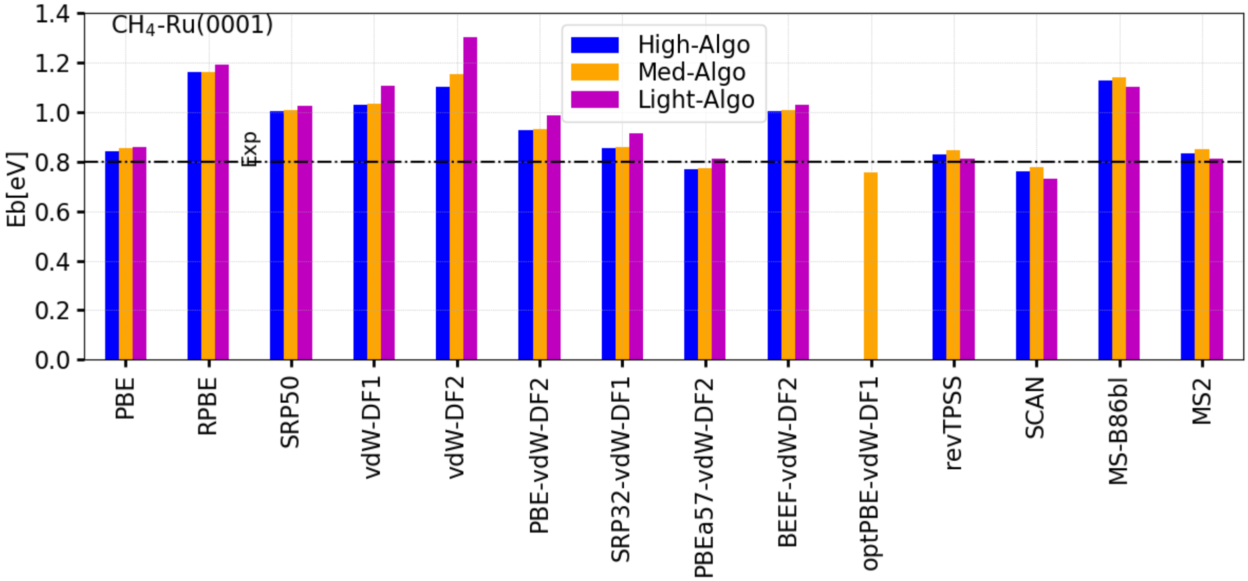


Fig. S15 – $CH_4$+Ru(0001) bar plot of barrier heights for all DFs and algorithms tested. The horizontal black dashed line is the SRP reference value.

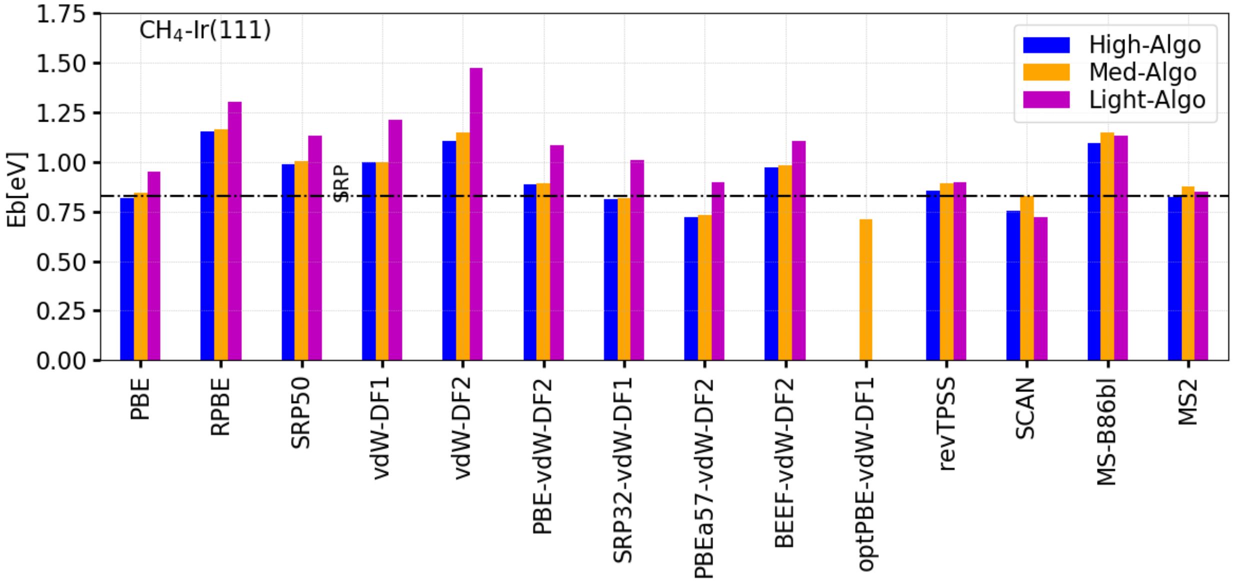


Fig. S16 – $CH_4$+Ir(111) bar plot of barrier heights for all DFs and algorithms tested. The horizontal black dashed line is the SRP reference value.

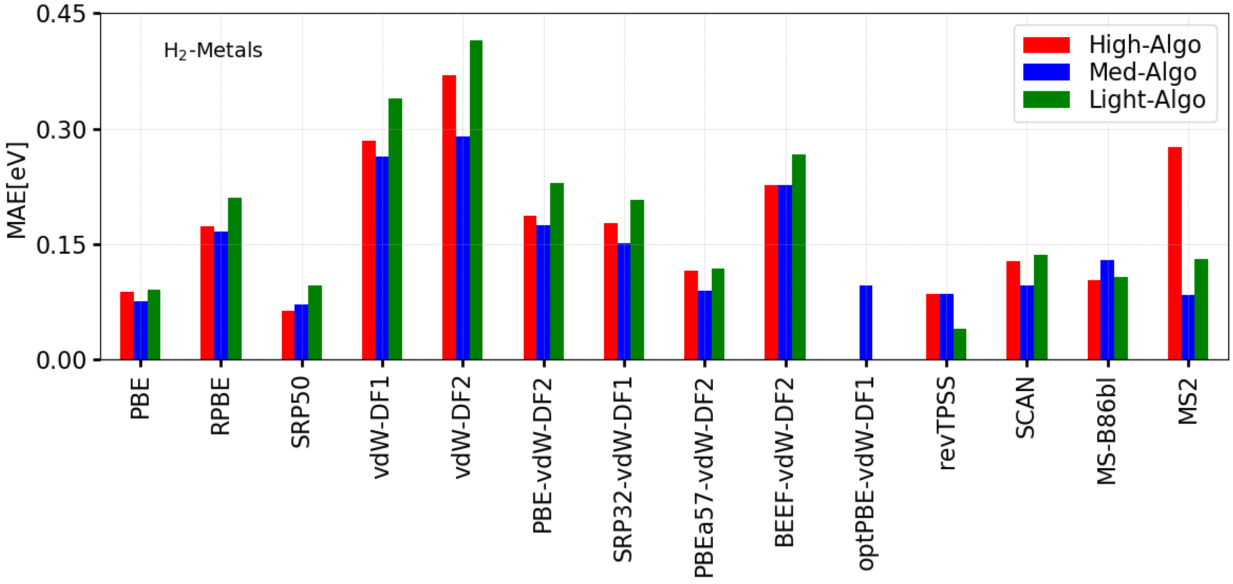


Fig. S17 – Bar plot of the mean absolute error obtained for $H_2$-metal surface systems for the three algorithms for all DFs tested.

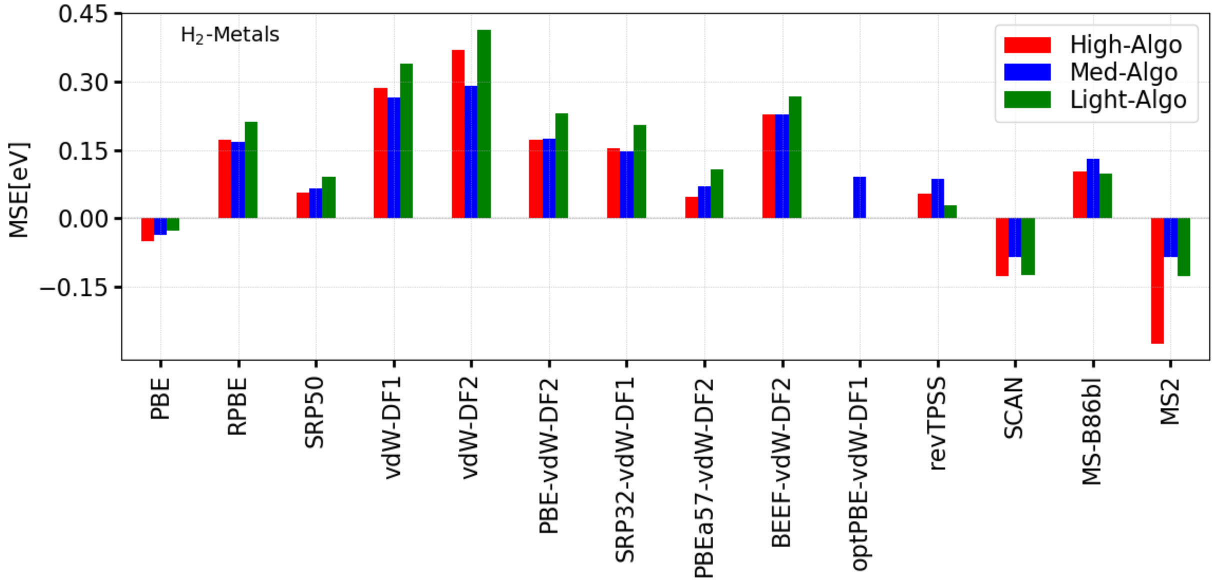


Fig. S18 – Bar plot of the mean signed error obtained for $H_2$-metal surface systems for the three algorithms for all DFs tested.

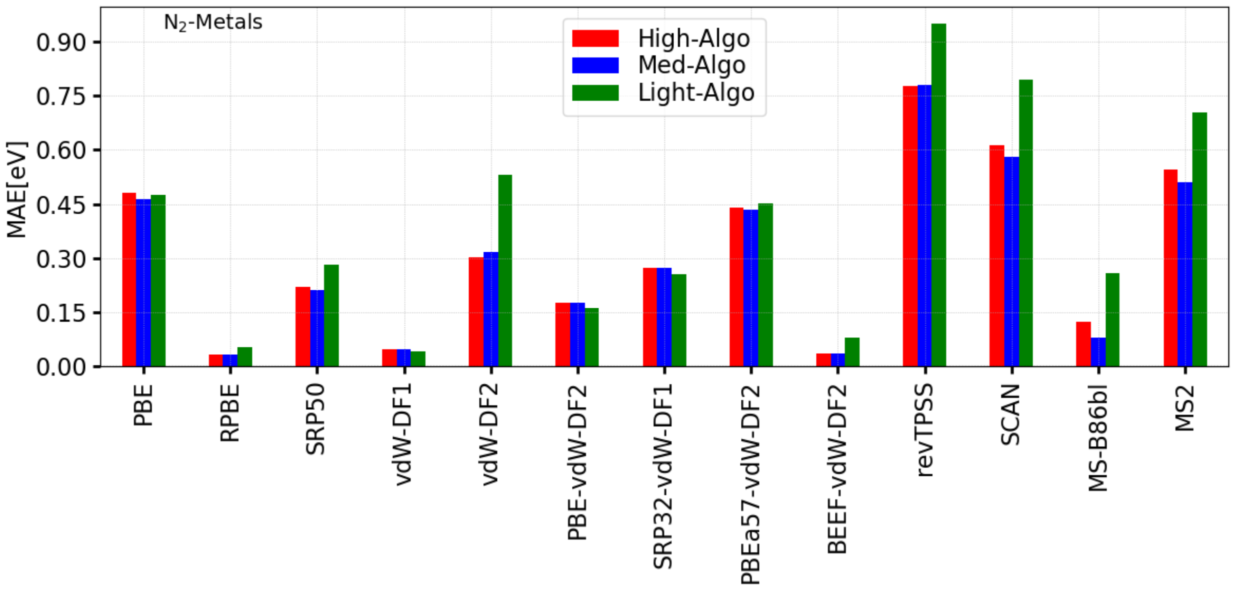


Fig. S19 – Bar plot of the mean absolute error obtained for $N_2$-metal surface systems for the three algorithms for all DFs tested.

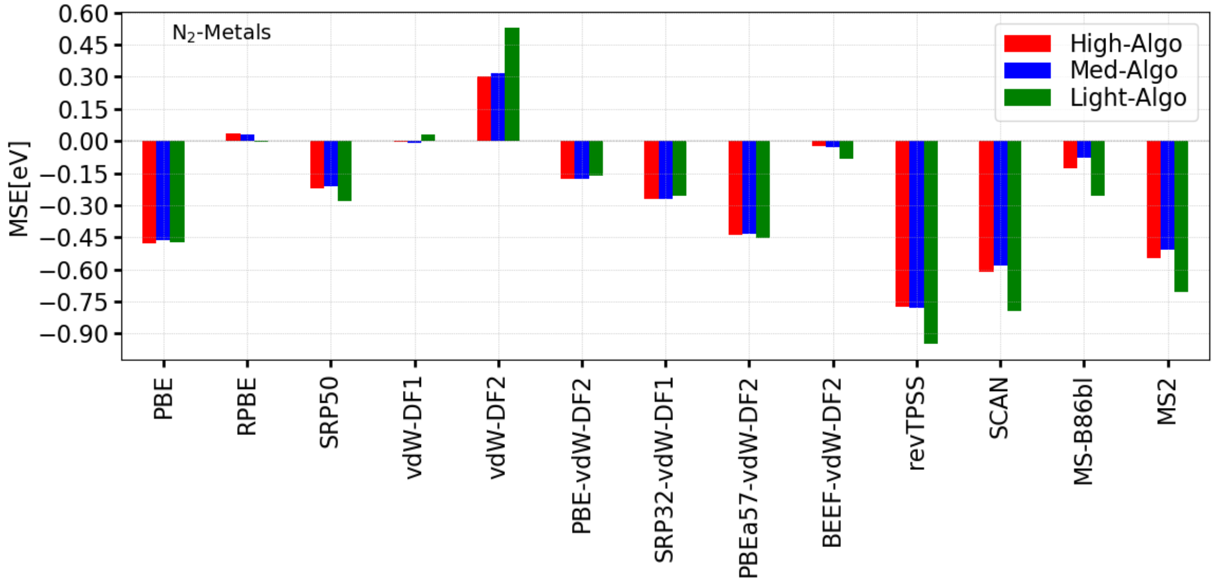


Fig. S20 – Bar plot of the mean signed error obtained for $N_2$-metal surface systems for the three algorithms for all DFs tested.

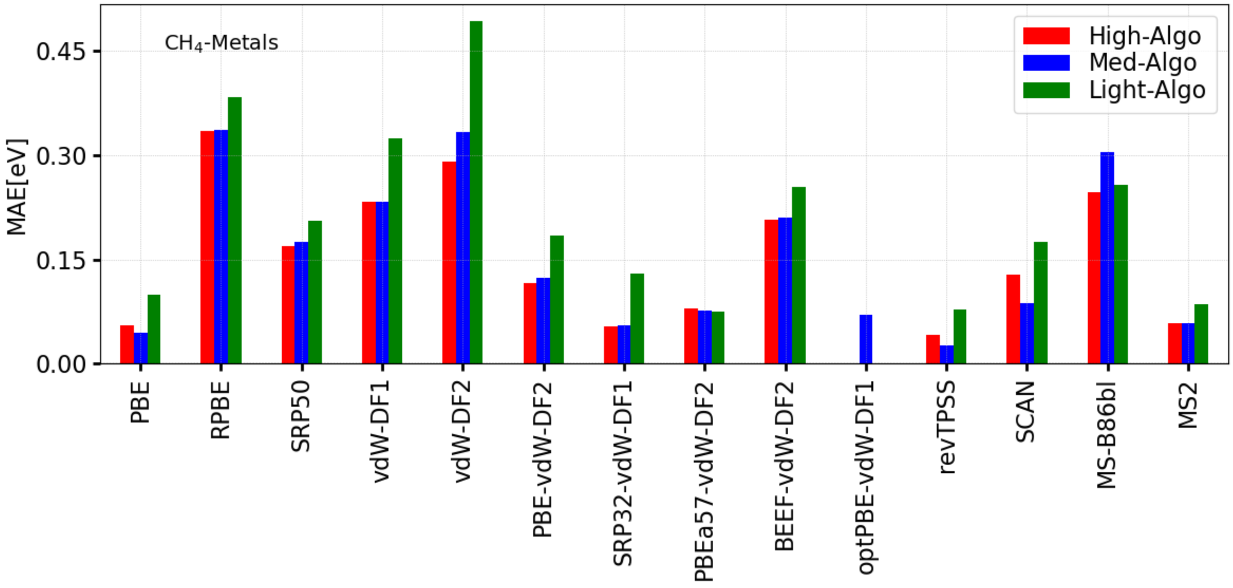


Fig. S21 – Bar plot of the mean absolute error obtained for $CH_4$-metal surface systems for the three algorithms for all DFs tested.

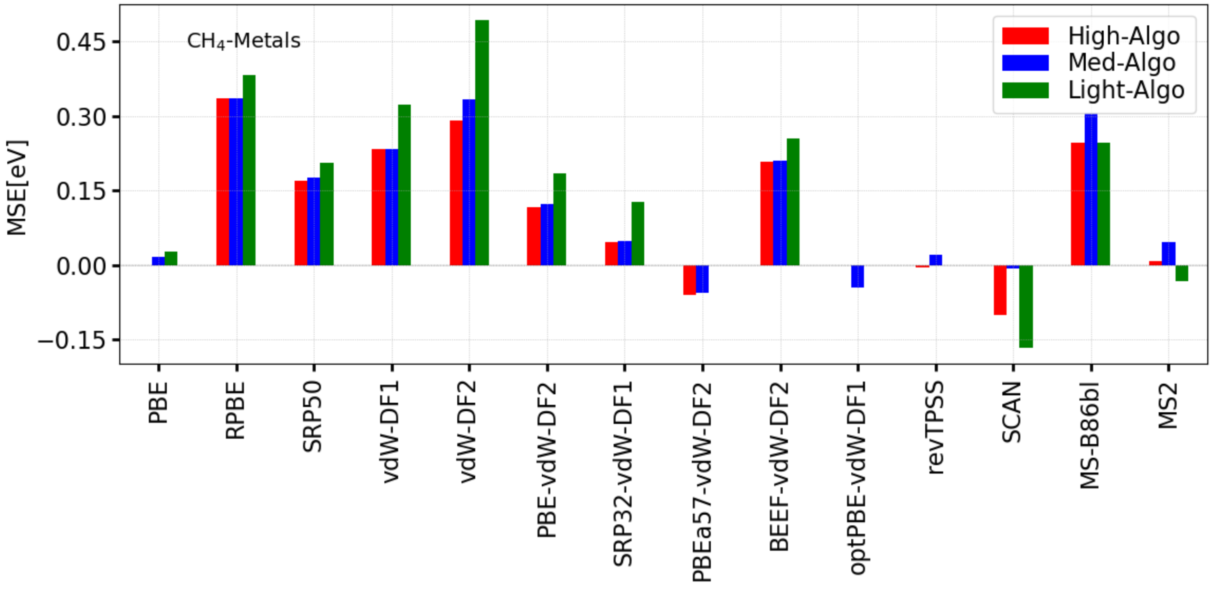


Fig. S22 – Bar plot of the mean signed error obtained for $CH_4$-metal surface systems for the three algorithms for all DFs tested.

# References

(1) Mallikarjun Sharada, S.; Bligaard, T.; Luntz, A. C.; Kroes, G.-J.; Nørskov, J. K. SBH10: A benchmark database of barrier heights on transition metal surfaces. *J. Phys. Chem. C* **2017**, *121*, 19807–19815.

(2) Díaz, C.; Pijper, E.; Olsen, R. A.; Busnengo, H. F.; Auerbach, D. J.; Kroes, G.-J. Chemically accurate simulation of a prototypical surface reaction: $H_2$ dissociation on Cu(111). *Science* **2009**, *326*, 832–834.

(3) Sementa, L.; Wijzenbroek, M.; Van Kolck, B. J.; Somers, M. F.; Al-Halabi, A.; Busnengo, H. F.; Olsen, R. A.; Kroes, G.-J.; Rutkowski, M.; Thewes, C., et al. Reactive scattering of $H_2$ from Cu (100): Comparison of dynamics calculations based on the specific reaction parameter approach to density functional theory with experiment. *J. Chem. Phys.* **2013**, *138*, 044708.

(4) Zhu, L.; Zhang, Y.; Zhang, L.; Zhou, X.; Jiang, B. Unified and transferable description of dynamics of $H_2$ dissociative adsorption on multiple copper surfaces via machine learning. *Phys. Chem. Chem. Phys.* **2020**, *22*, 13958–13964.

(5) Ghassemi, E. N.; Wijzenbroek, M.; Somers, M. F.; Kroes, G.-J. Chemically accurate simulation of dissociative chemisorption of $D_2$ on Pt(111). *Chem. Phys. Lett.* **2017**, *683*, 329–335.

(6) Ghassemi, E. N.; Smeets, E. W. F.; Somers, M. F.; Kroes, G.-J.; Groot, I. M.; Juurlink, L. B.; Füchsel, G. Transferability of the Specific Reaction Parameter Density Functional for $H_2$+ Pt(111) to $H_2$+ Pt(211). *J. Phys. Chem. C* **2019**, *123*, 2973–2986.

(7) Tchakoua, T.; Smeets, E. W.; Somers, M.; Kroes, G.-J. Toward a Specific Reaction Parameter Density Functional for $H_2$+ Ni(111): Comparison of Theory with Molecular Beam Sticking Experiments. *J. Phys. Chem. C* **2019**, *123*, 20420–20433.

(8) Smeets, E. W. F.; Kroes, G.-J. Performance of Made Simple Meta-GGA Functionals with rVV10 Nonlocal Correlation for $H_2$+ Cu(111), $D_2$+Ag(111), $H_2$+Au(111), and $D_2$+Pt(111). *J. Phys. Chem. C* **2021**, *125*, 8993–9010.

(9) Wijzenbroek, M.; Kroes, G. J. The effect of the exchange-correlation functional on $H_2$ dissociation on Ru(0001). *J. Chem. Phys.* **2014**, *140*, 084702.

(10) Shakouri, K.; Behler, J.; Meyer, J.; Kroes, G.-J. Accurate neural network description of surface phonons in reactive gas–surface dynamics: $N_2$+ Ru (0001). *J. Phys. Chem. Lett.* **2017**, *8*, 2131–2136.

(11) Nattino, F.; Migliorini, D.; Kroes, G.-J.; Dombrowski, E.; High, E. A.; Killelea, D. R.; Utz, A. L. Chemically accurate simulation of a polyatomic molecule-metal surface reaction. *J. Phys. Chem. Lett.* **2016**, *7*, 2402–2406.

(12) Guo, H.; Menzel, J. P.; Jackson, B. Quantum dynamics studies of the dissociative chemisorption of $CH_4$ on the steps and terraces of Ni(211). *J. Chem. Phys.* **2018**, *149*, 244704.

(13) Migliorini, D.; Chadwick, H.; Kroes, G.-J. Methane on a stepped surface: Dynamical insights on the dissociation of $CHD_3$ on Pt(111) and Pt(211). *J. Chem. Phys.* **2018**, *149*, 094701.

(14) Jackson, B. Direct and trapping-mediated pathways to dissociative chemisorption: $CH_4$ dissociation on Ir (111) with step defects. *J. Chem. Phys.* **2020**, *153*, 034704.

(15) Wijzenbroek, M. Ph.D. thesis, Leiden Institute of Chemistry, 2016.

(16) Salin, A. Theoretical study of hydrogen dissociative adsorption on the Cu(110) surface. *J. Chem. Phys.* **2006**, *124*, 104704.

(17) Wijzenbroek, M.; Klein, D. M.; Smits, B.; Somers, M. F.; Kroes, G.-J. Performance of a

non-local van der Waals density functional on the dissociation of $H_2$ on metal surfaces. *J. Phys. Chem. A* **2015**, *119*, 12146–12158.

(18) Smeets, E. W. F.; Voss, J.; Kroes, G.-J. Specific Reaction Parameter Density Functional Based on the Meta-Generalized Gradient Approximation: Application to $H_2$+Cu(111) and $H_2$+Ag(111). *J. Phys. Chem. A* **2019**, *123*, 5395–5406.

(19) Smeets, E. W. F.; Kroes, G.-J. Designing new SRP density functionals including non-local vdW-DF2 correlation for $H_2$+Cu(111) and their transferability to $H_2$+Ag(111), Au(111) and Pt(111). *Phys. Chem. Chem. Phys.* **2021**, *23*, 7875–7901.

(20) Nave, S.; Tiwari, A. K.; Jackson, B. Methane dissociation and adsorption on Ni(111), Pt(111), Ni(100), Pt(100), and Pt (110)-(1× 2): energetic study. *J. Chem. Phys.* **2010**, *132*, 054705.

(21) Nave, S.; Tiwari, A. K.; Jackson, B. Dissociative chemisorption of methane on ni and pt surfaces: mode-specific chemistry and the effects of lattice motion. *J. Phys. Chem. A* **2014**, *118*, 9615–9631.

(22) Nattino, F.; Migliorini, D.; Bonfanti, M.; Kroes, G.-J. Methane dissociation on Pt(111): Searching for a specific reaction parameter density functional. *J. Chem. Phys.* **2016**, *144*, 044702.

(23) Guo, H.; Jackson, B. Mode-selective chemistry on metal surfaces: The dissociative chemisorption of CH4 on Pt (111). *J. Chem. Phys.* **2016**, *144*, 184709.

(24) Migliorini, D.; Chadwick, H.; Nattino, F.; Gutiérrez-González, A.; Dombrowski, E.; High, E. A.; Guo, H.; Utz, A. L.; Jackson, B.; Beck, R. D.; Kroes, G.-J. Surface reaction barriometry: methane dissociation on flat and stepped transition-metal surfaces. *J. Phys. Chem. Lett.* **2017**, *8*, 4177–4182.

(25) Moiraghi, R.; Lozano, A.; Busnengo, H. F. Theoretical study of the dissociative adsorption of methane on Ir(111): The role of steps and surface distortions at high temperatures. *J. Phys. Chem. C* **2016**, *120*, 3946–3954.
